# Supplementary material for: A systematic atlas of chaperome deregulation topologies across the human cancer landscape
Source: PLoS Comput Biol. 2018 Jan 2;14(1):e1005890. doi: 10.1371/journal.pcbi.1005890 (PMC5766242; doi:10.1371/journal.pcbi.1005890)
Supplement: S1 Fig — Correlation between GSA-scores [33] and Meta-PCA T-statistic values derived using Limma linear modelling on all TCGA cancer groups considered in this study indicates overall correlation (cor = 0.61). (PDF) [file pcbi.1005890.s001.pdf]

Figure S1

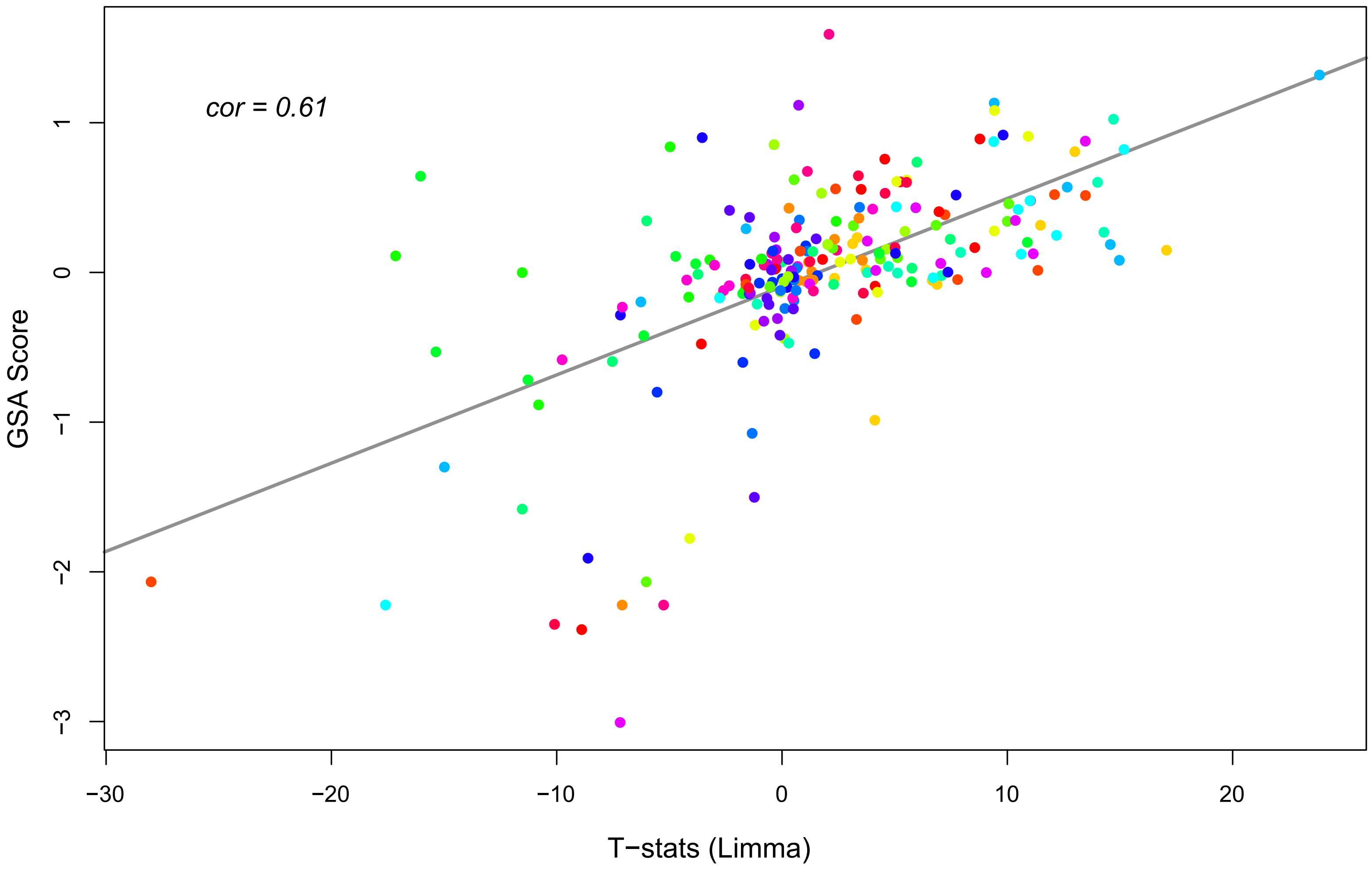

TCGA Cancer Group:

- Bladder Urothelial Carcinoma (BLCA)
- Breast invasive carcinoma (BRCA)
- Cervical squamous cell carcinoma and endocervical adenocarcinoma (CESC)
- Cholangiocarcinoma (CHOL)
- Esophageal carcinoma (ESCA)
- Glioblastoma multiforme (GBM)
- Head and Neck squamous cell carcinoma (HNSC)
- Kidney Chromophobe (KICH)
- Kidney renal clear cell carcinoma (KIRC)
- Kidney renal papillary cell carcinoma (KIRP)
- Liver hepatocellular carcinoma (LIHC)
- Lung adenocarcinoma (LUAD)
- Lung squamous cell carcinoma (LUSC)
- Pancreatic adenocarcinoma (PAAD)
- Pheochromocytoma and Paraganglioma (PCPG)
- Prostate adenocarcinoma (PRAD)
- Sarcoma (SARC)
- Skin Cutaneous Melanoma (SKCM)
- Stomach adenocarcinoma (STAD)
- Thyroid carcinoma (THCA)
- Thymoma (THYM)
- Uterine Corpus Endometrial Carcinoma (UCEC)
