# Supplementary figures and images for: A systematic atlas of chaperome deregulation topologies across the human cancer landscape
Source: PLoS Comput Biol. 2018 Jan 2;14(1):e1005890. doi: 10.1371/journal.pcbi.1005890 (PMC5766242; doi:10.1371/journal.pcbi.1005890)

## Figure S2

# A

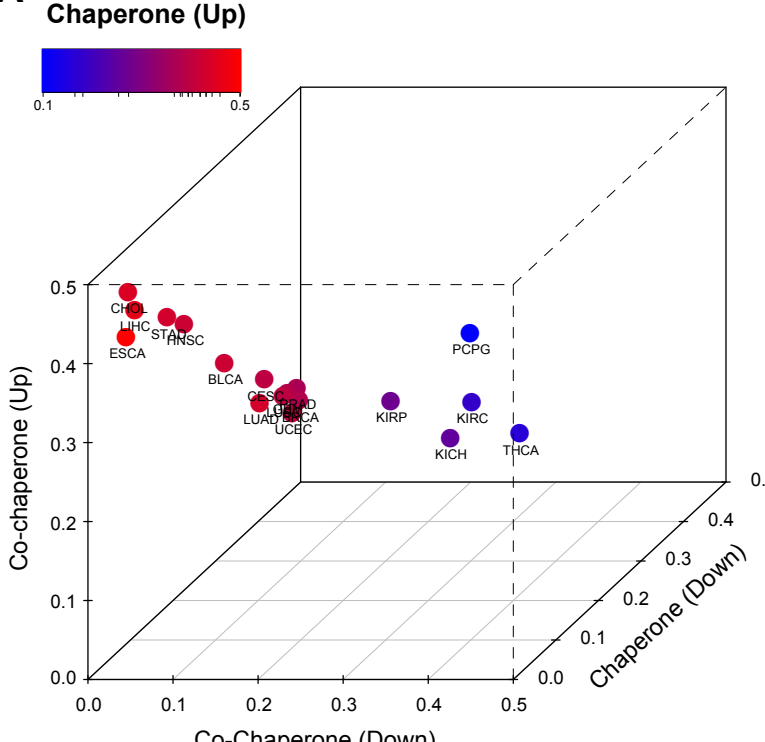

# C

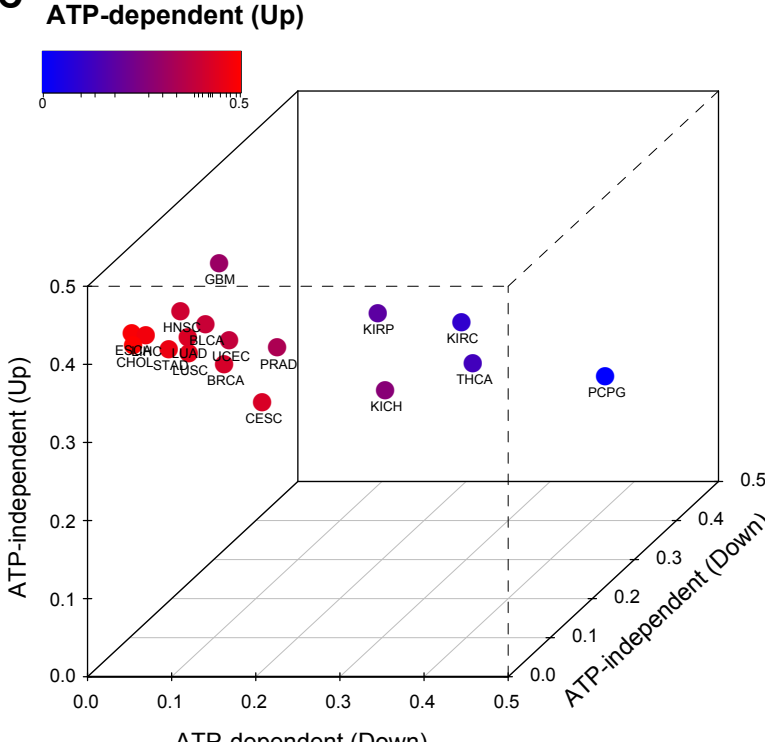

# B

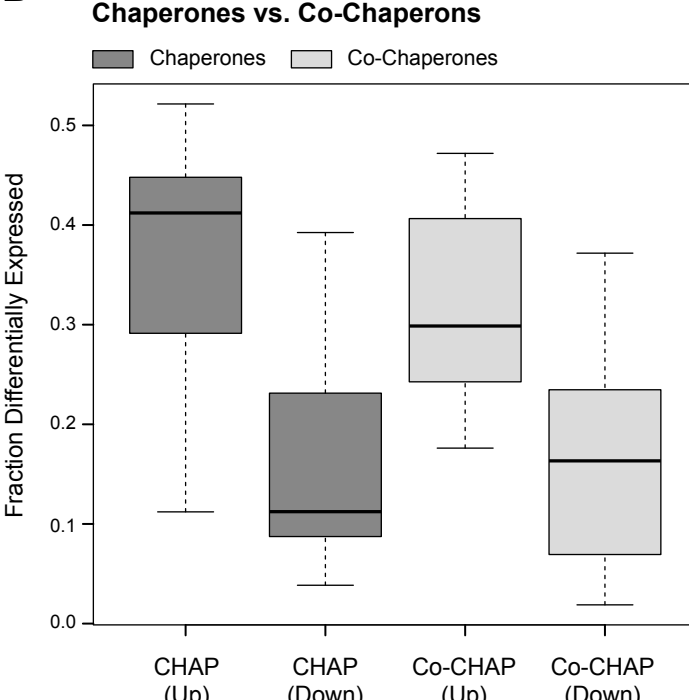

# D

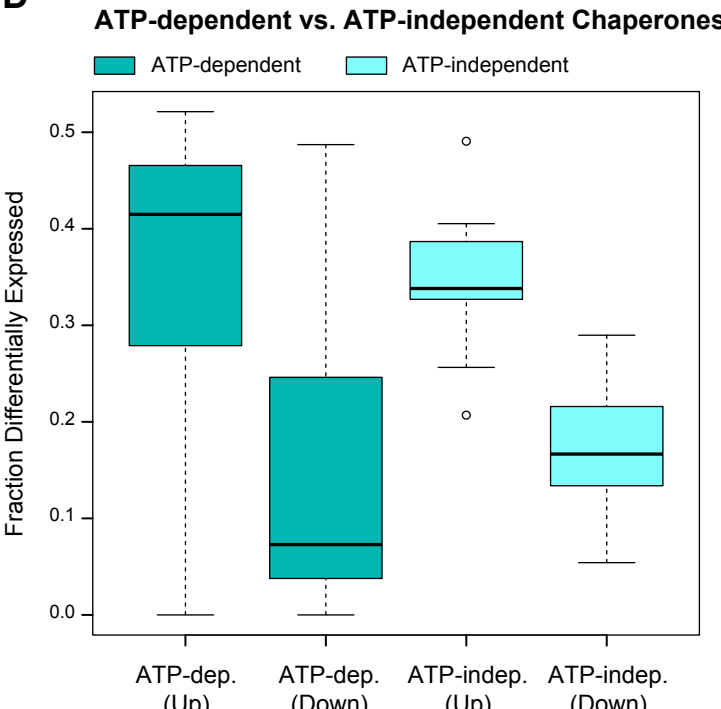

Supplement: S2 Fig — Analysis of differential cancer gene expression of chaperome functional subsets, comparing chaperones and co-chaperones as well as ATP-dependent and ATP-independent chaperones [4]. See also Fig 3. A. Comparing upregulation and downregulation of gene expression of chaperones and co-chaperones using GSA reveals a general upregulation of chaperones and co-chaperones in cancer, with preferential upregulation of chaperones. Colour code indicates chaperone up-regulation of gene expression. Axes represent chaperone downregulation, co-chaperone upregulation and downregulation of gene expression. B. Box-and-whisker plots highlight fractions of differentially expressed genes in each chaperome subset for all TCGA cancers assessed, based on A. Differentially expressed genes in each set were obtained by linear modelling (Limma package in R) and considering genes with p value < 0.5 following Benjamini-Hochberg correction. Box boundaries, 25% and 75% quartiles; middle horizontal line, median; whiskers, quartile boundaries for values beyond 1.5 times the interquartile range; small circle, outlier. C. Assessing differential expression of ATP-dependent (n = 50) vs. ATP-independent (n = 38) chaperones highlights a preferential upregulation of ATP-dependent chaperones across TCGA cancers D. Box plots (drawn as in B.) show fractions of differentially expressed genes in the two sets of ATP-dependent and ATP-independent chaperones for all TCGA cancers assessed, based on C. (PDF) [file pcbi.1005890.s002.pdf]
