# Supplementary material for: A systematic atlas of chaperome deregulation topologies across the human cancer landscape
Source: PLoS Comput Biol. 2018 Jan 2;14(1):e1005890. doi: 10.1371/journal.pcbi.1005890 (PMC5766242; doi:10.1371/journal.pcbi.1005890)
Supplement: S3 Fig — Chaperome gene expression shifts between healthy and cancer (pp. 1–22) or AD, PD and HD (pp. 23–25) tissue biopsy datasets are quantified by Meta-PCA and visualized in context by plotting resulting M-scores on polar maps as in Fig 6. Blue (healthy) and red (disease) lines represent means across all samples for each disease. Halos represent confidence interval at the 90% quantile range (5%–95%). (PDF) [file pcbi.1005890.s003.pdf]

Figure S3

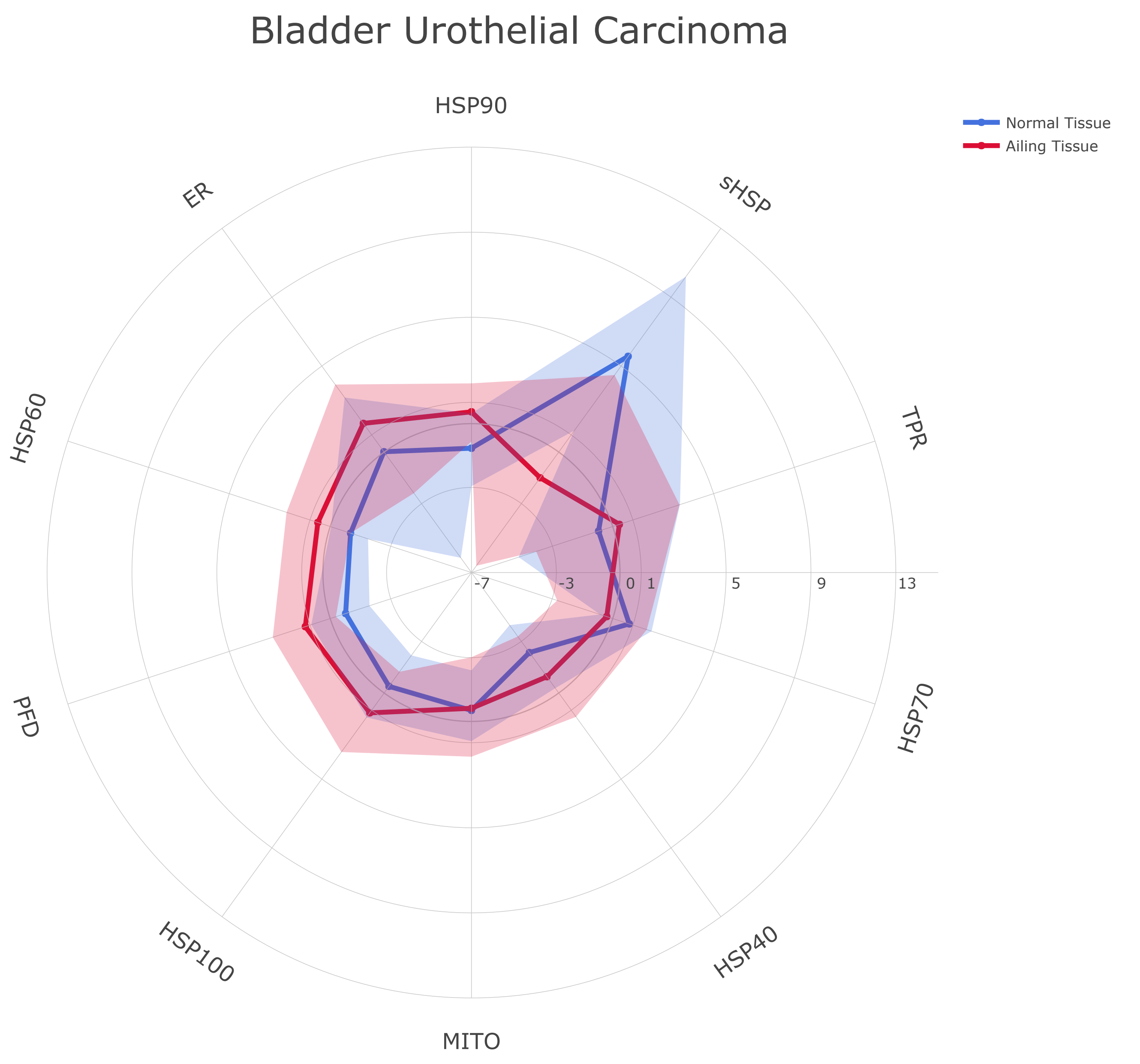
Bladder Urothelial Carcinoma
HSP90
sHSP
TPR
HSP70
HSP40
MITO
HSP100
PFD
HSP60
ER
-7
-3
0
1
5
9
13
Normal Tissue
Ailing Tissue

Breastinvasivecarcinoma

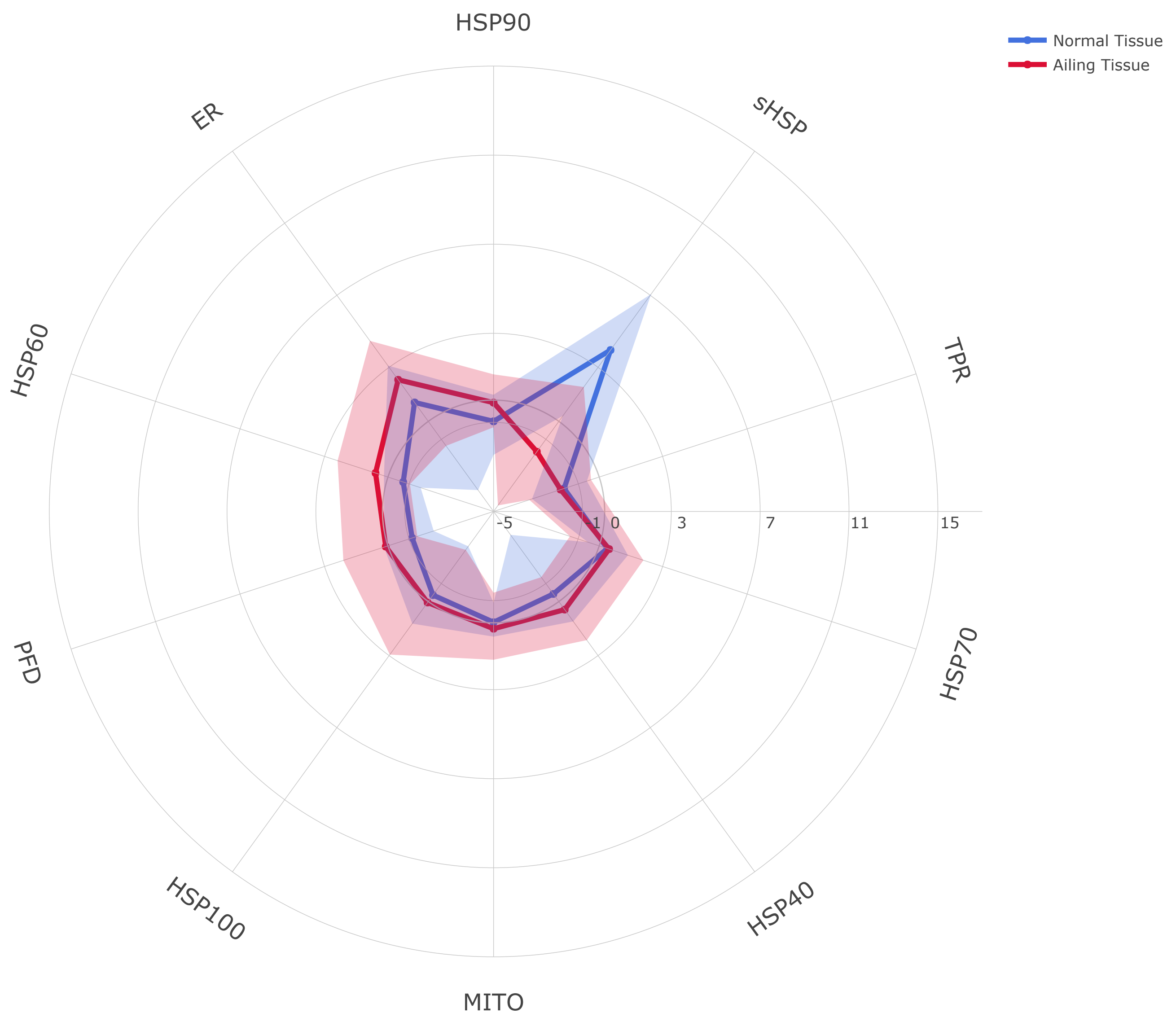

»

Cervicalsquamouscellcarcinomaandendocervicaladenocarcinoma

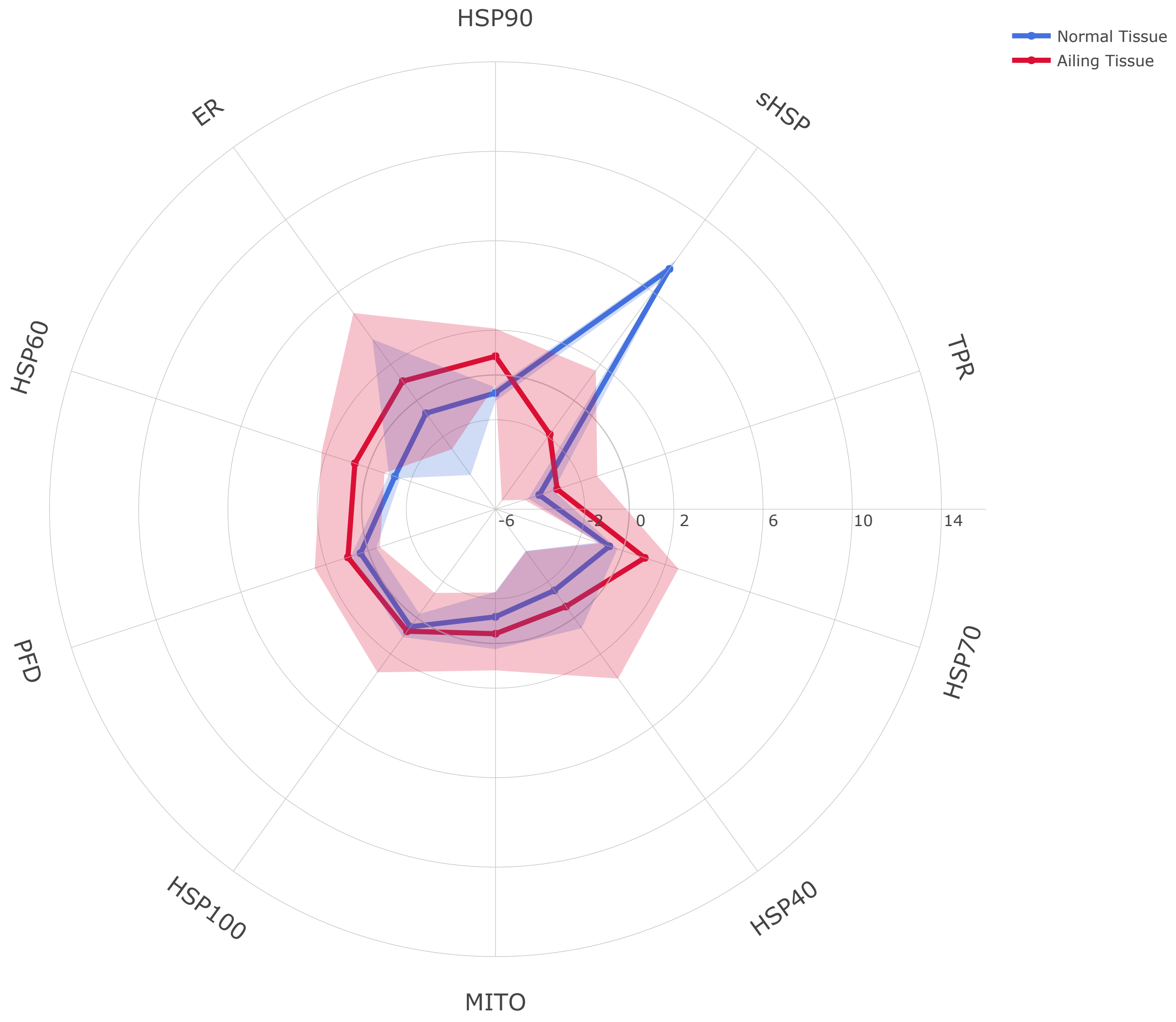

»

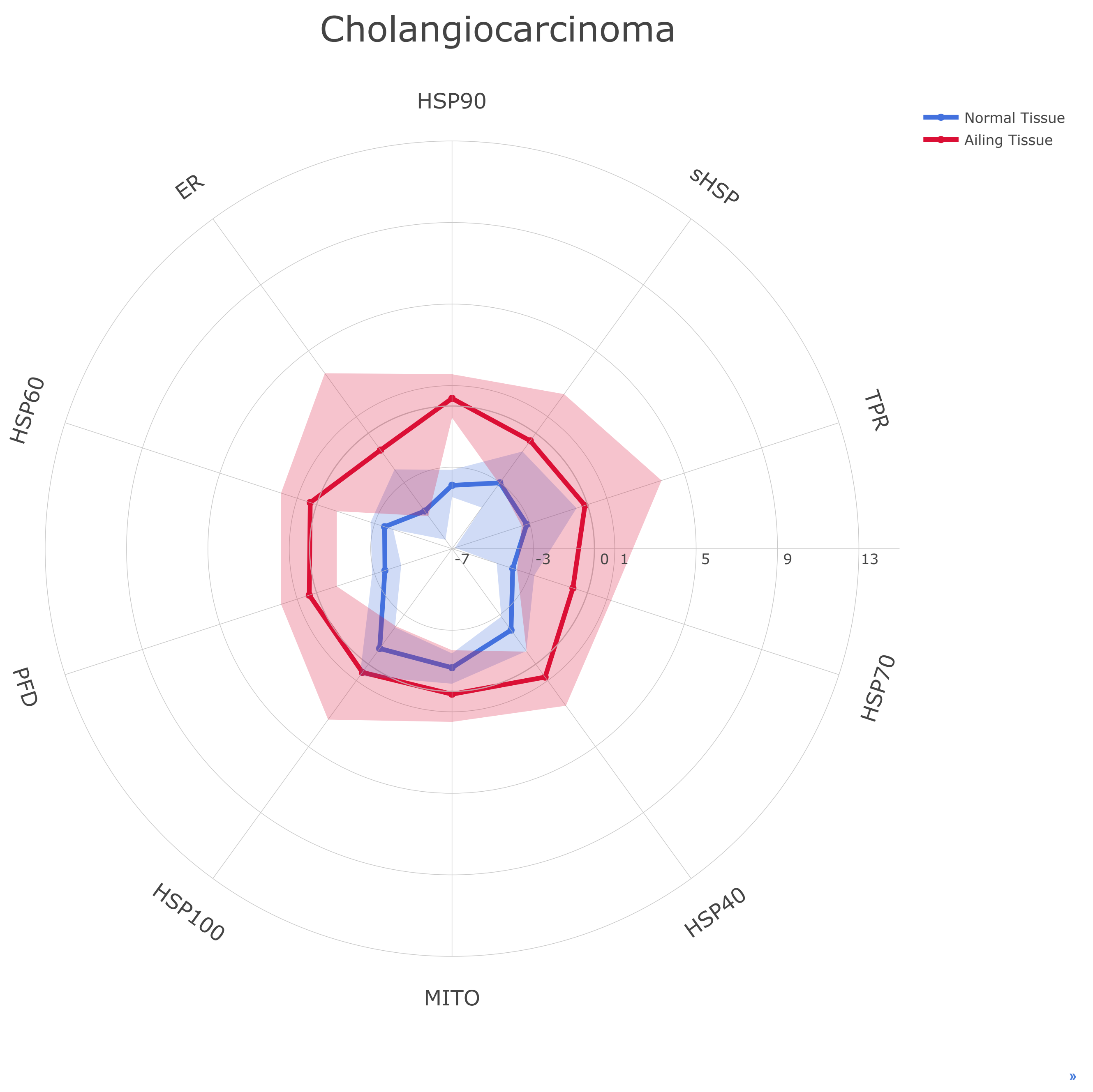
Cholangiocarcinoma
HSP90
sHSP
TPR
HSP70
HSP40
MITO
HSP100
PFD
HSP60
ER
-7
-3
0
1
5
9
13
Normal Tissue
Ailing Tissue

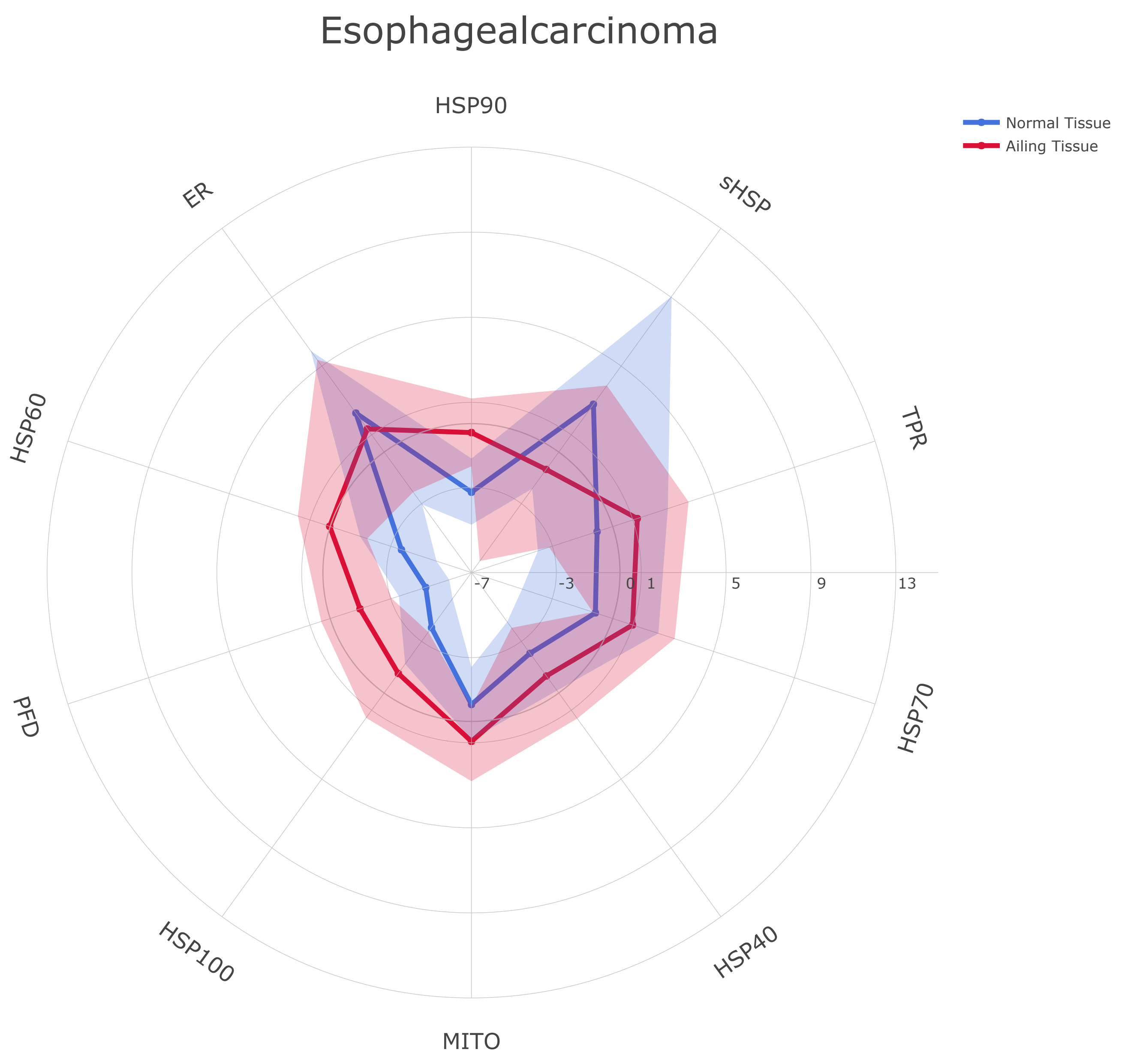
Esophagealcarcinoma
HSP90
sHSP
TPR
HSP70
HSP40
MITO
HSP100
PFD
HSP60
ER
-7
-3
0
1
5
9
13
Normal Tissue
Ailing Tissue

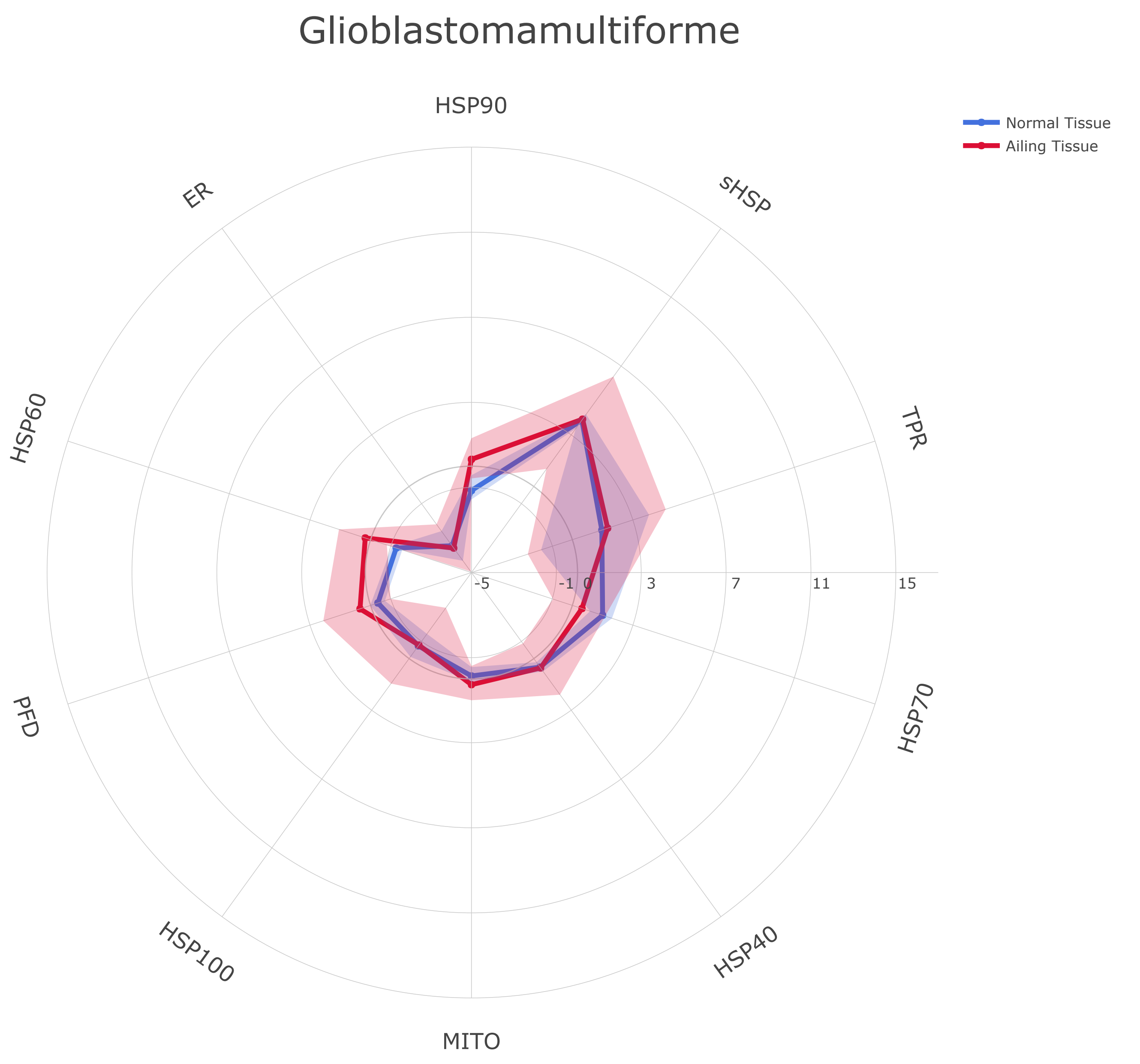
Glioblastomamultiforme
Normal Tissue
Ailing Tissue
HSP90
sHSP
TPR
HSP70
HSP40
MITO
HSP100
PFD
HSP60
ER
-5
-1
0
3
7
11
15

Headand Necksquamouscellcarcinoma

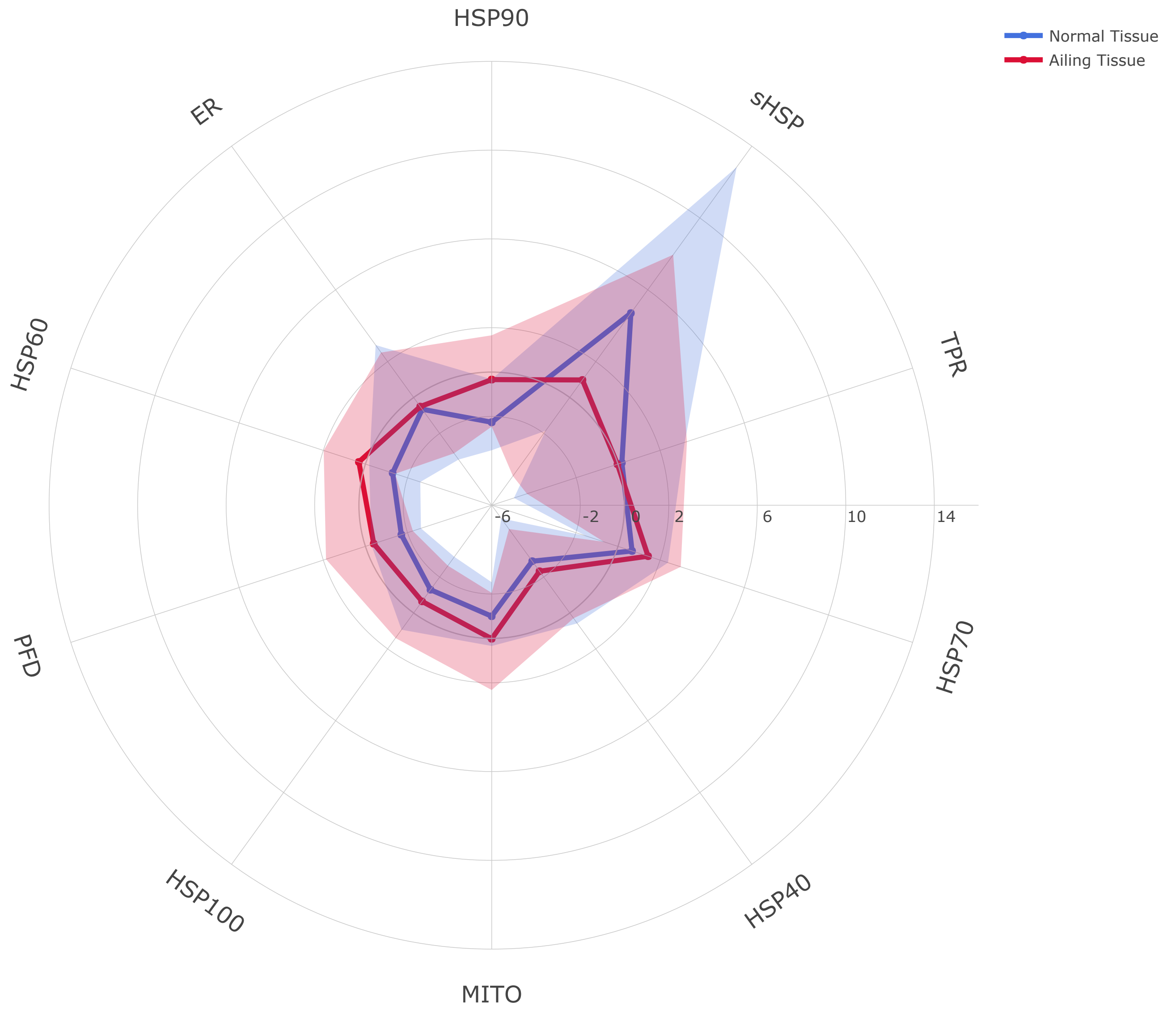

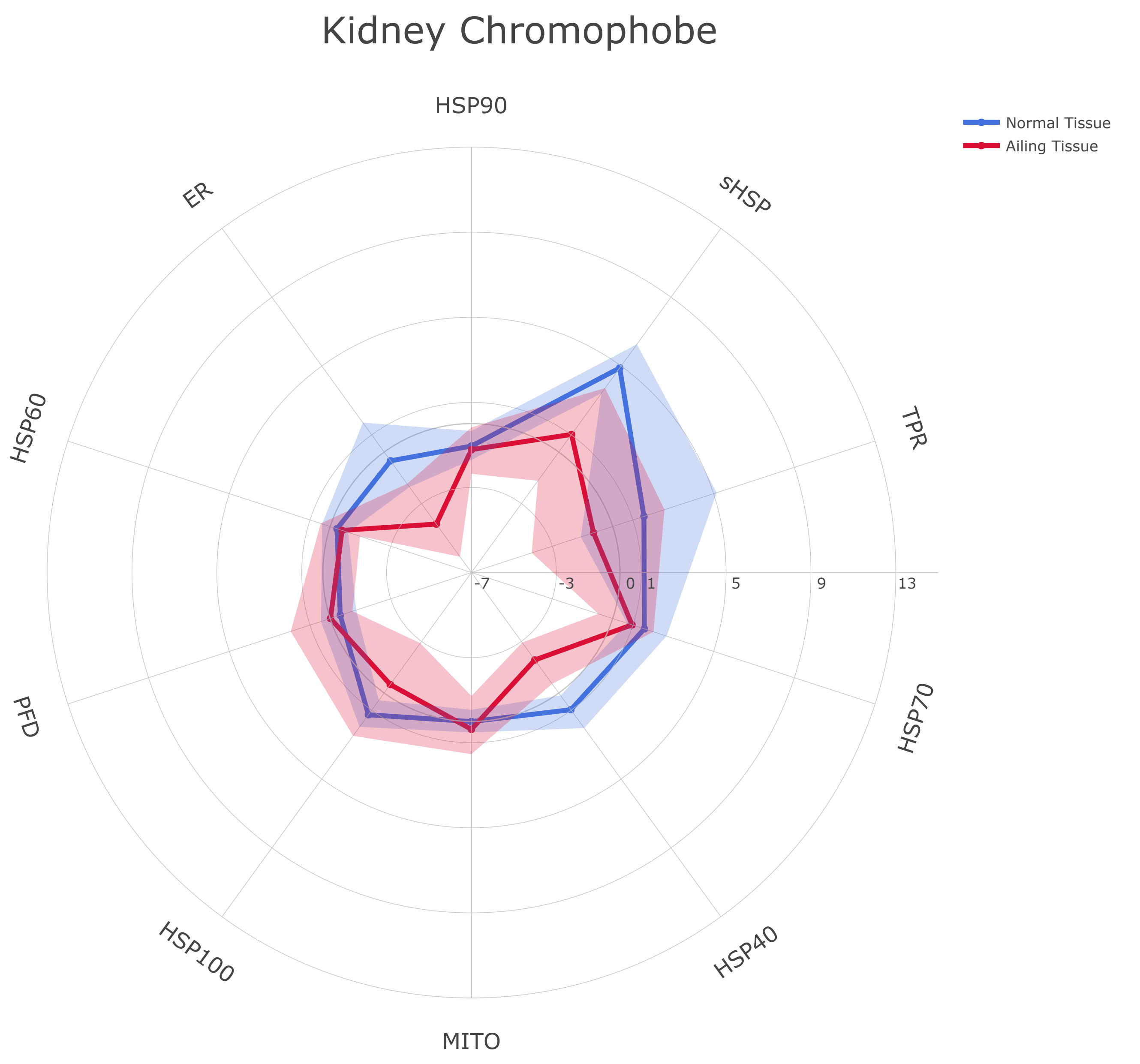

Kidney Chromophobe
HSP90
sHSP
TPR
HSP70
HSP40
MITO
HSP100
PFD
HSP60
ER
-7
-3
0
1
5
9
13
Normal Tissue
Ailing Tissue

»

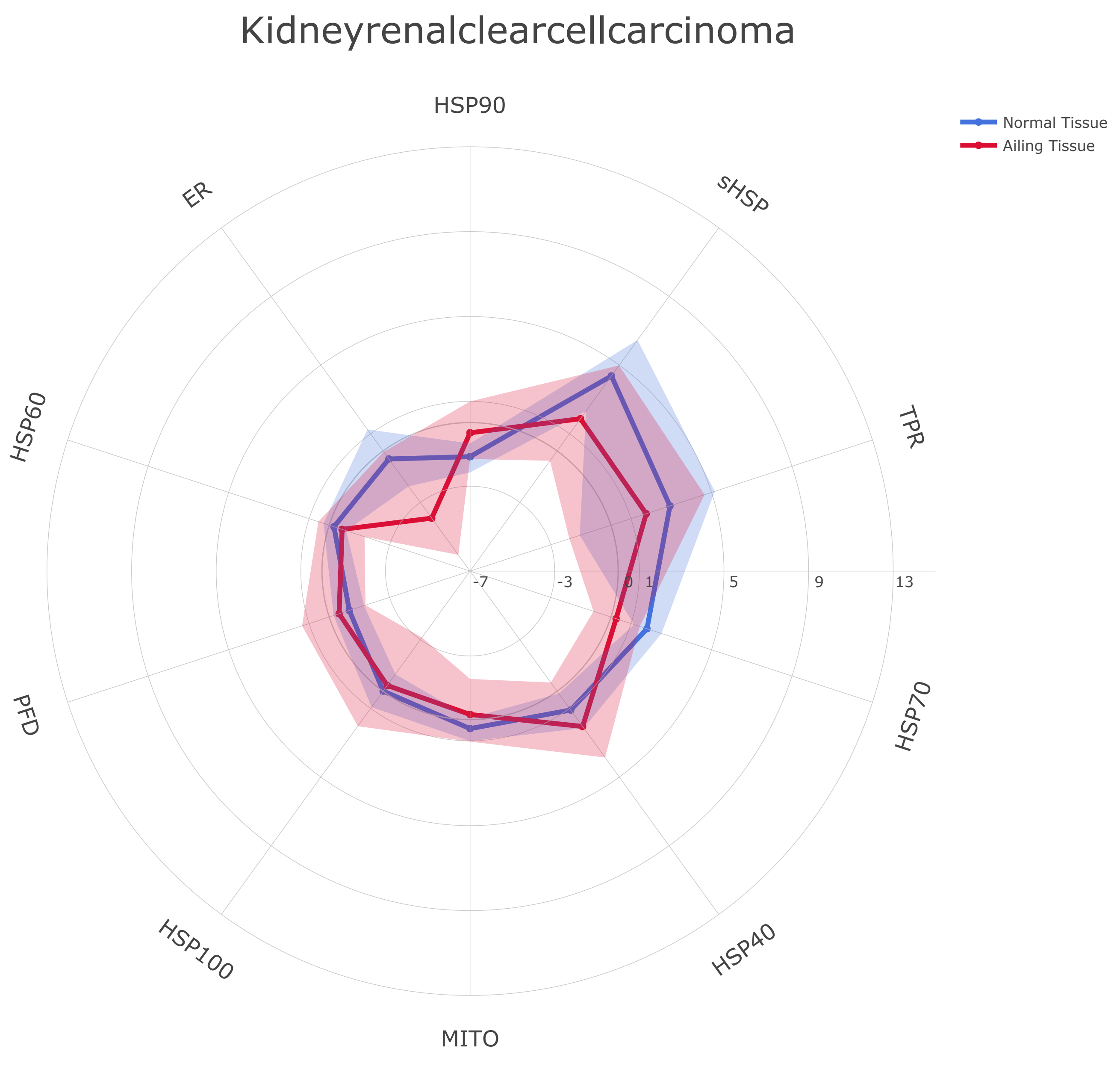
Kidneyrenalclearcellcarcinoma
HSP90
sHSP
TPR
HSP70
HSP40
MITO
HSP100
PFD
HSP60
ER
-7
-3
0
1
5
9
13
Normal Tissue
Ailing Tissue

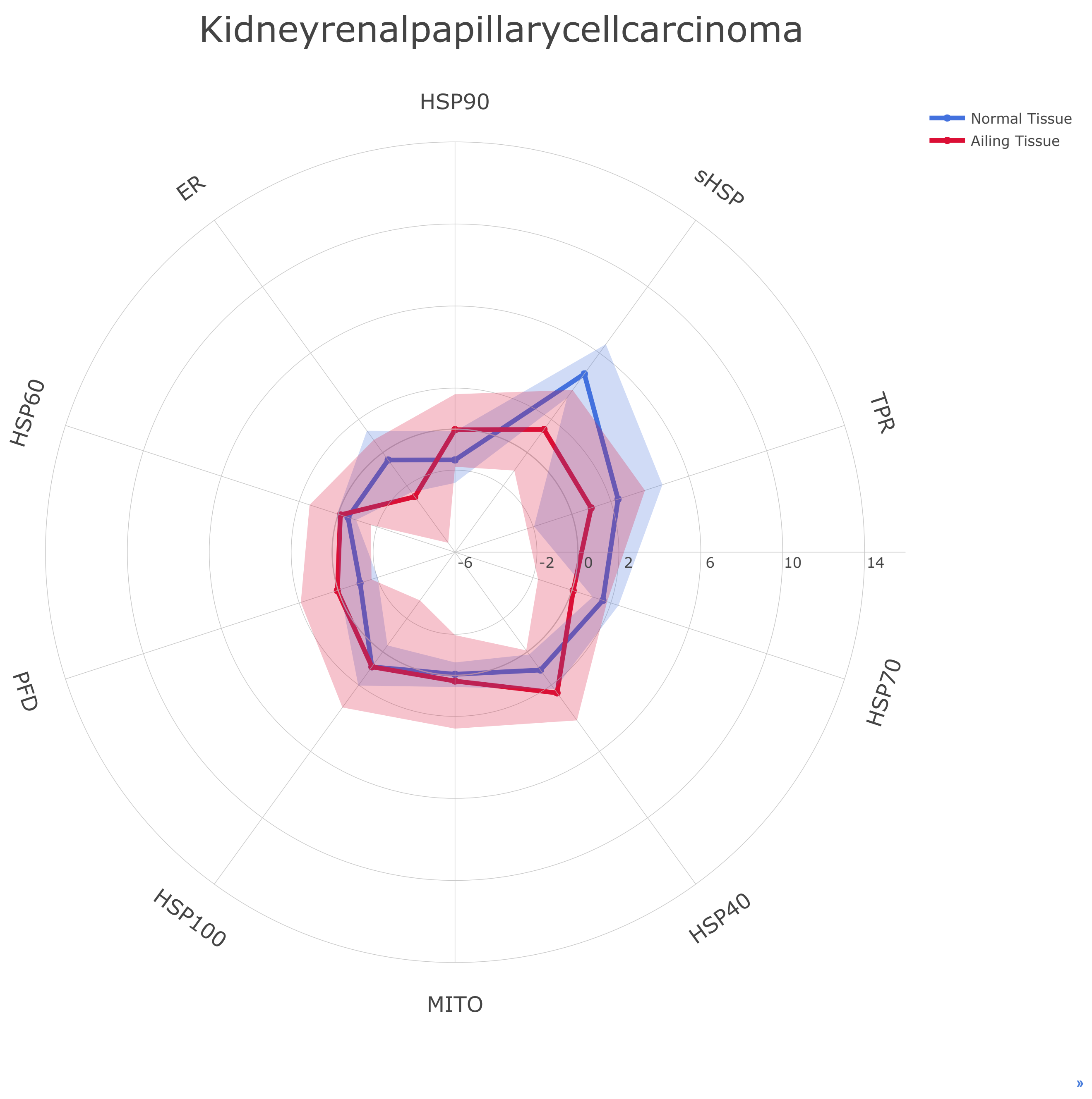

Kidneyrenalpapillarycellcarcinoma
HSP90
sHSP
TPR
HSP70
HSP40
MITO
HSP100
PFD
HSP60
ER
Normal Tissue
Ailing Tissue
-6
-2
0
2
6
10
14
»

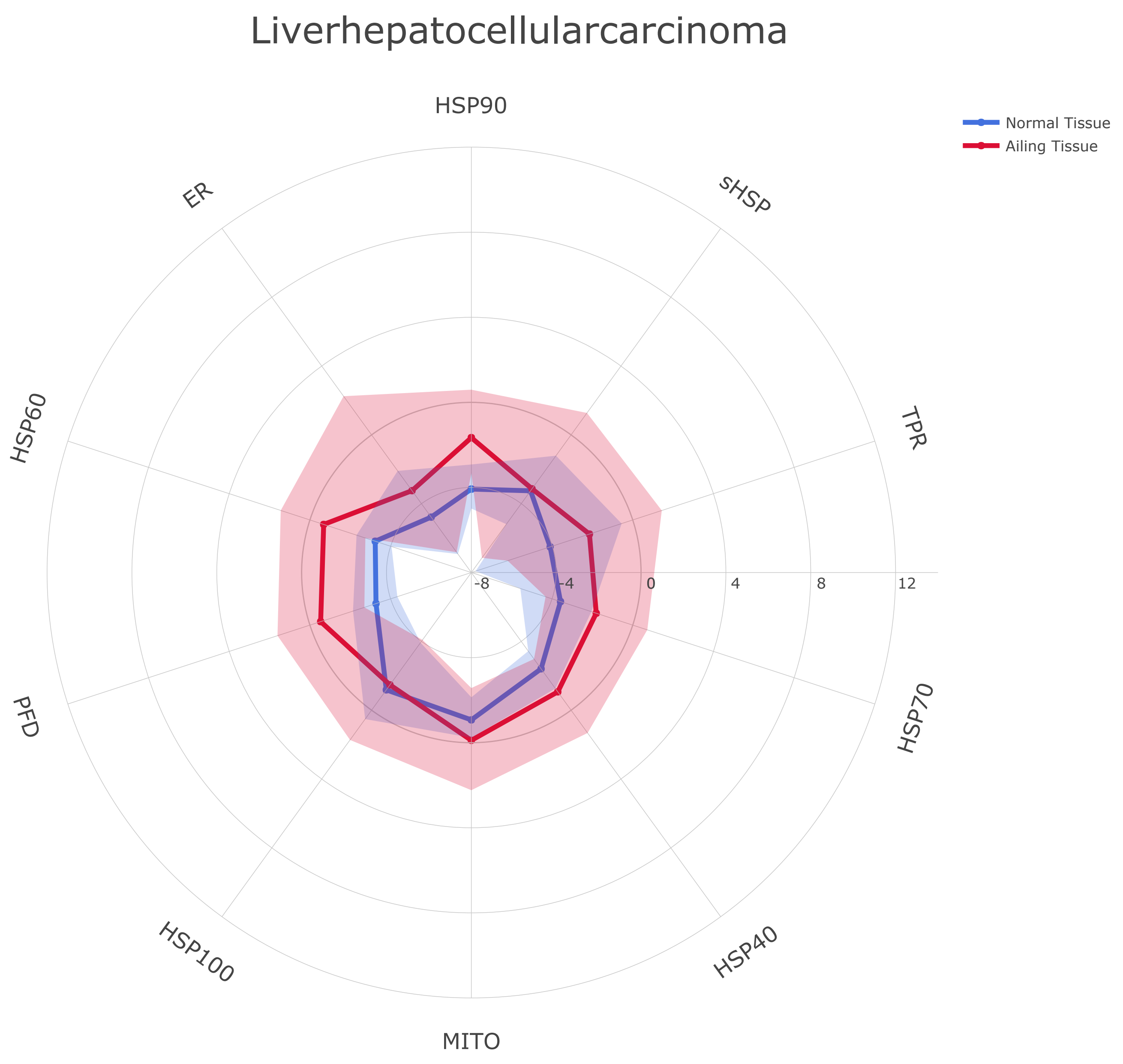

Liverhepatocellularcarcinoma
HSP90
sHSP
TPR
HSP70
HSP40
MITO
HSP100
PFD
HSP60
ER
-8
-4
0
4
8
12
Normal Tissue
Ailing Tissue

»

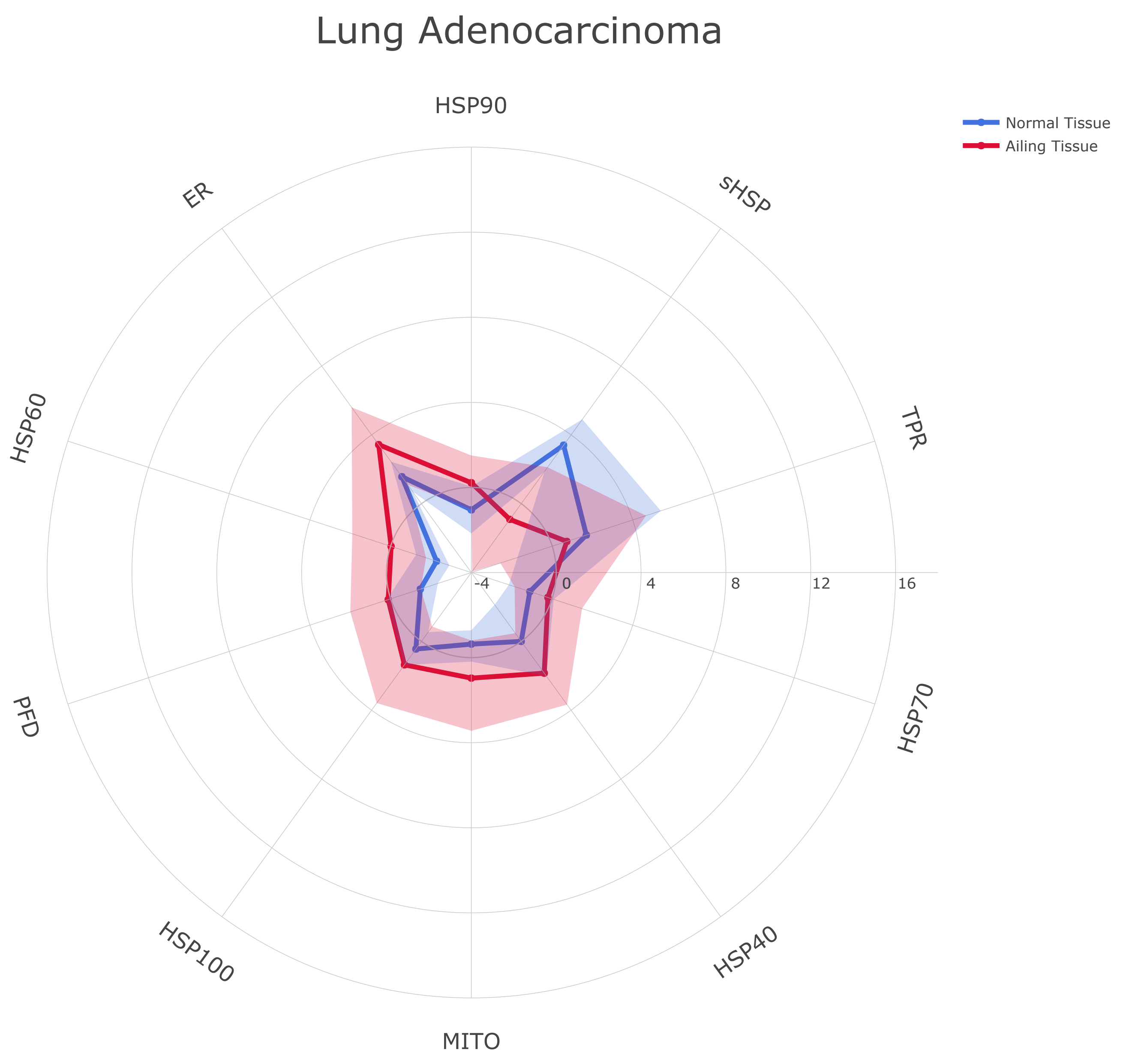

Lung Adenocarcinoma
HSP90
sHSP
TPR
HSP70
HSP40
MITO
HSP100
PFD
HSP60
ER
-4
0
4
8
12
16
Normal Tissue
Ailing Tissue

»

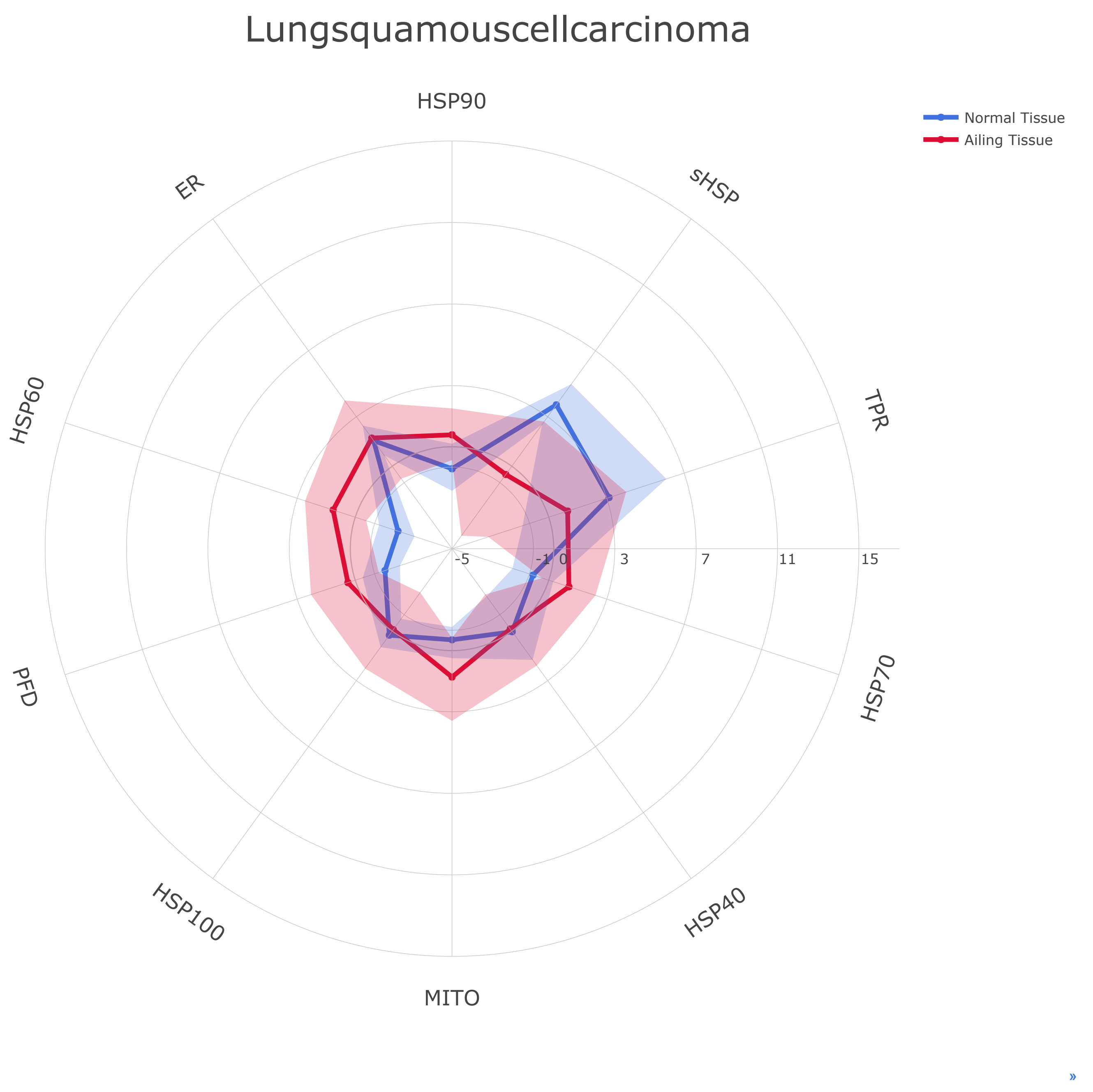

Lungsquamouscellcarcinoma
HSP90
sHSP
TPR
HSP70
HSP40
MITO
HSP100
PFD
HSP60
ER
Normal Tissue
Ailing Tissue
-5
-1
0
3
7
11
15

Pancreaticadenocarcinoma

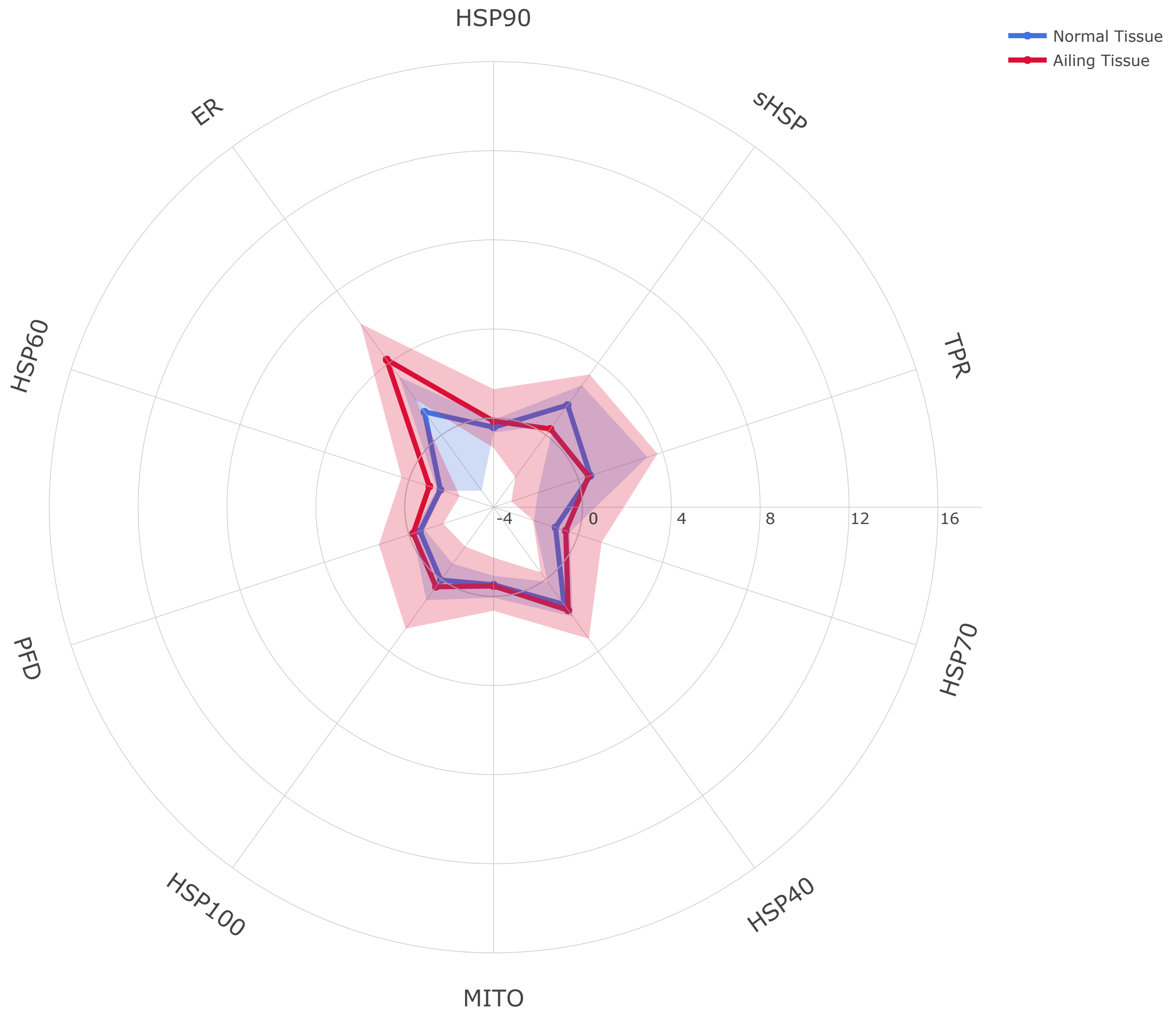

Pheochromocytomaand Paraganglioma

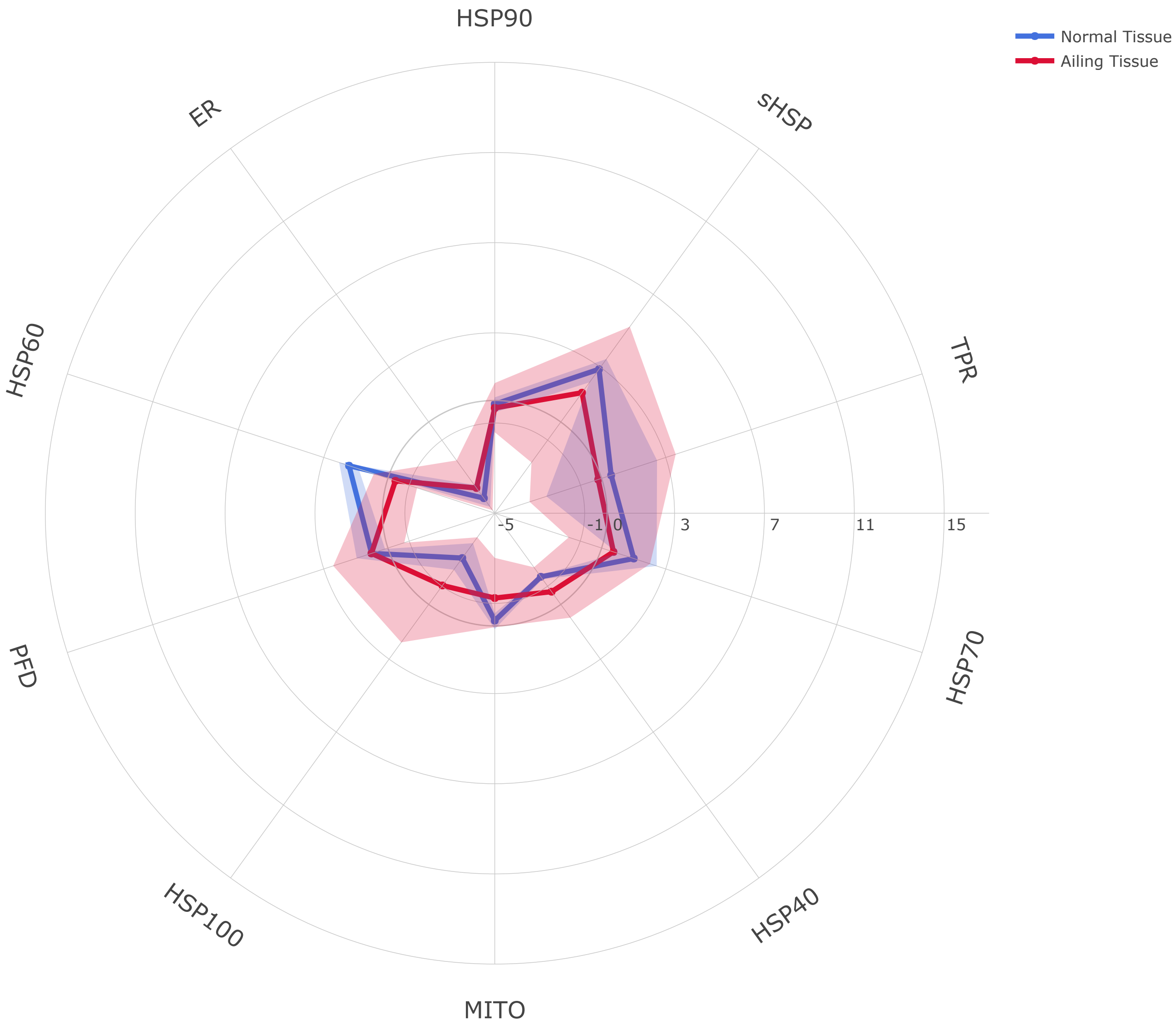

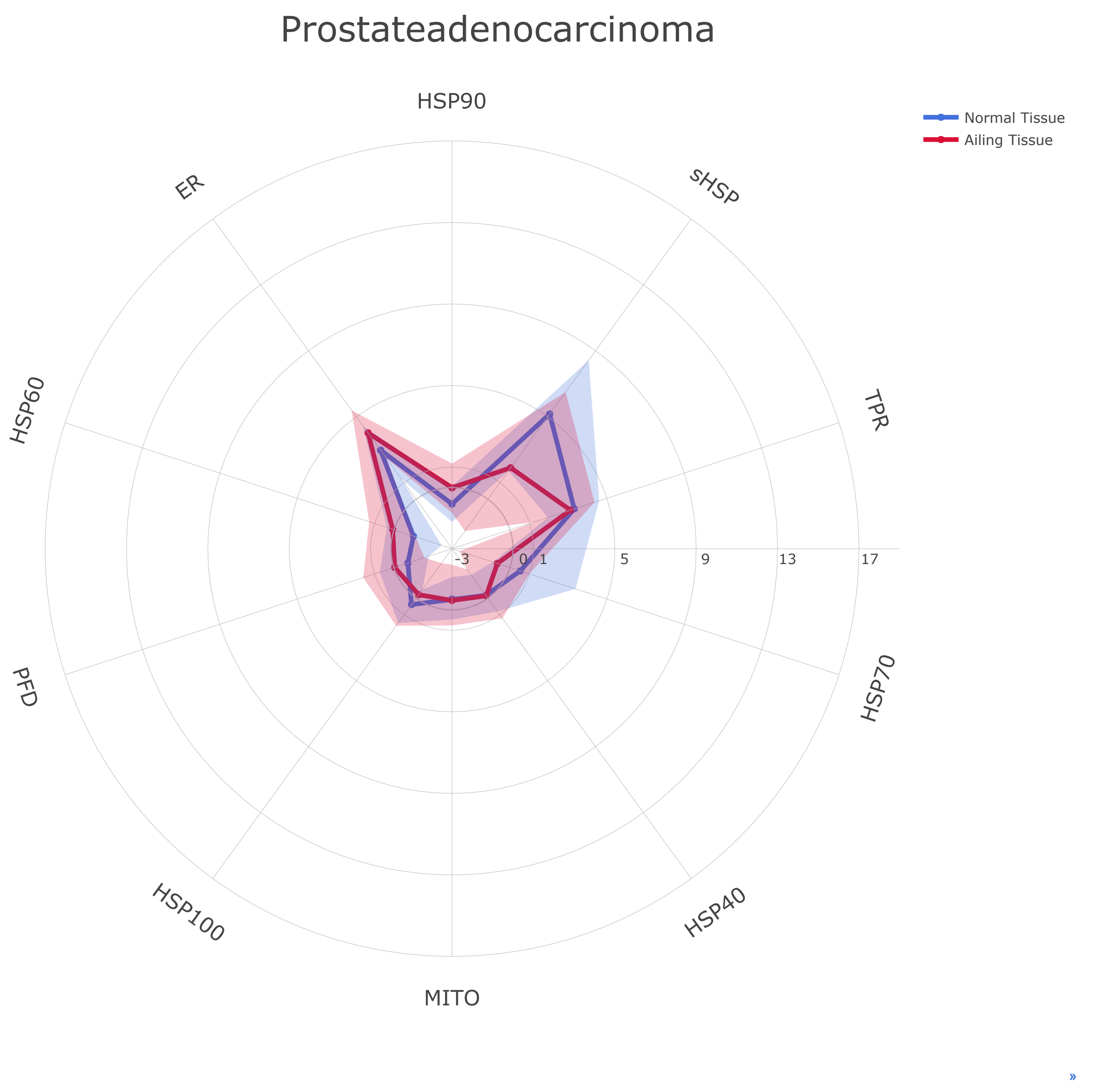

Prostateadenocarcinoma
Normal Tissue
Ailing Tissue
HSP90
sHSP
TPR
HSP70
HSP40
MITO
HSP100
PFD
HSP60
ER
-3
0
1
5
9
13
17

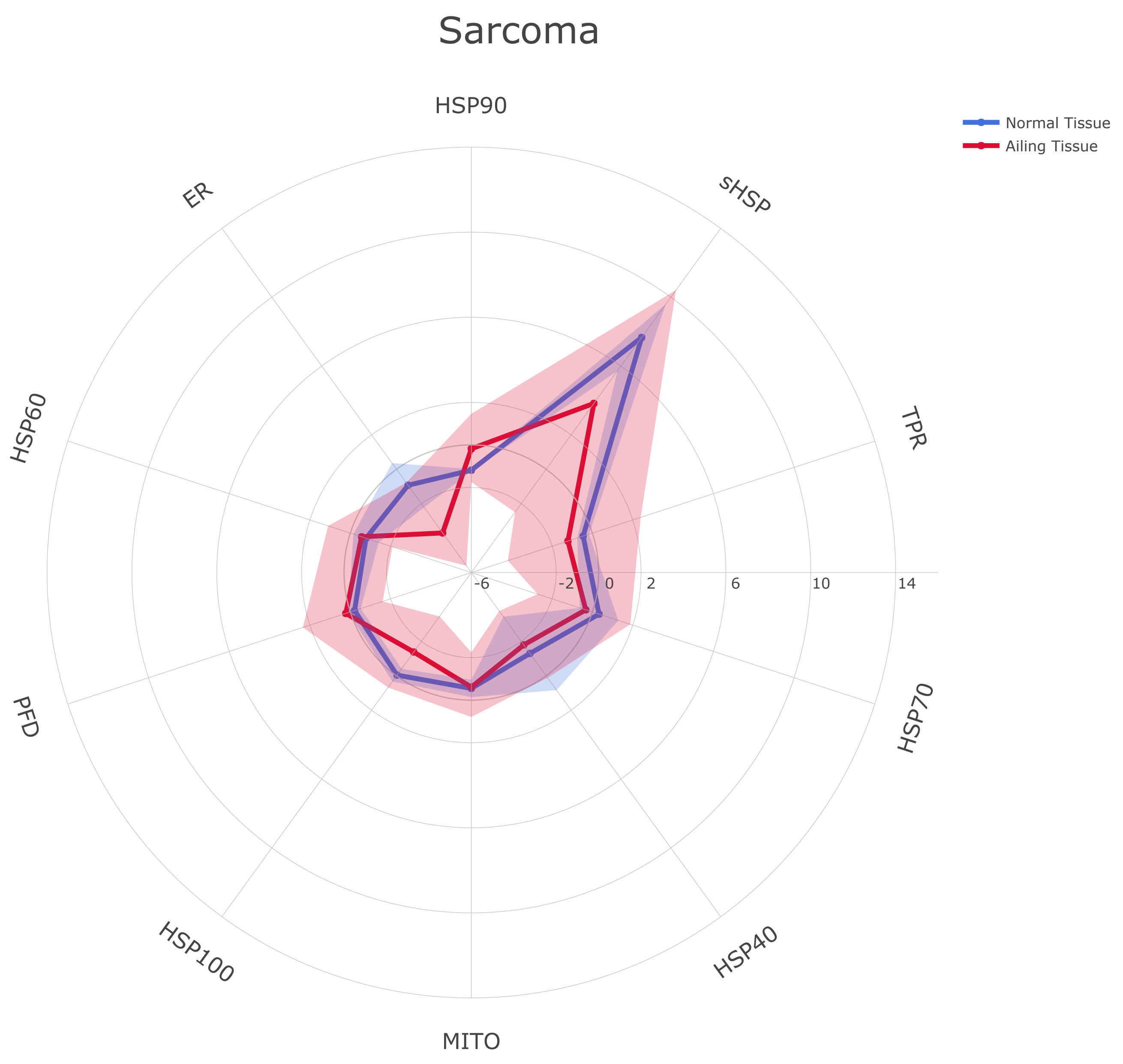
Sarcoma
HSP90
sHSP
TPR
HSP70
HSP40
MITO
HSP100
PFD
HSP60
ER
-6
-2
0
2
6
10
14
Normal Tissue
Ailing Tissue

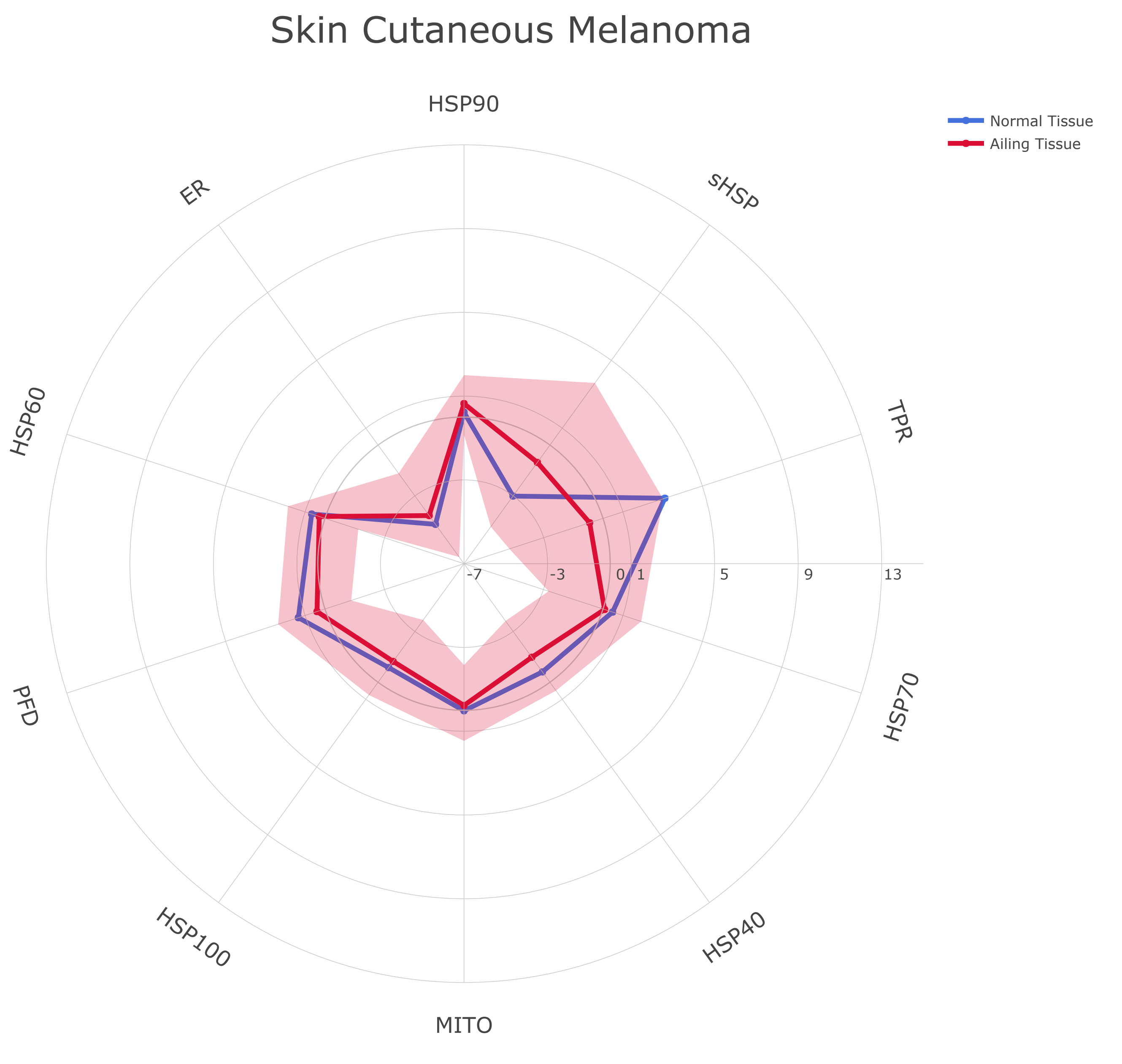
Skin Cutaneous Melanoma
HSP90
sHSP
TPR
HSP70
HSP40
MITO
HSP100
PFD
HSP60
ER
-7
-3
0
1
5
9
13
Normal Tissue
Ailing Tissue

Stomachadenocarcinoma

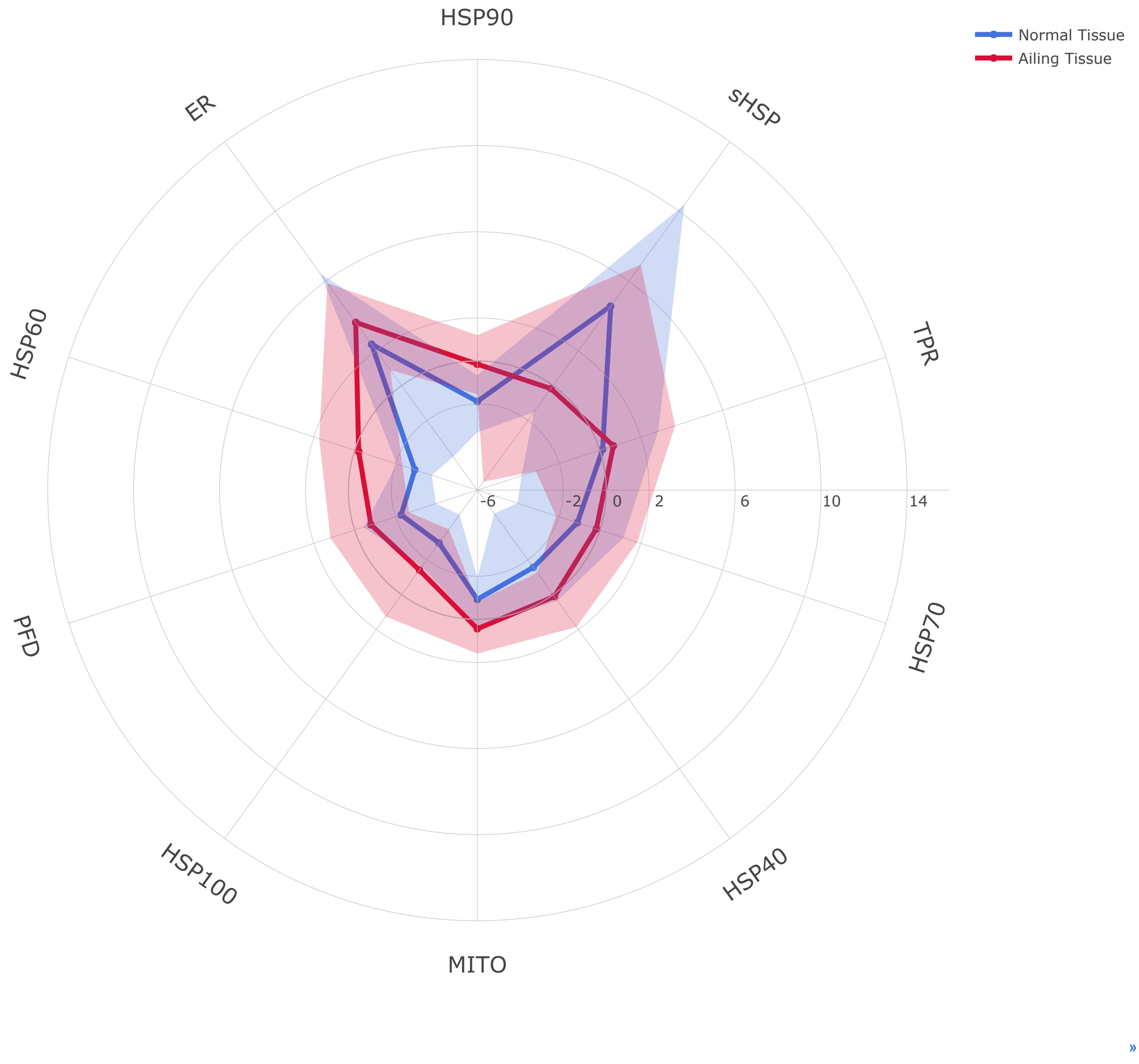

Thymoma

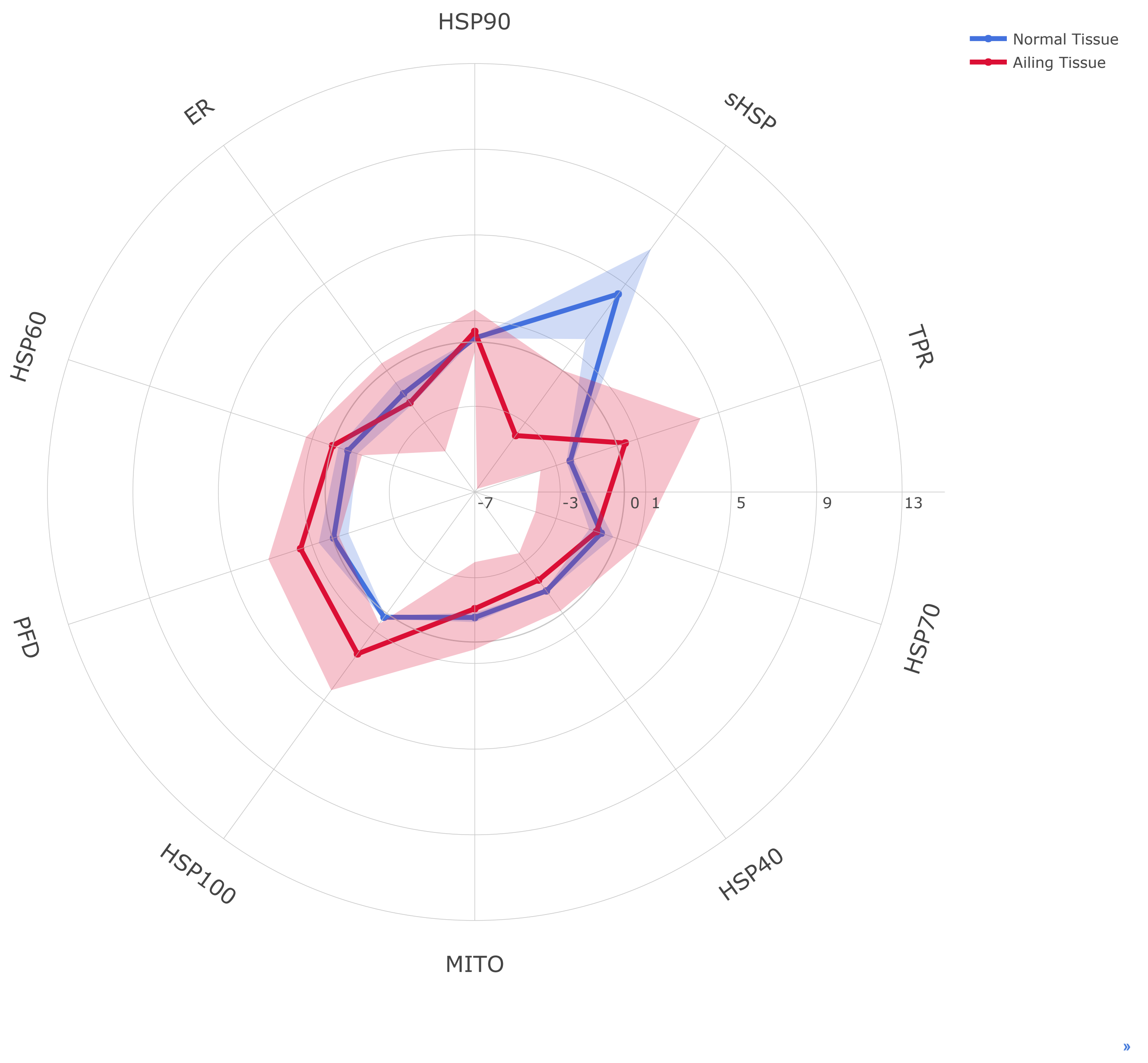

HSP90
sHSP
TPR
HSP70
HSP40
MITO
HSP100
PFD
HSP60
ER
-7
-3
0
1
5
9
13
Normal Tissue
Ailing Tissue
»

Thyroidcarcinoma

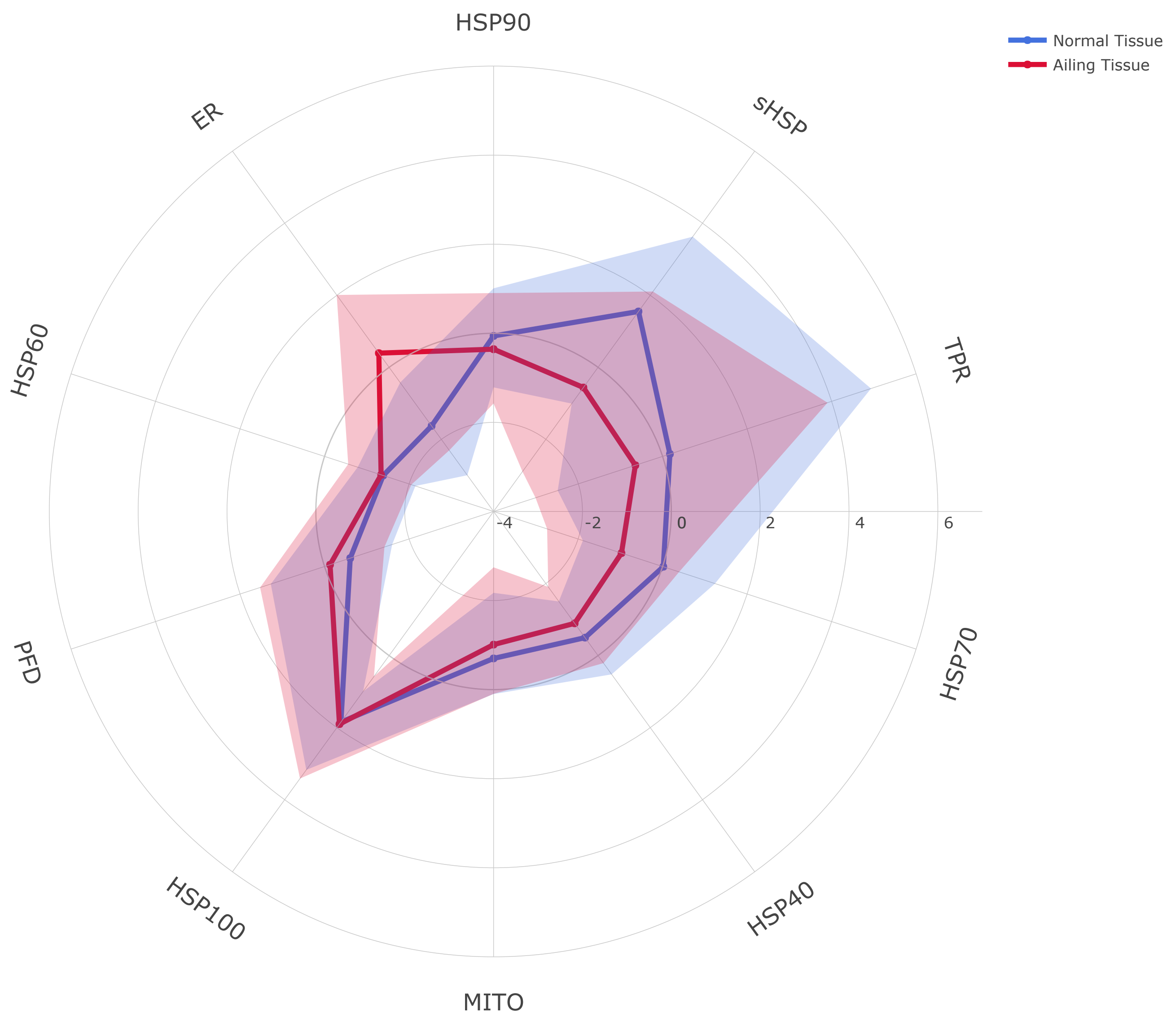

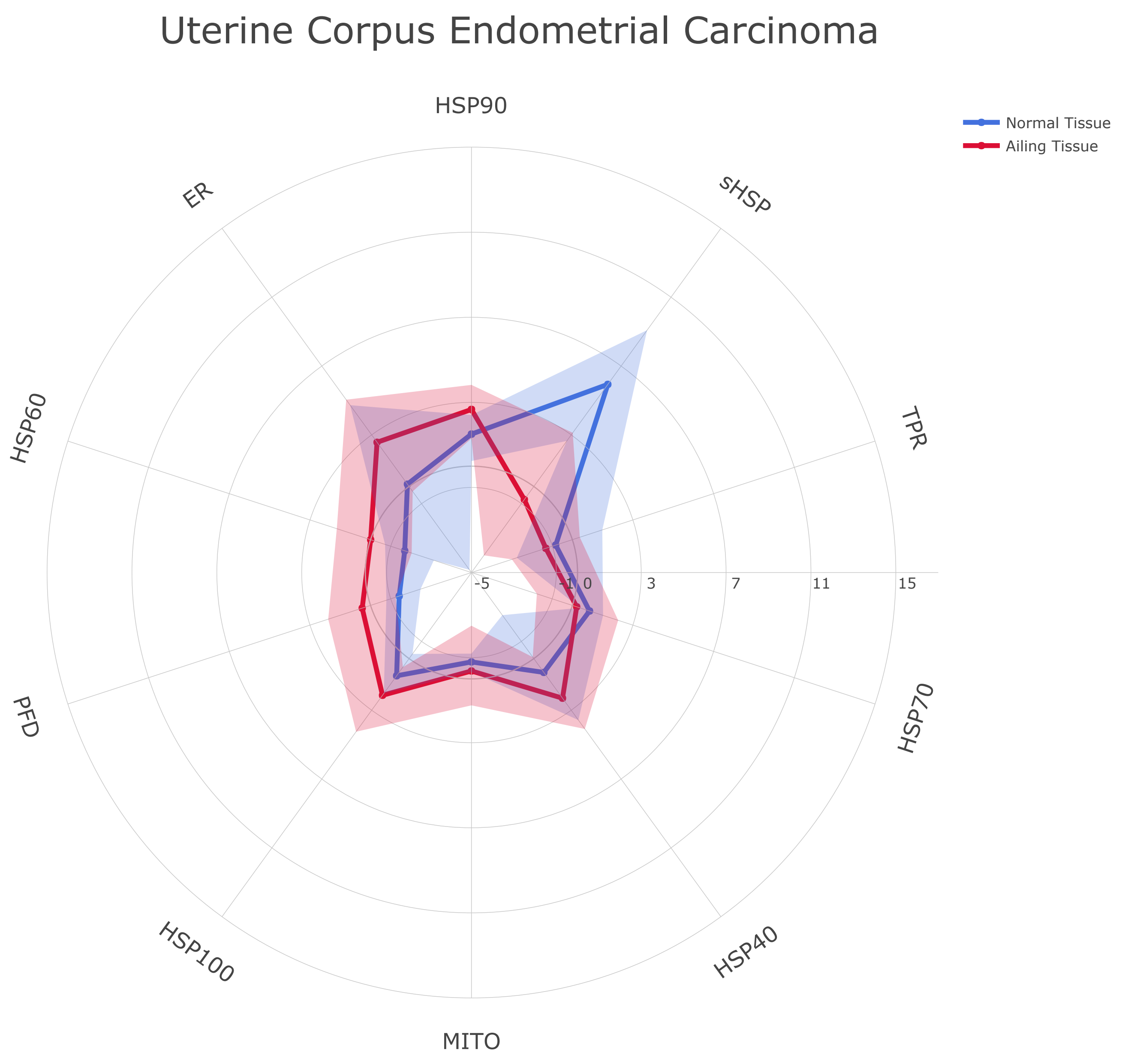
Uterine Corpus Endometrial Carcinoma
HSP90
sHSP
TPR
HSP70
HSP40
MITO
HSP100
PFD
HSP60
ER
-5
-1
0
3
7
11
15
Normal Tissue
Ailing Tissue

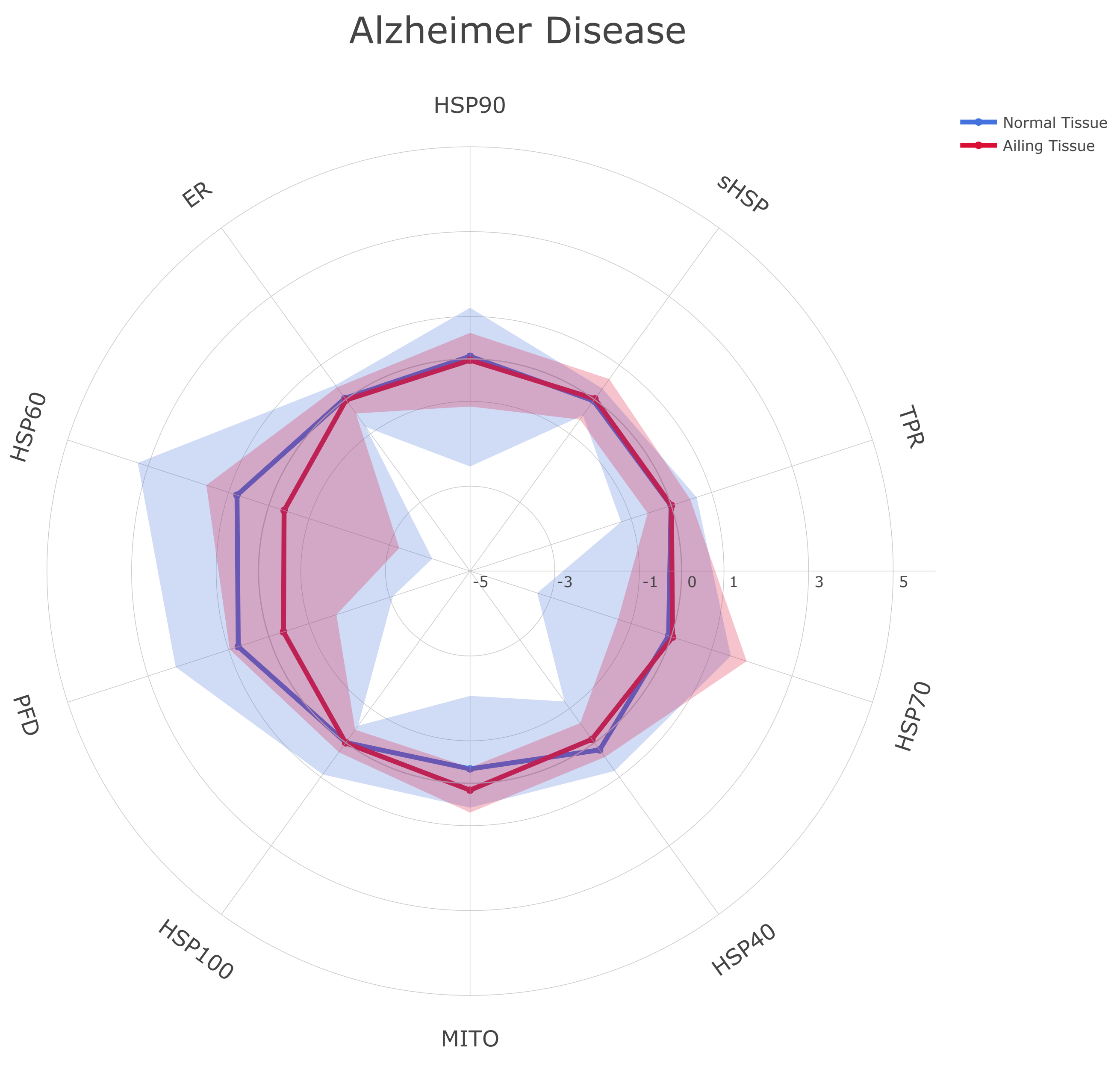
Alzheimer Disease
Normal Tissue
Ailing Tissue
HSP90
sHSP
TPR
HSP70
HSP40
MITO
HSP100
PFD
HSP60
ER
-5
-3
-1
0
1
3
5

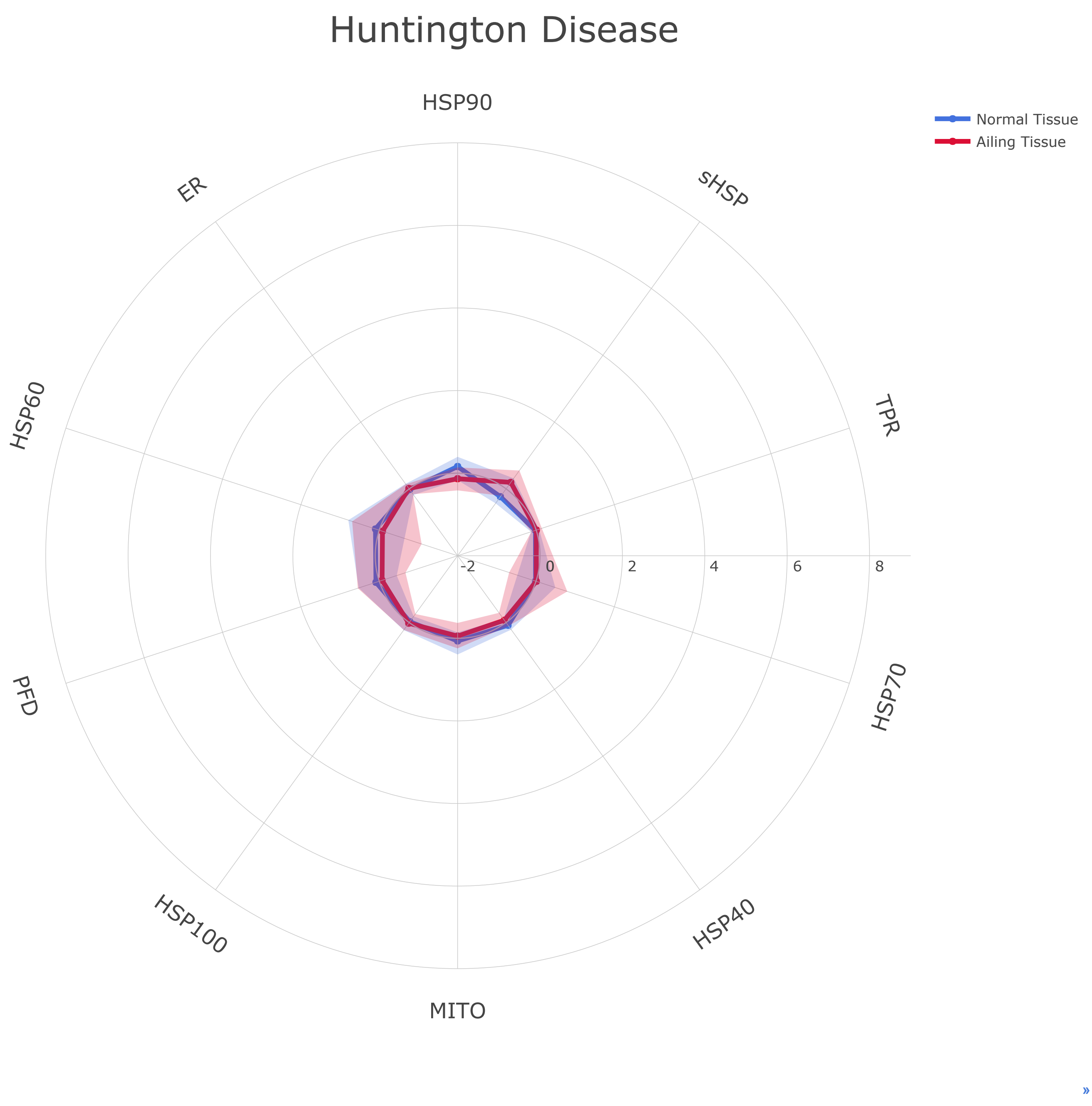
Huntington Disease
Normal Tissue
Ailing Tissue
HSP90
sHSP
TPR
HSP70
HSP40
MITO
HSP100
PFD
HSP60
ER
-2
0
2
4
6
8

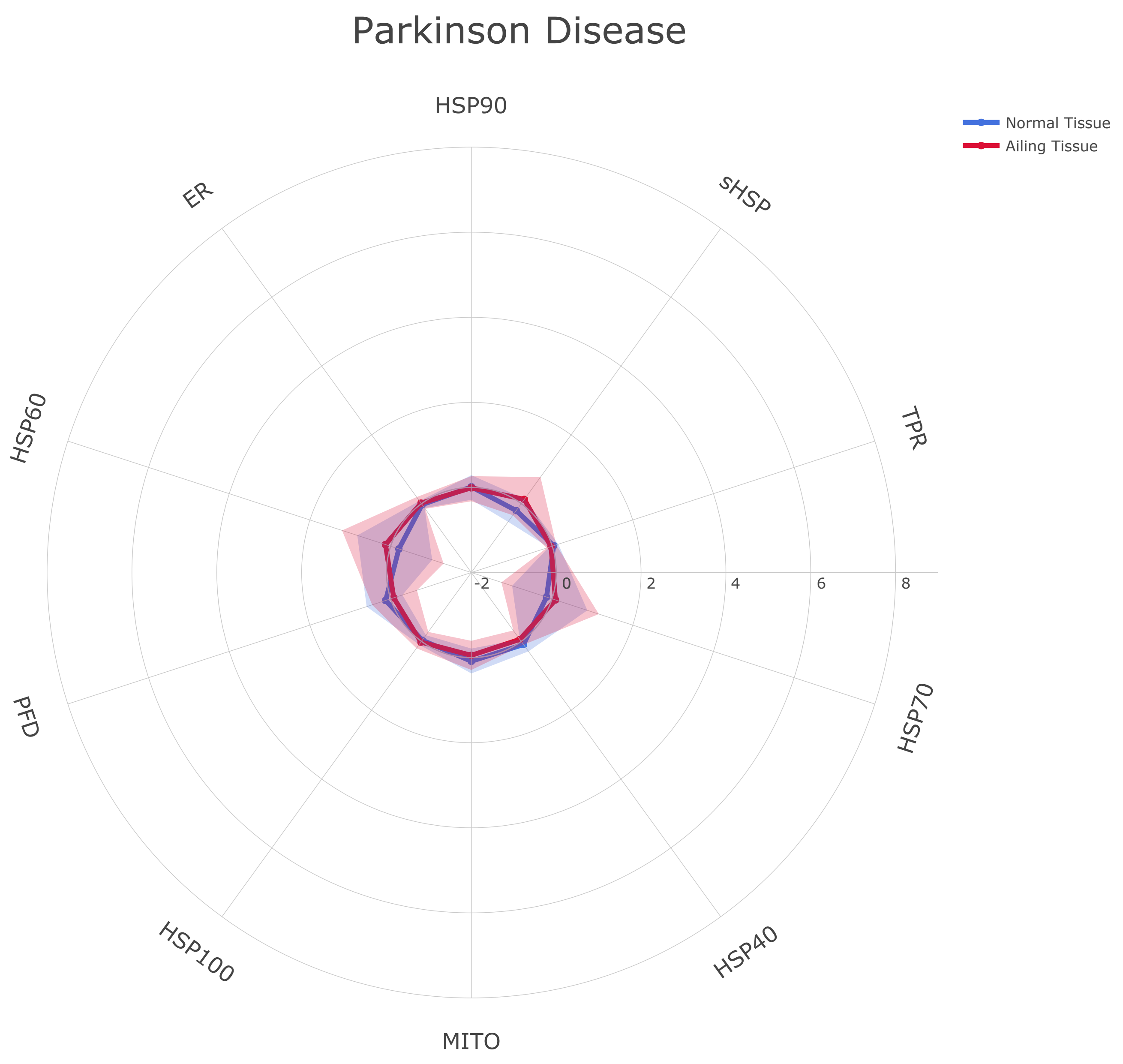

Parkinson Disease
Normal Tissue
Ailing Tissue
HSP90
sHSP
TPR
HSP70
HSP40
MITO
HSP100
PFD
HSP60
ER
-2
0
2
4
6
8

»
