# Supplementary material for: A systematic atlas of chaperome deregulation topologies across the human cancer landscape
Source: PLoS Comput Biol. 2018 Jan 2;14(1):e1005890. doi: 10.1371/journal.pcbi.1005890 (PMC5766242; doi:10.1371/journal.pcbi.1005890)
Supplement: S4 Fig — Chaperome meta-interactome networks for cancers (pp. 1–22) or AD, PD and HD (pp. 23–25) are shown as in Fig 7A. Multiple-evidence chaperome (ME-CHAP) edges and nodes are collapsed onto meta-edges and meta-nodes. Node size and edge thickness correspond to the number of functional family member nodes and the sum of the number of inter-family edges, respectively. Node colour indicates combined disease gene expression changes quantified via Meta-PCA. (PDF) [file pcbi.1005890.s004.pdf]

Figure S4

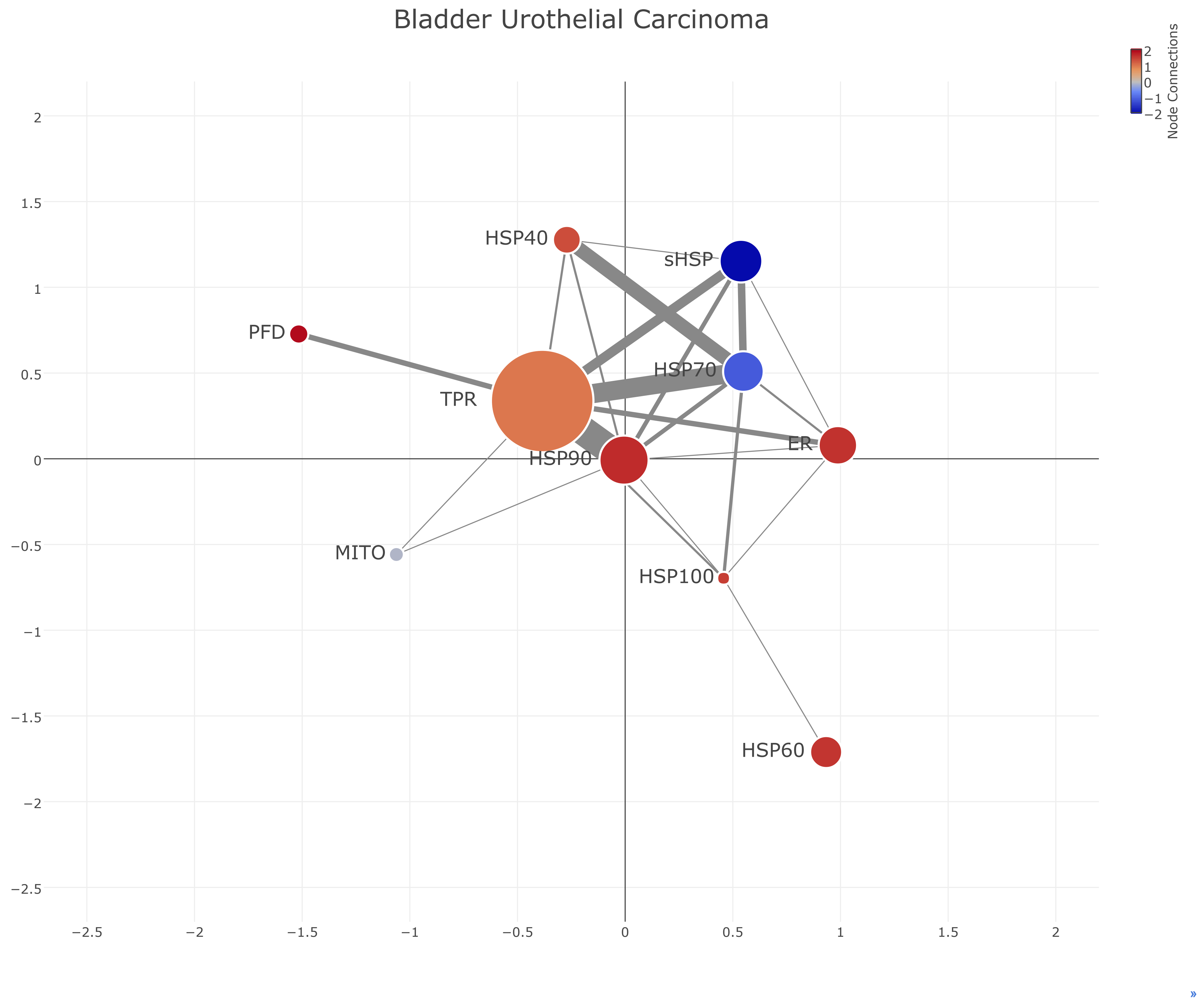

Bladder Urothelial Carcinoma
Node Connections
2
1
0
−1
−2
HSP40
sHSP
PFD
HSP70
TPR
ER
HSP90
MITO
HSP100
HSP60
2
1.5
1
0.5
0
−0.5
−1
−1.5
−2
−2.5
−2.5
−2
−1.5
−1
−0.5
0
0.5
1
1.5
2
»

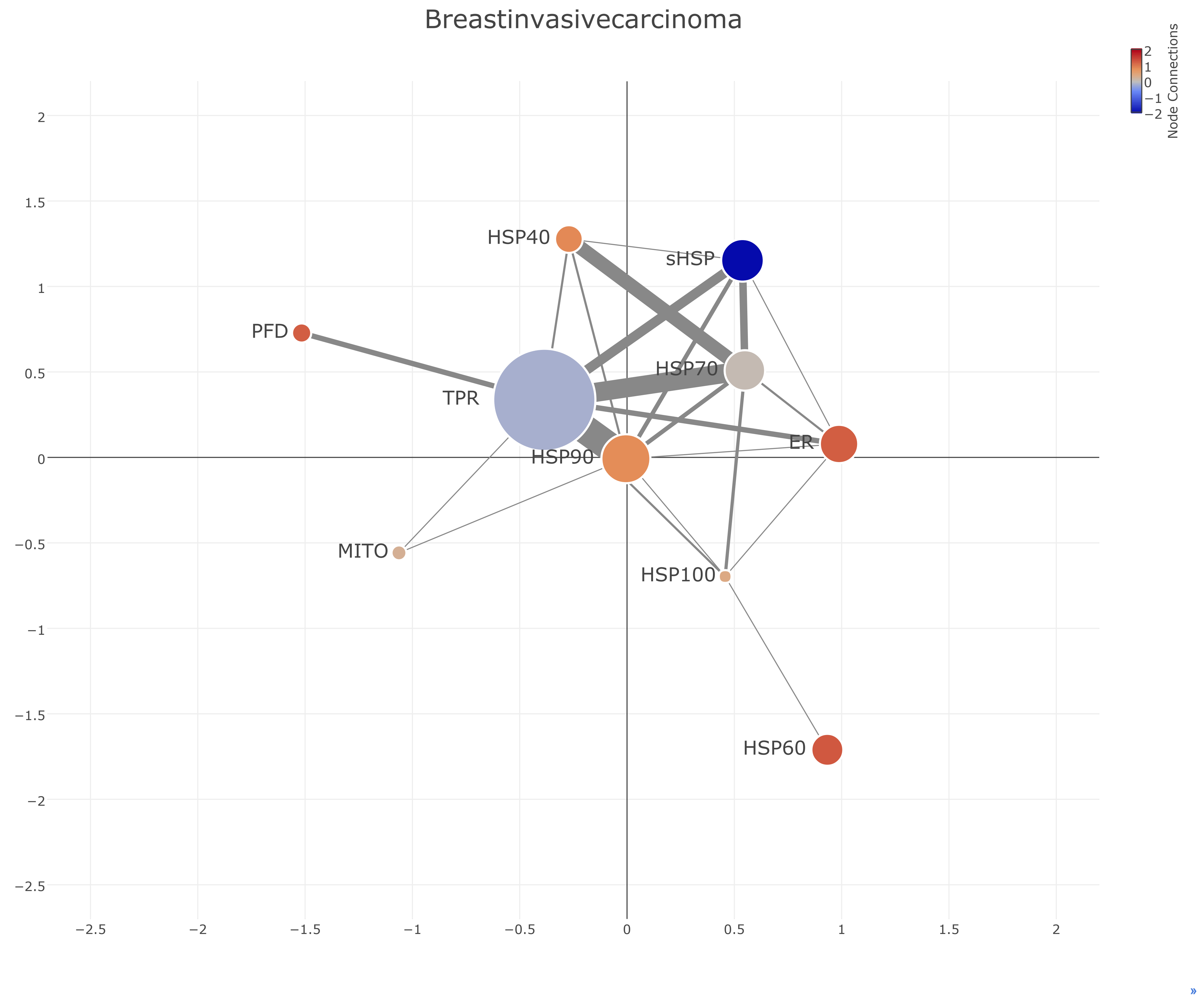
Breastinvasivecarcinoma
Node Connections
2
1
0
−1
−2
HSP40
sHSP
PFD
HSP70
TPR
ER
HSP90
MITO
HSP100
HSP60
2
1.5
1
0.5
0
−0.5
−1
−1.5
−2
−2.5
−2.5
−2
−1.5
−1
−0.5
0
0.5
1
1.5
2
»

Cervicalsquamouscellcarcinomaandendocervicaladenocarcinoma

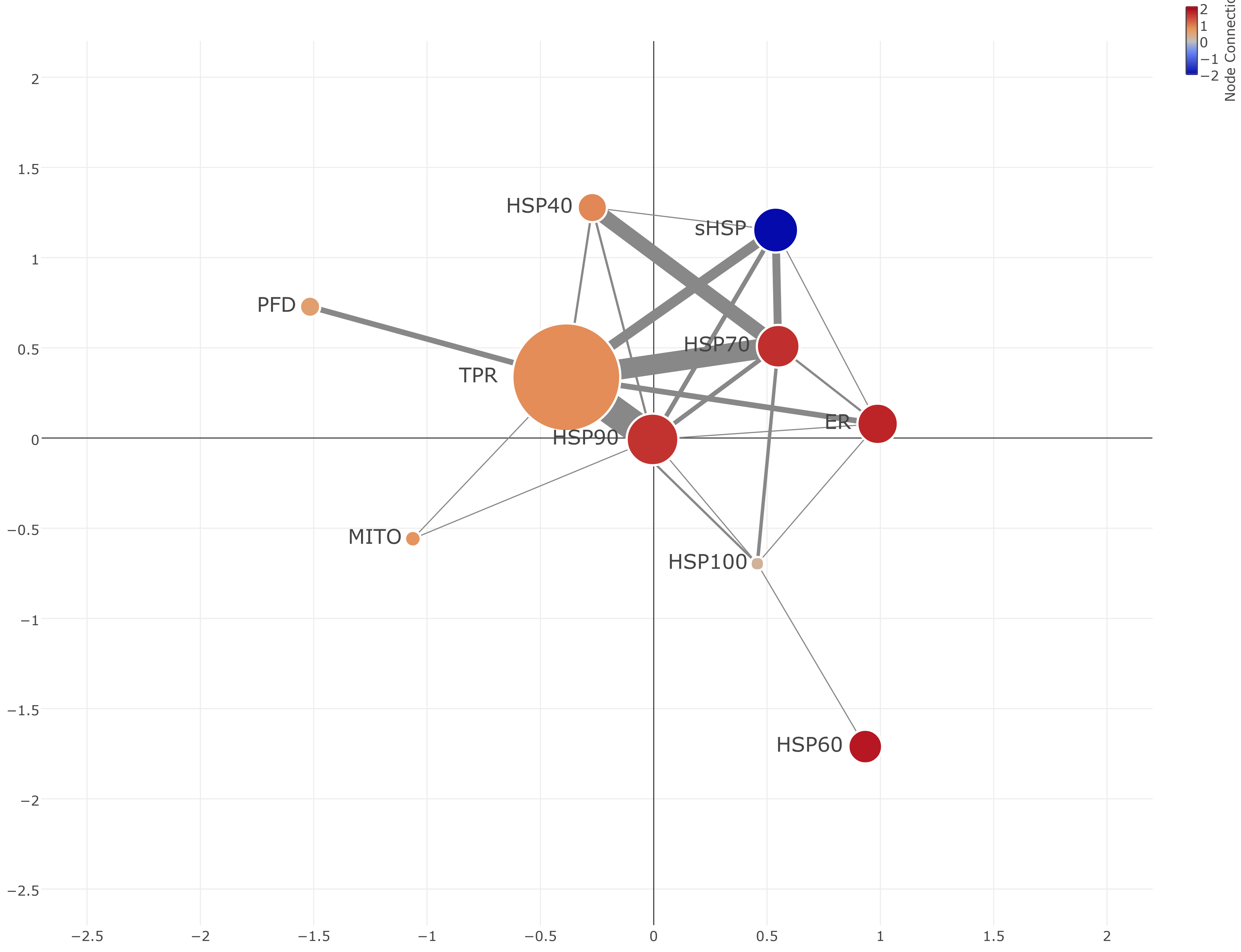

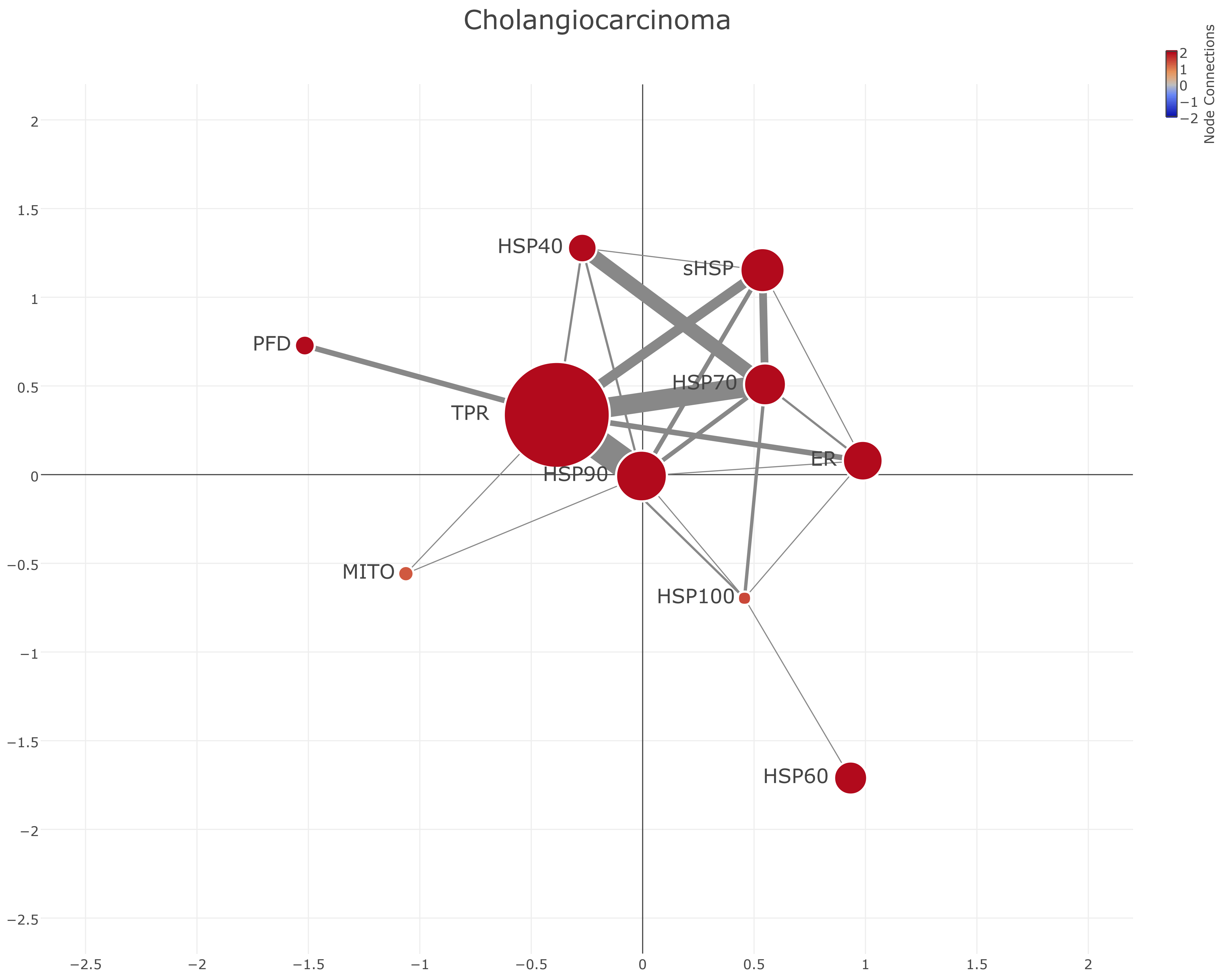
Cholangiocarcinoma
Node Connections
2
1
0
−1
−2
HSP40
sHSP
PFD
HSP70
TPR
HSP90
ER
MITO
HSP100
HSP60
2
1.5
1
0.5
0
−0.5
−1
−1.5
−2
−2.5
−2.5
−2
−1.5
−1
−0.5
0
0.5
1
1.5
2

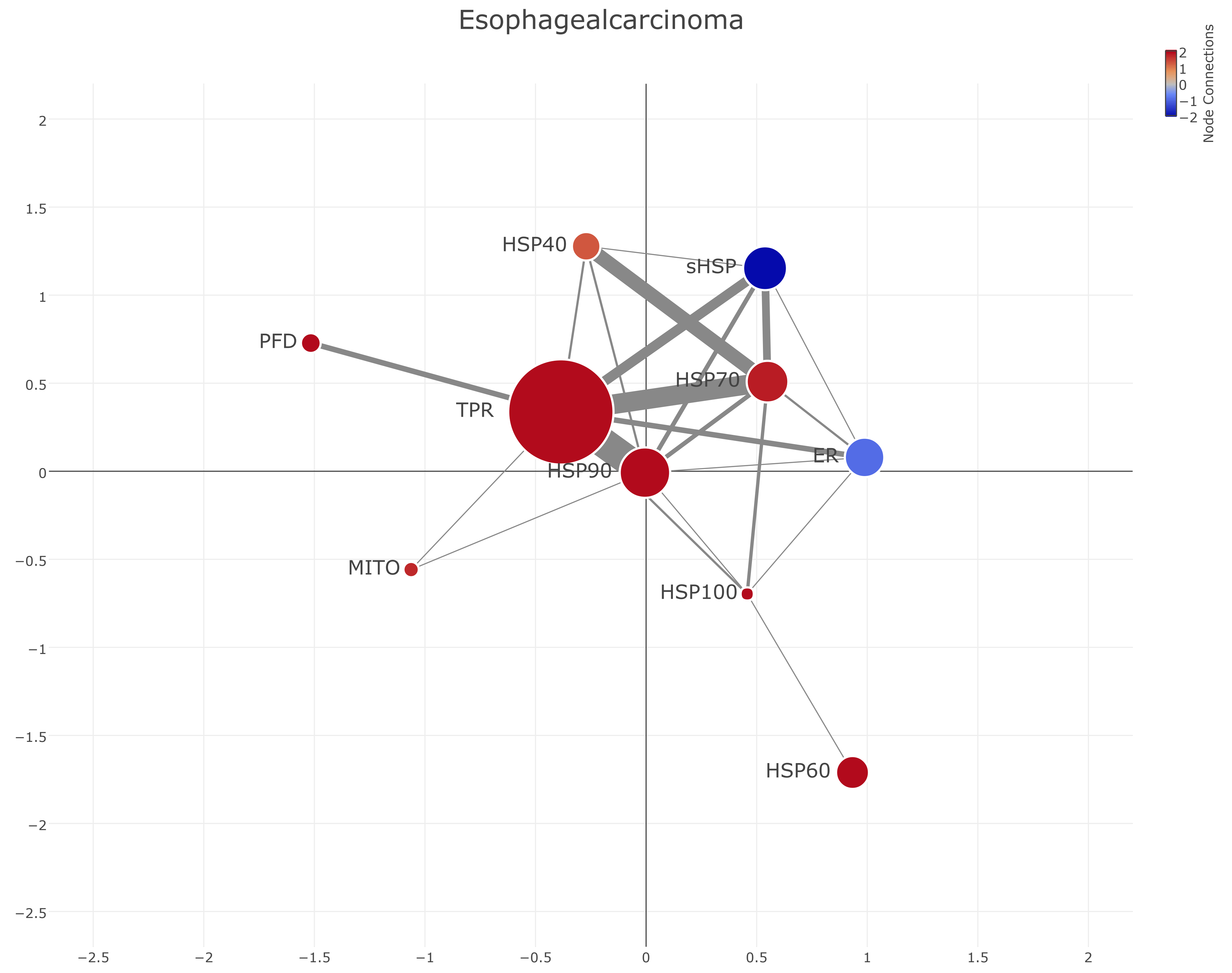

Esophagealcarcinoma
Node Connections
2
1
0
−1
−2
HSP40
sHSP
PFD
HSP70
TPR
ER
HSP90
MITO
HSP100
HSP60
2
1.5
1
0.5
0
−0.5
−1
−1.5
−2
−2.5
−2.5
−2
−1.5
−1
−0.5
0
0.5
1
1.5
2

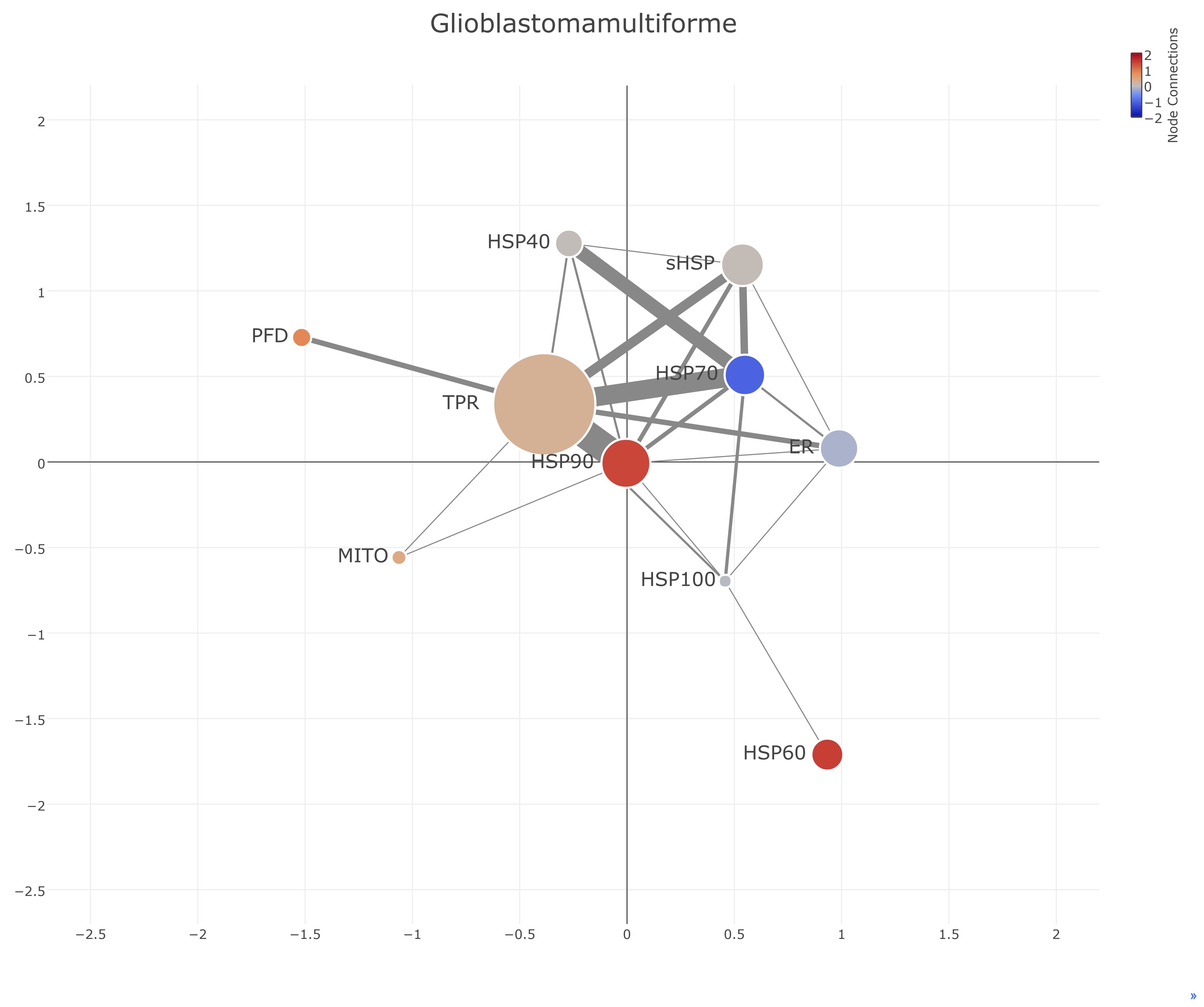
Glioblastomamultiforme
Node Connections
2
1
0
−1
−2
HSP40
sHSP
PFD
HSP70
TPR
ER
HSP90
MITO
HSP100
HSP60
2
1.5
1
0.5
0
−0.5
−1
−1.5
−2
−2.5
−2.5
−2
−1.5
−1
−0.5
0
0.5
1
1.5
2
»

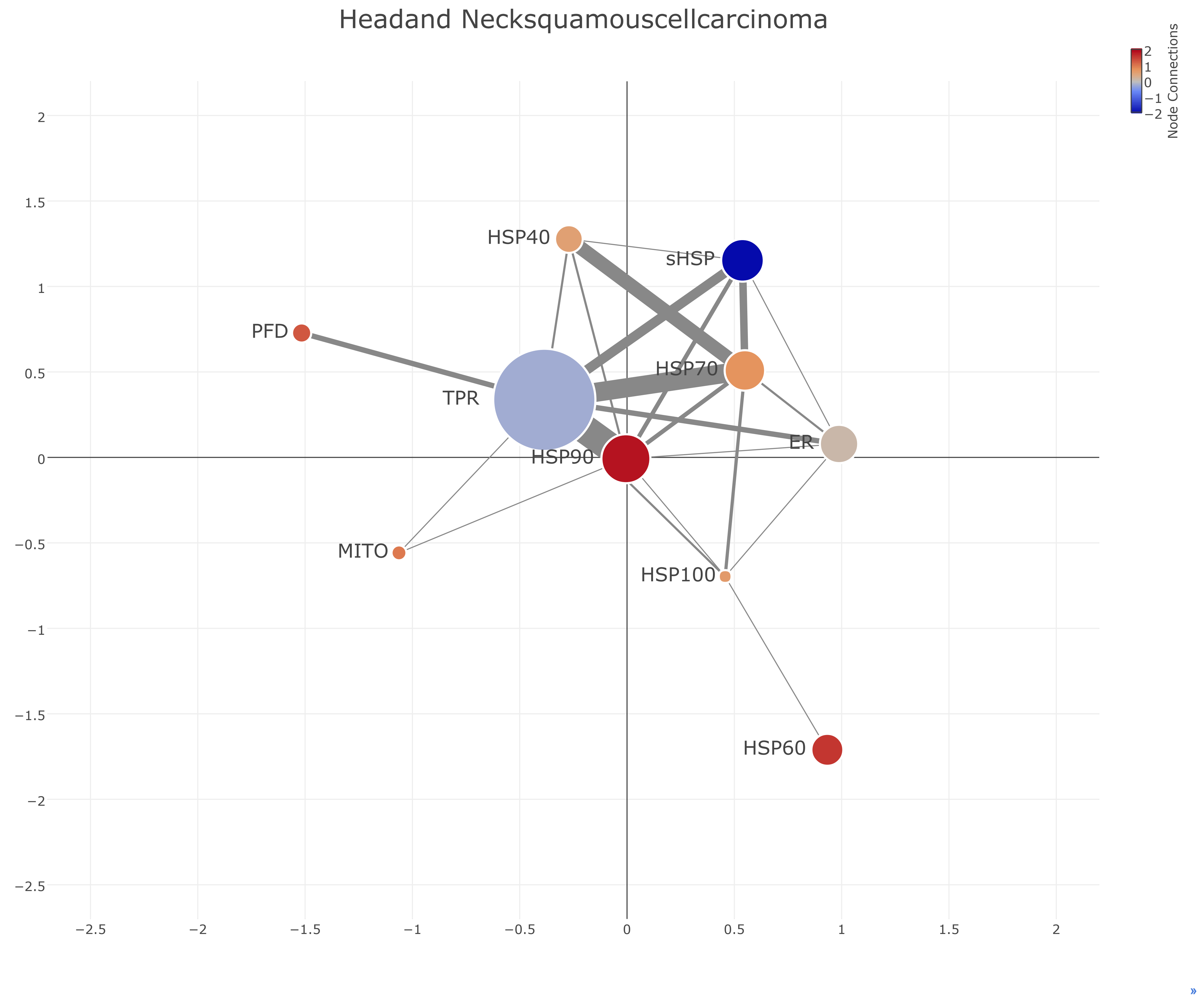
Headand Necksquamouscellcarcinoma
Node Connections
2
1
0
−1
−2
HSP40
sHSP
PFD
HSP70
TPR
ER
HSP90
MITO
HSP100
HSP60
2
1.5
1
0.5
0
−0.5
−1
−1.5
−2
−2.5
−2.5
−2
−1.5
−1
−0.5
0
0.5
1
1.5
2
»

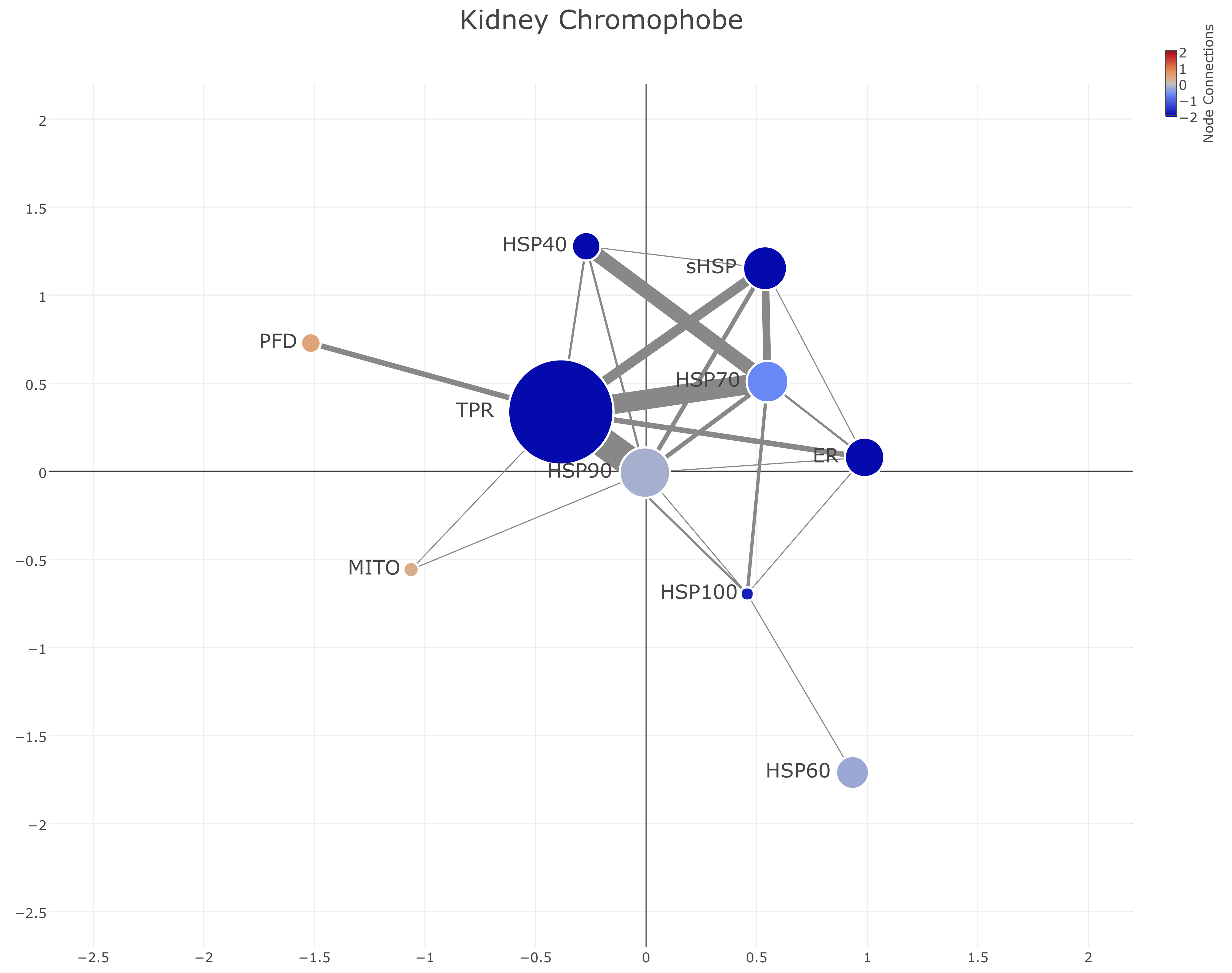
Kidney Chromophobe
Node Connections
2
1
0
−1
−2
HSP40
sHSP
PFD
HSP70
TPR
ER
HSP90
MITO
HSP100
HSP60
2
1.5
1
0.5
0
−0.5
−1
−1.5
−2
−2.5
−2.5
−2
−1.5
−1
−0.5
0
0.5
1
1.5
2

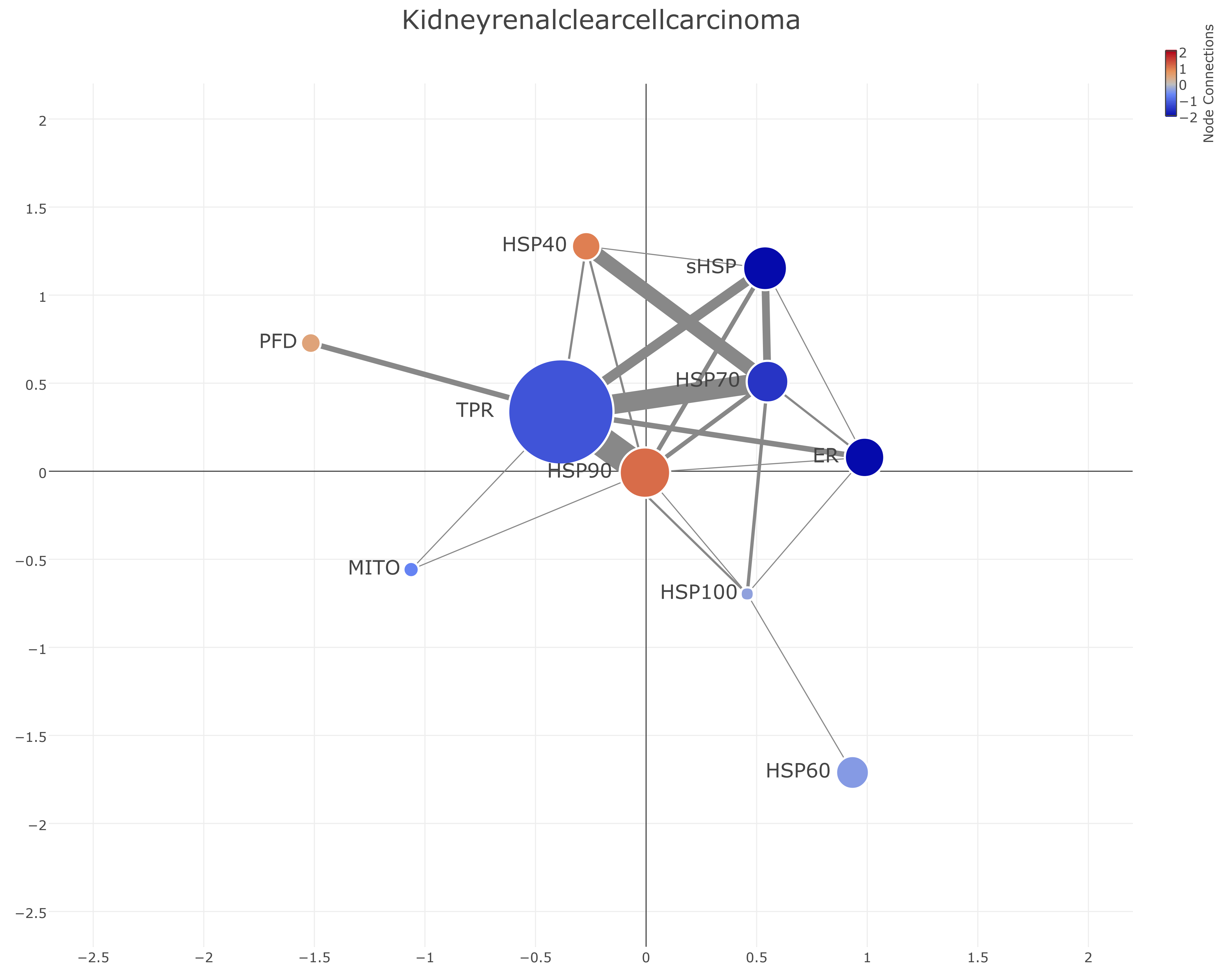
Kidneyrenalclearcellcarcinoma
Node Connections
2
1
0
−1
−2
HSP40
sHSP
PFD
HSP70
TPR
ER
HSP90
MITO
HSP100
HSP60
2
1.5
1
0.5
0
−0.5
−1
−1.5
−2
−2.5
−2.5
−2
−1.5
−1
−0.5
0
0.5
1
1.5
2

»

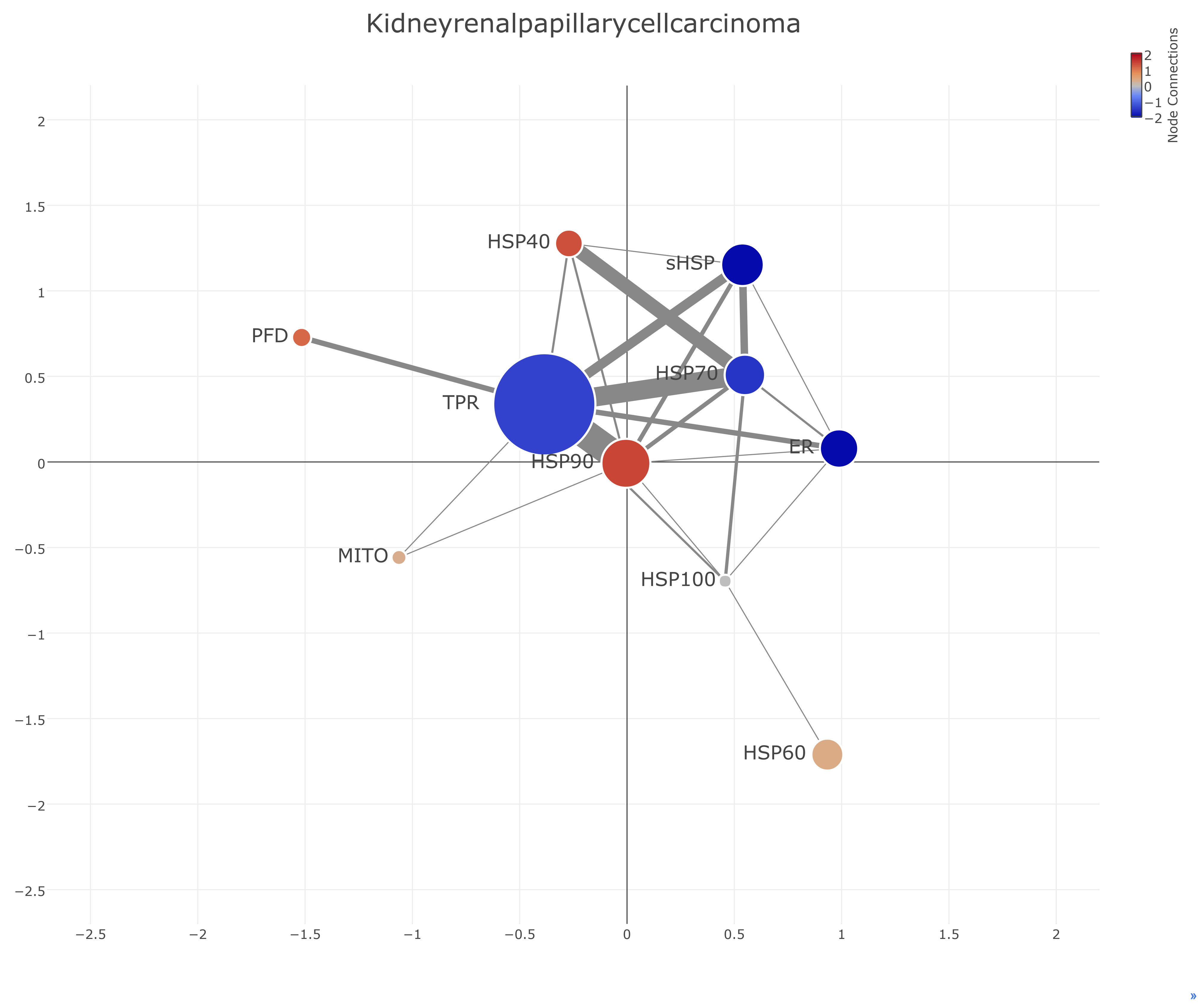
Kidneyrenalpapillarycellcarcinoma
Node Connections
2
1
0
−1
−2
HSP40
sHSP
PFD
HSP70
TPR
ER
HSP90
MITO
HSP100
HSP60
2
1.5
1
0.5
0
−0.5
−1
−1.5
−2
−2.5
−2.5
−2
−1.5
−1
−0.5
0
0.5
1
1.5
2

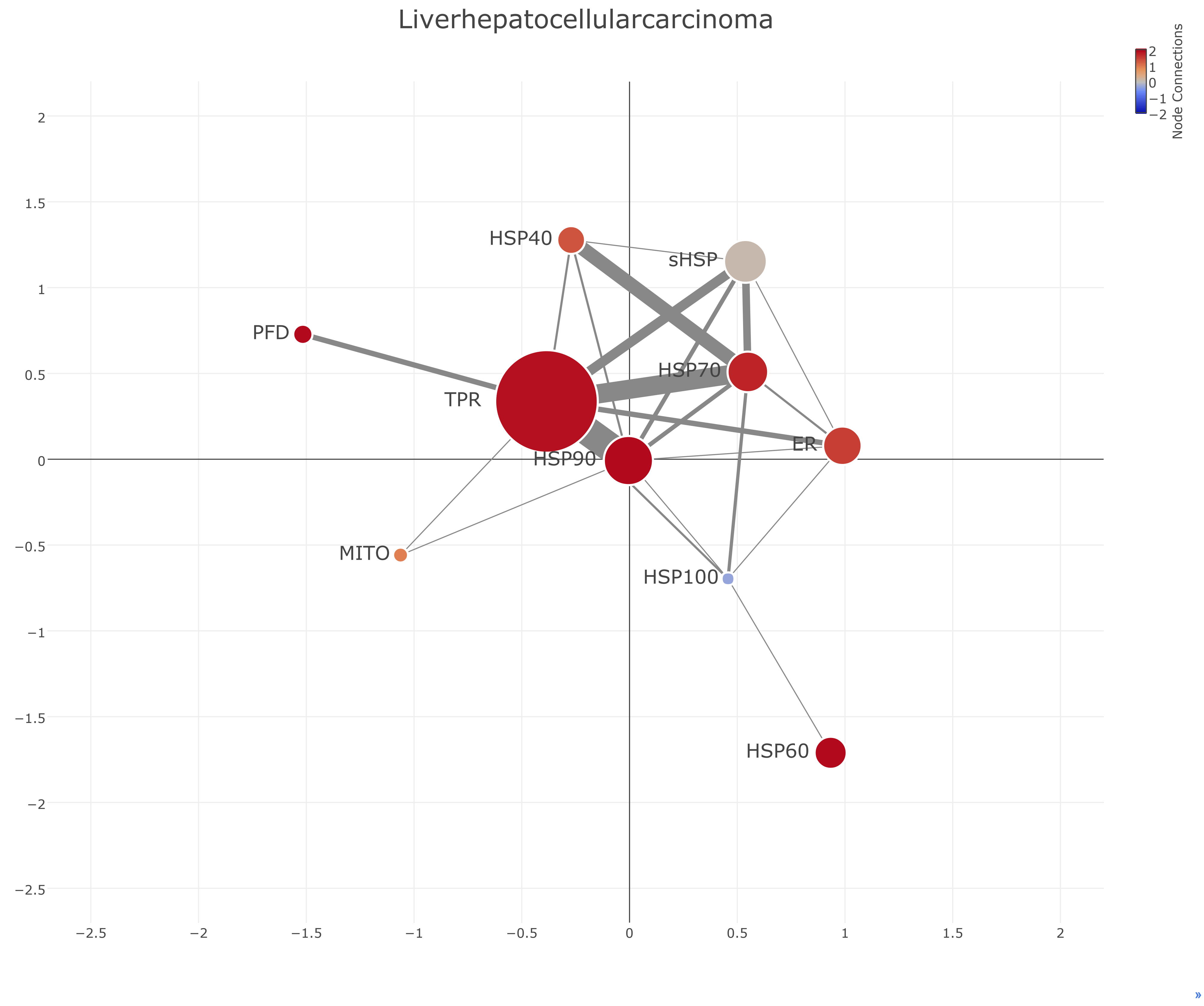

Liverhepatocellularcarcinoma
Node Connections
2
1
0
−1
−2
HSP40
sHSP
PFD
HSP70
TPR
HSP90
ER
MITO
HSP100
HSP60
2
1.5
1
0.5
0
−0.5
−1
−1.5
−2
−2.5
−2.5
−2
−1.5
−1
−0.5
0
0.5
1
1.5
2

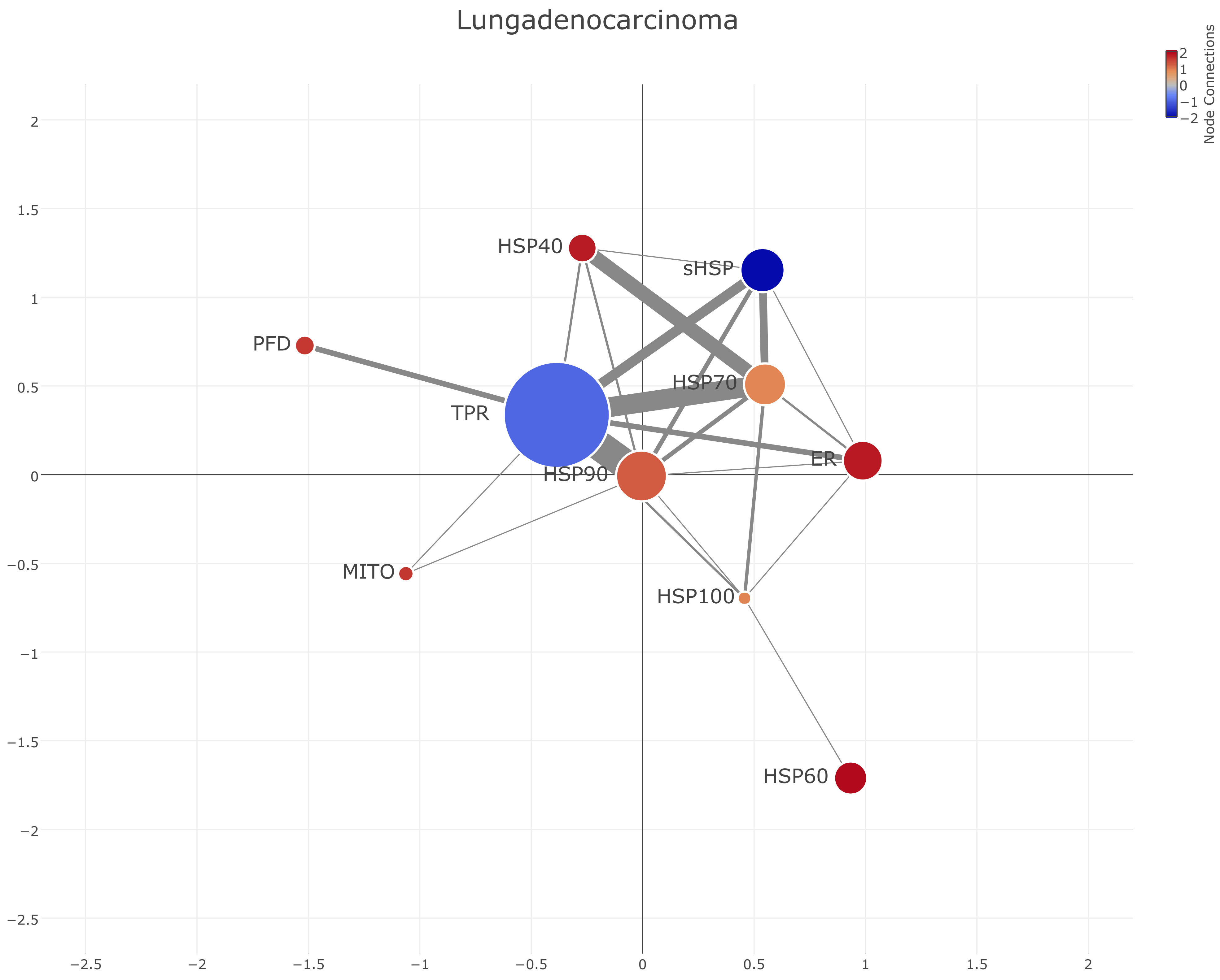

Lungadenocarcinoma
Node Connections
2
1
0
−1
−2
HSP40
sHSP
PFD
HSP70
TPR
ER
HSP90
MITO
HSP100
HSP60
2
1.5
1
0.5
0
−0.5
−1
−1.5
−2
−2.5
−2.5
−2
−1.5
−1
−0.5
0
0.5
1
1.5
2

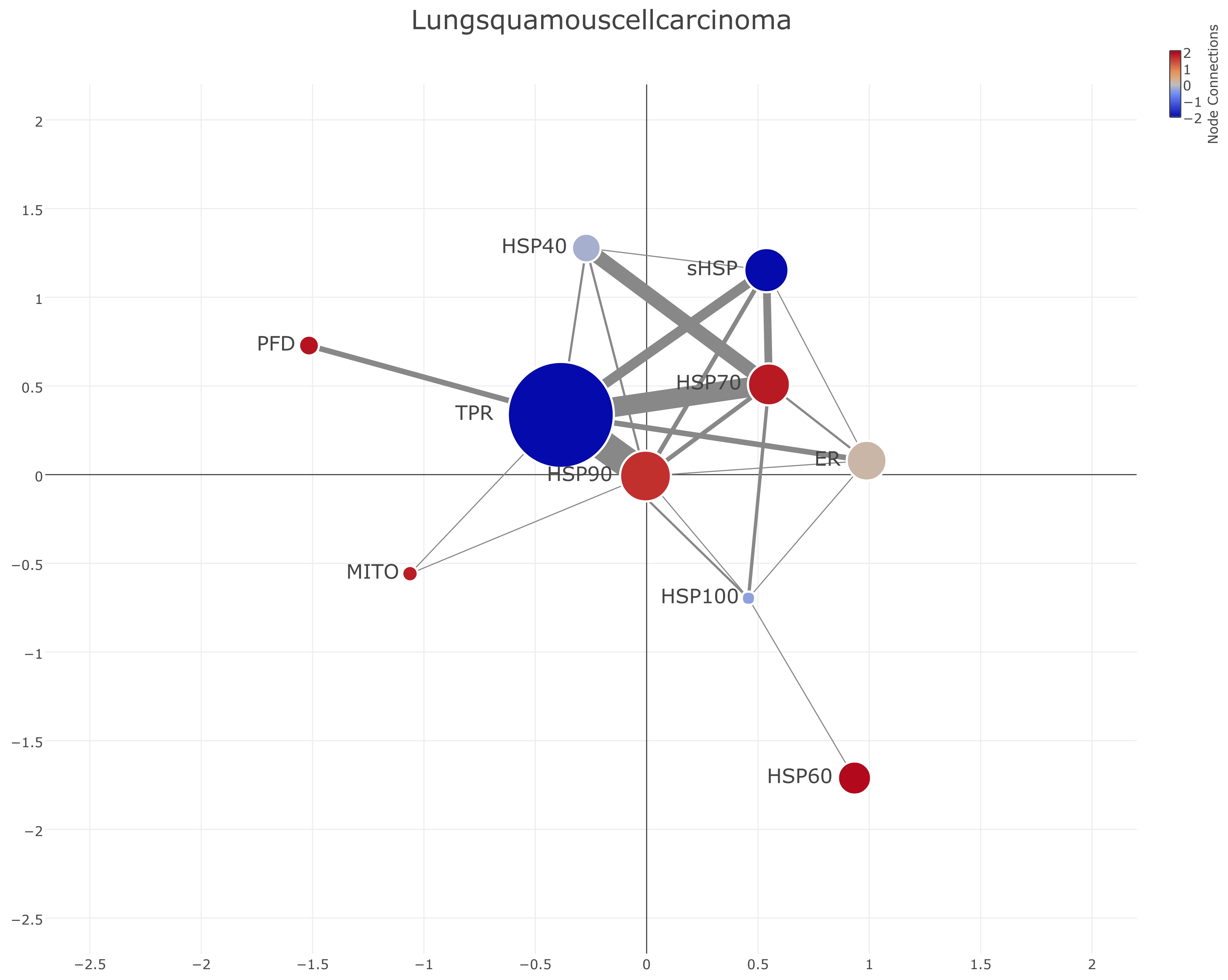

Lungsquamouscellcarcinoma
Node Connections
2
1
0
−1
−2
HSP40
sHSP
PFD
HSP70
TPR
ER
HSP90
MITO
HSP100
HSP60
2
1.5
1
0.5
0
−0.5
−1
−1.5
−2
−2.5
−2.5
−2
−1.5
−1
−0.5
0
0.5
1
1.5
2

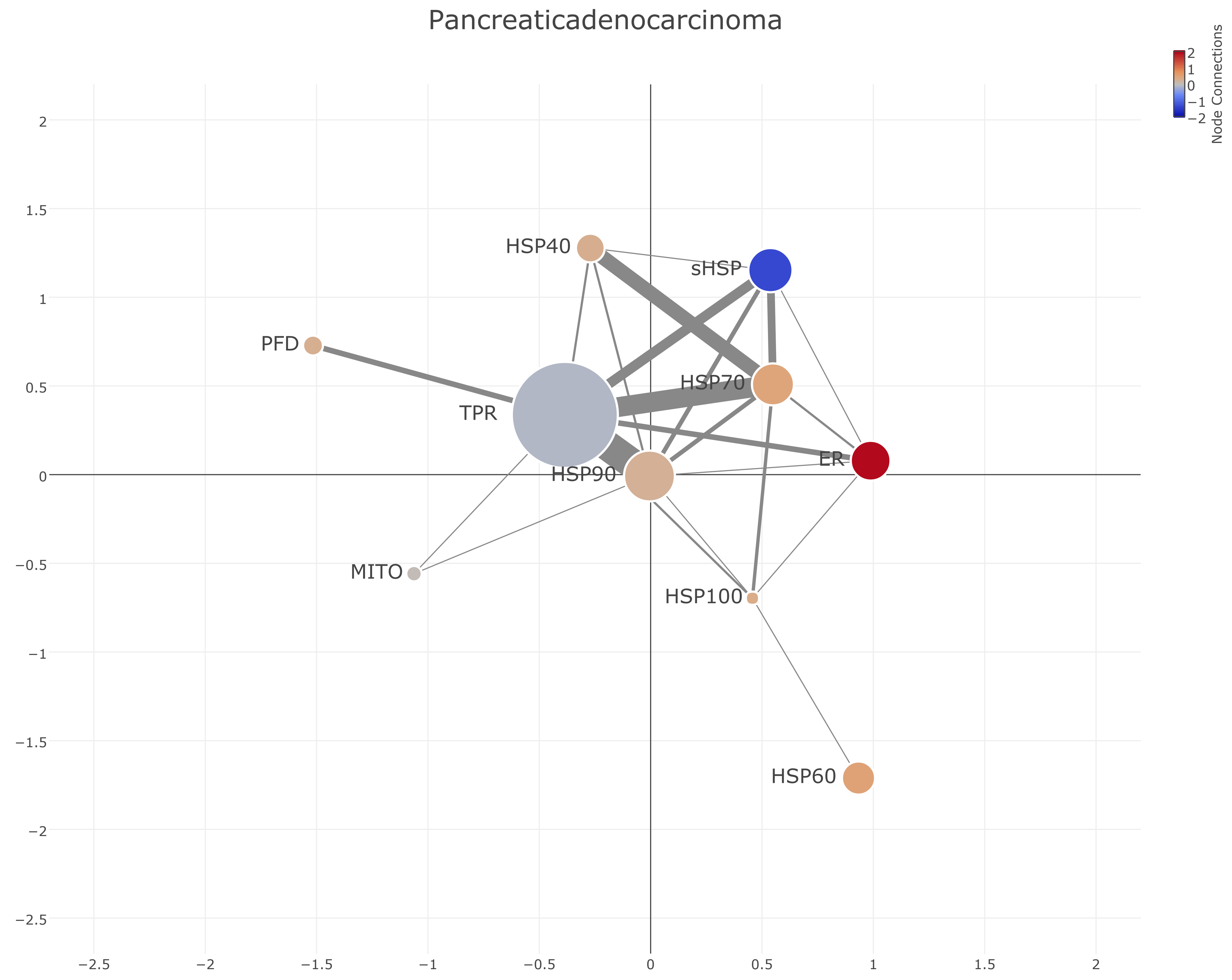
Pancreaticadenocarcinoma
Node Connections
2
1
0
−1
−2
HSP40
sHSP
PFD
HSP70
TPR
ER
HSP90
MITO
HSP100
HSP60
2
1.5
1
0.5
0
−0.5
−1
−1.5
−2
−2.5
−2.5
−2
−1.5
−1
−0.5
0
0.5
1
1.5
2

»

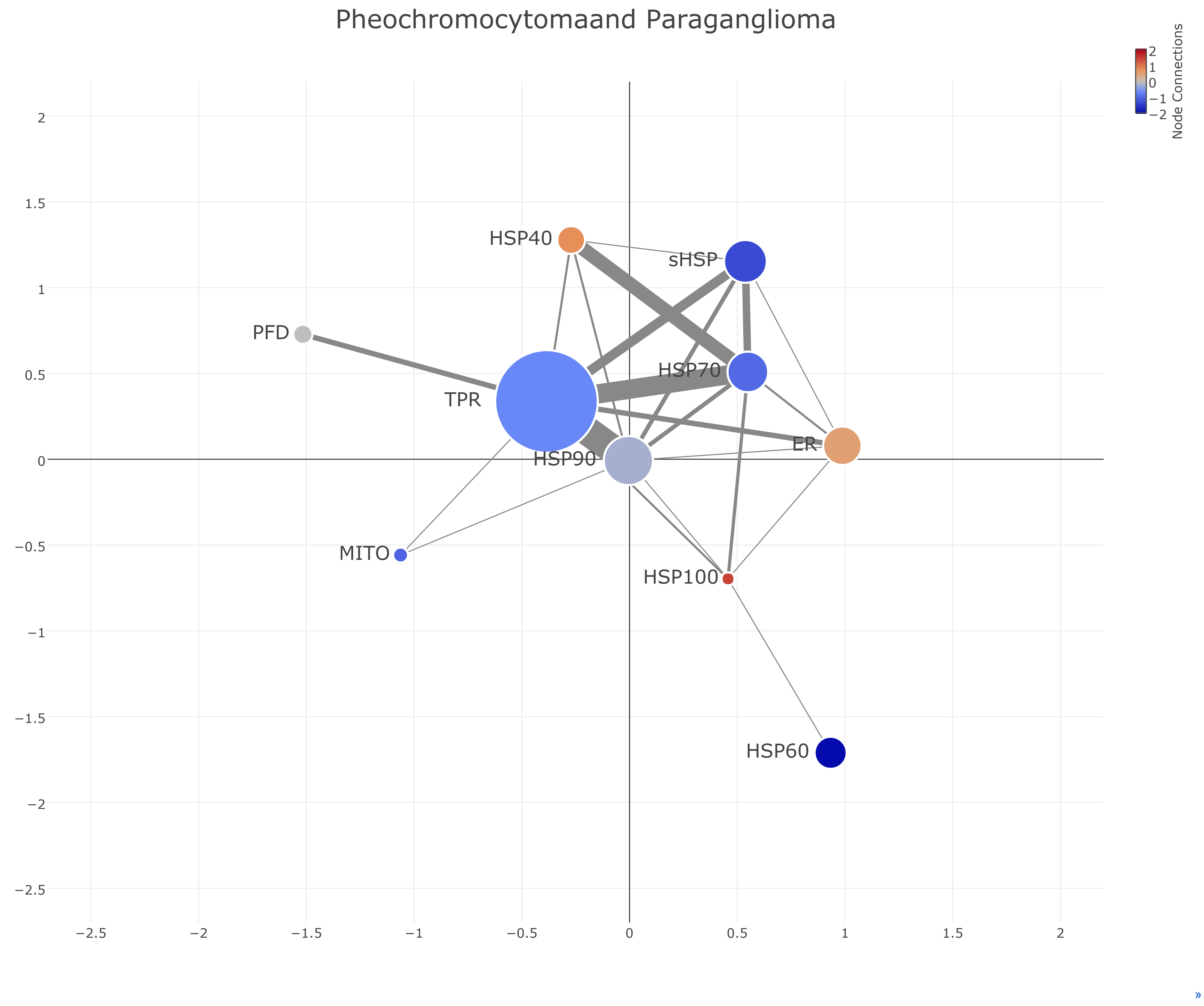
Pheochromocytomaand Paraganglioma
Node Connections
2
1
0
−1
−2
HSP40
sHSP
PFD
HSP70
TPR
ER
HSP90
MITO
HSP100
HSP60
2
1.5
1
0.5
0
−0.5
−1
−1.5
−2
−2.5
−2.5
−2
−1.5
−1
−0.5
0
0.5
1
1.5
2

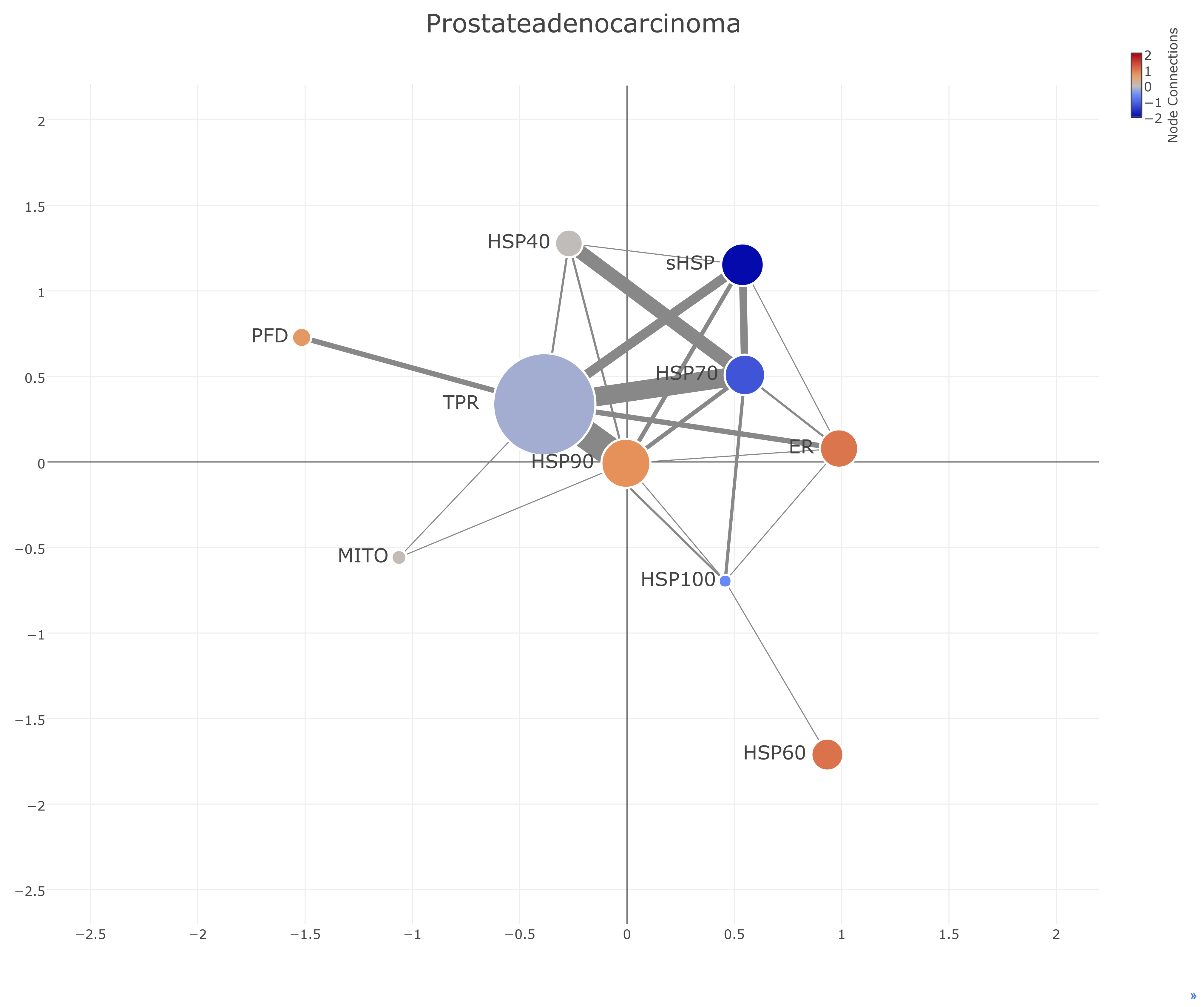

Prostateadenocarcinoma
Node Connections
2
1
0
−1
−2
HSP40
sHSP
PFD
HSP70
TPR
ER
HSP90
MITO
HSP100
HSP60
2
1.5
1
0.5
0
−0.5
−1
−1.5
−2
−2.5
−2.5
−2
−1.5
−1
−0.5
0
0.5
1
1.5
2
»

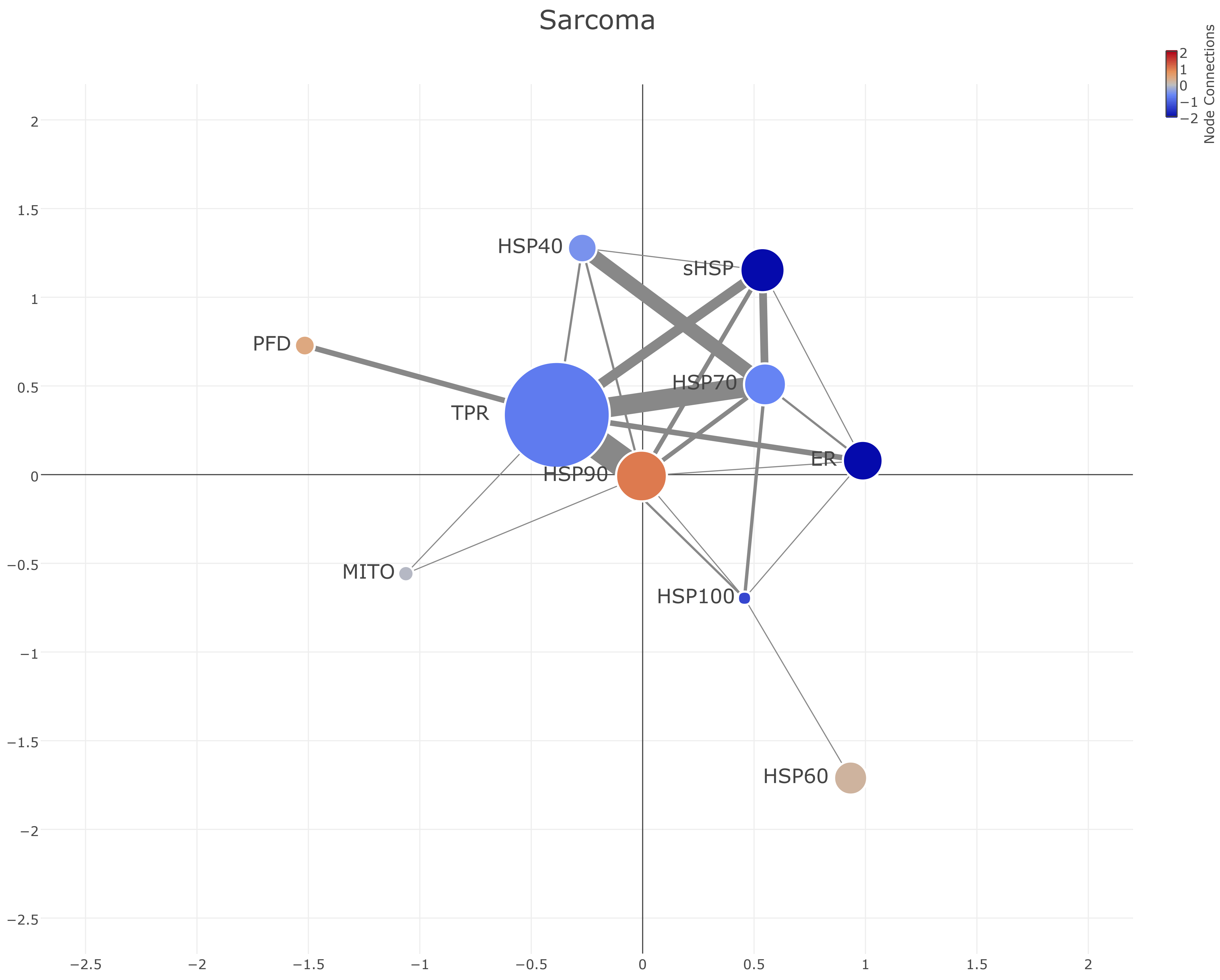
Sarcoma
Node Connections
2
1
0
−1
−2
HSP40
sHSP
PFD
HSP70
TPR
ER
HSP90
MITO
HSP100
HSP60
2
1.5
1
0.5
0
−0.5
−1
−1.5
−2
−2.5
−2.5
−2
−1.5
−1
−0.5
0
0.5
1
1.5
2

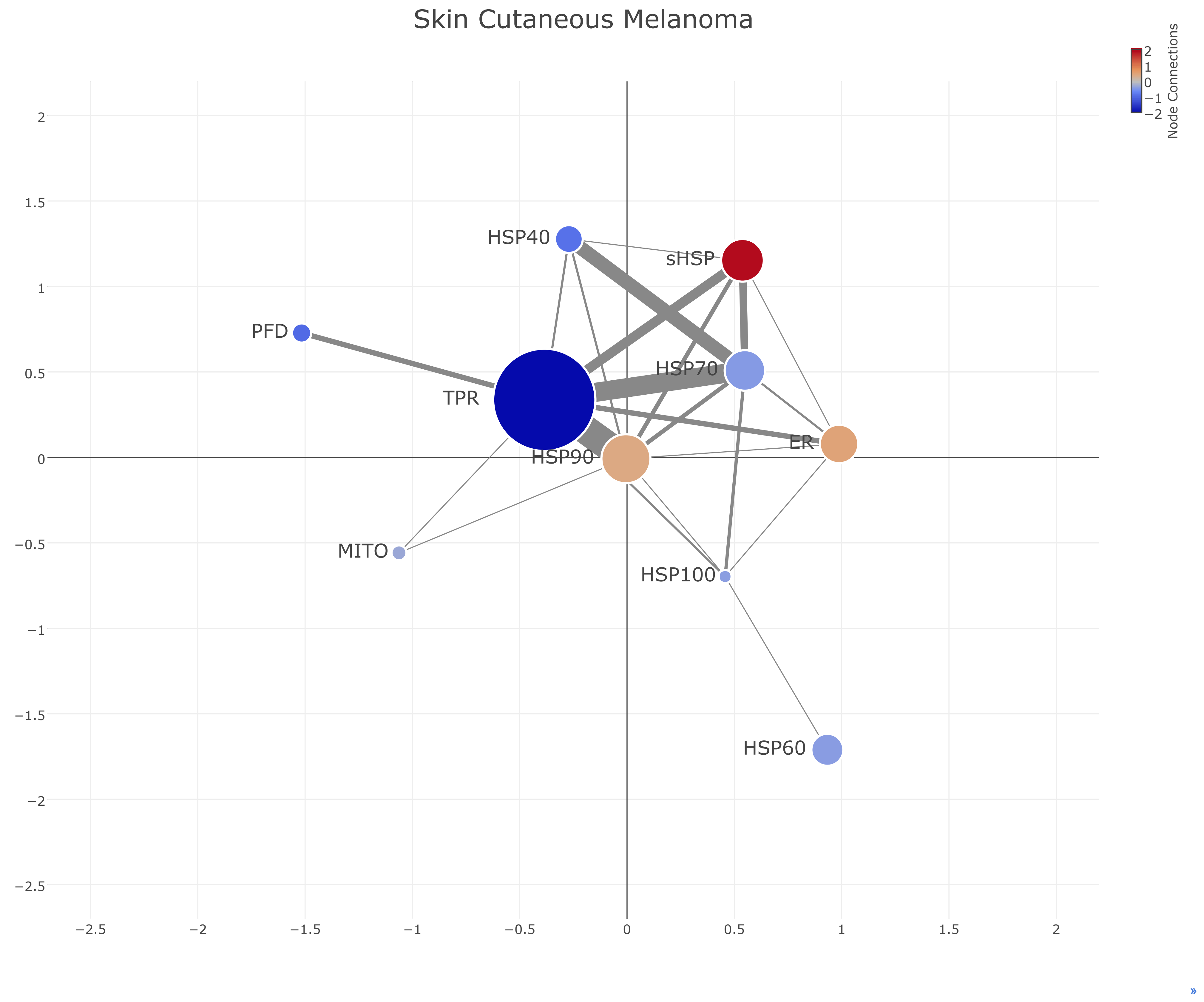
Skin Cutaneous Melanoma
Node Connections
2
1
0
−1
−2
HSP40
sHSP
PFD
HSP70
TPR
ER
HSP90
MITO
HSP100
HSP60
2
1.5
1
0.5
0
−0.5
−1
−1.5
−2
−2.5
−2.5
−2
−1.5
−1
−0.5
0
0.5
1
1.5
2
»

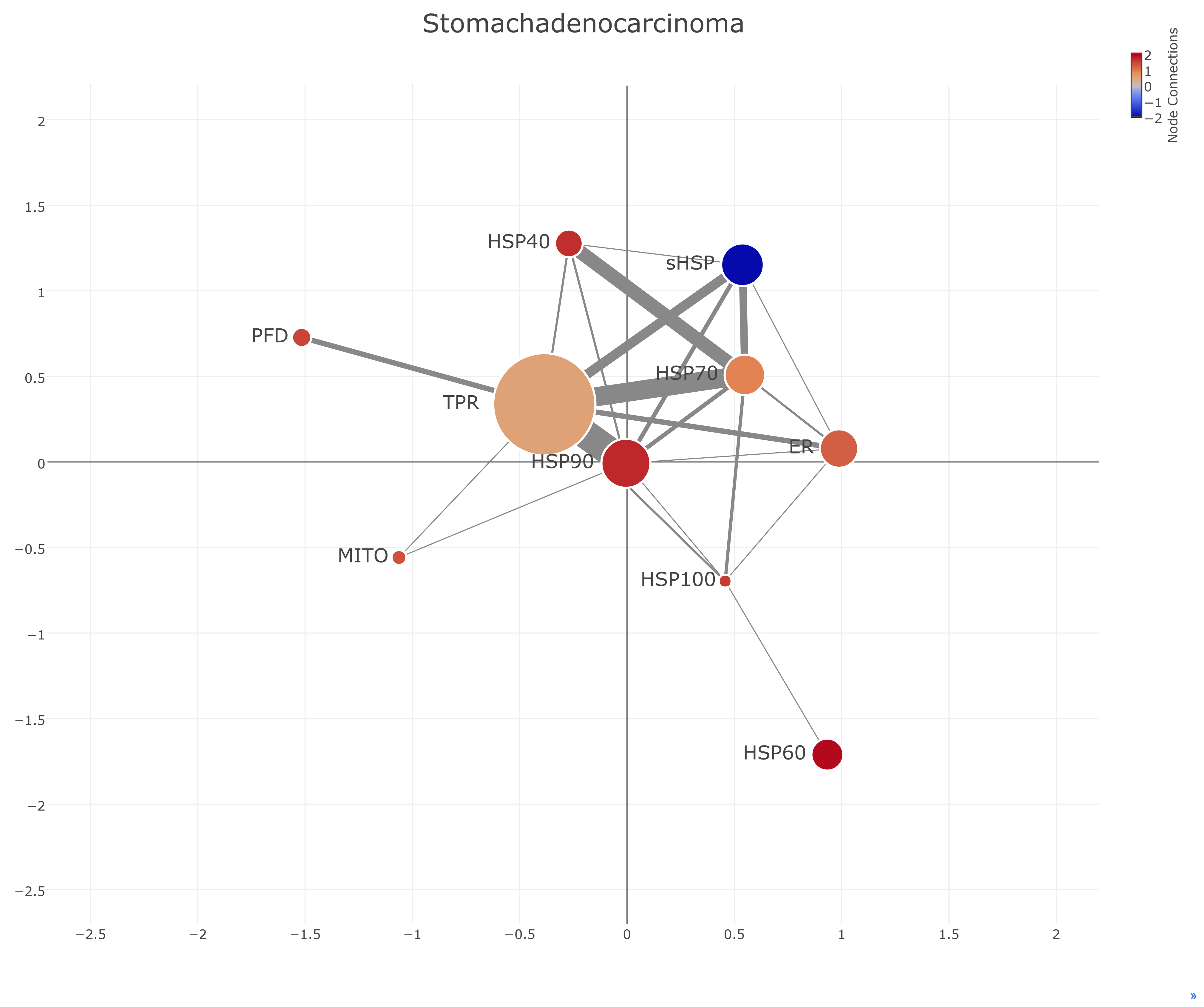
Stomachadenocarcinoma
Node Connections
2
1
0
−1
−2
HSP40
sHSP
PFD
HSP70
TPR
ER
HSP90
MITO
HSP100
HSP60
2
1.5
1
0.5
0
−0.5
−1
−1.5
−2
−2.5
−2.5
−2
−1.5
−1
−0.5
0
0.5
1
1.5
2

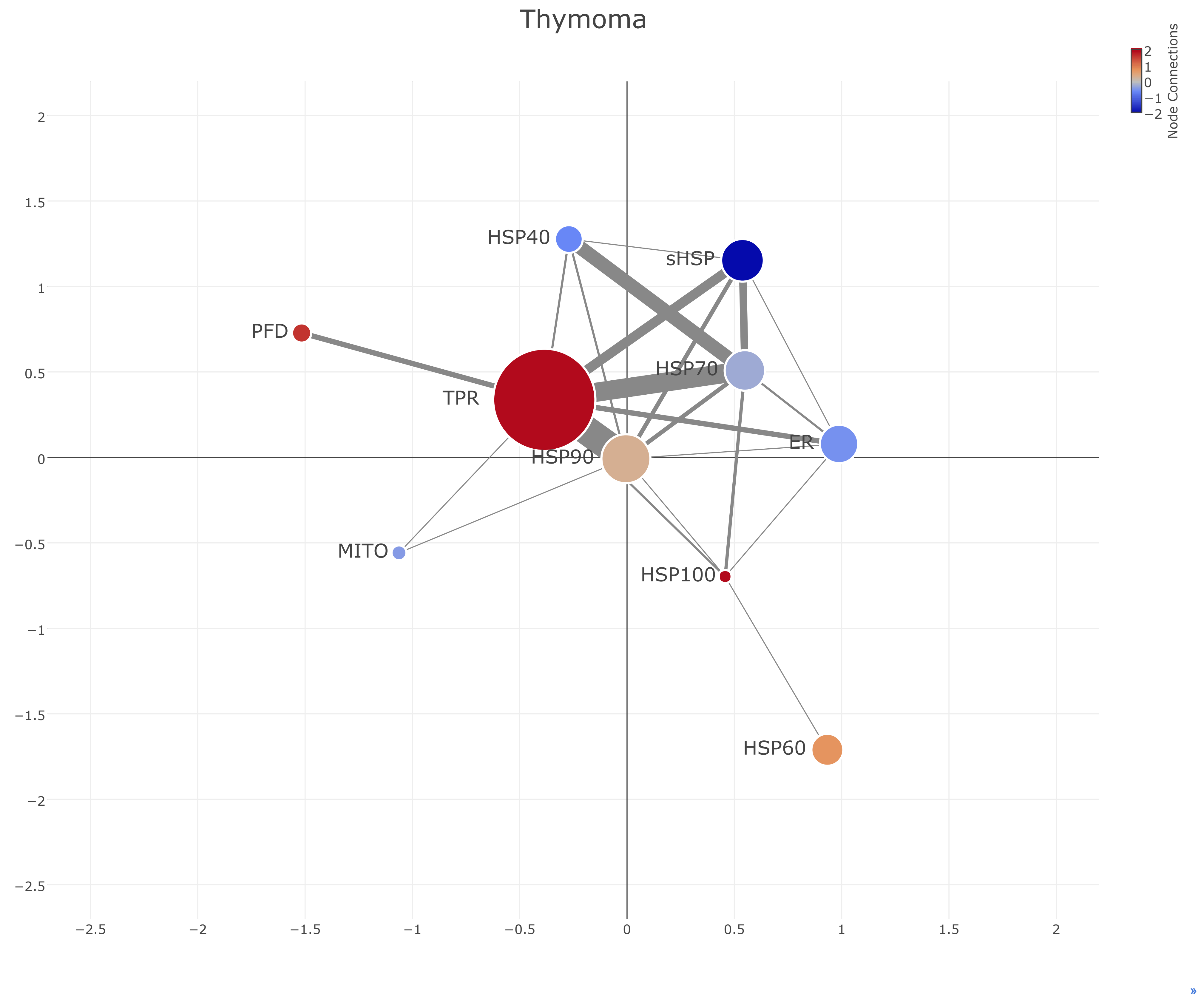
Thymoma
Node Connections
2
1
0
−1
−2
HSP40
sHSP
PFD
HSP70
TPR
ER
HSP90
MITO
HSP100
HSP60
2
1.5
1
0.5
0
−0.5
−1
−1.5
−2
−2.5
−2.5
−2
−1.5
−1
−0.5
0
0.5
1
1.5
2
»

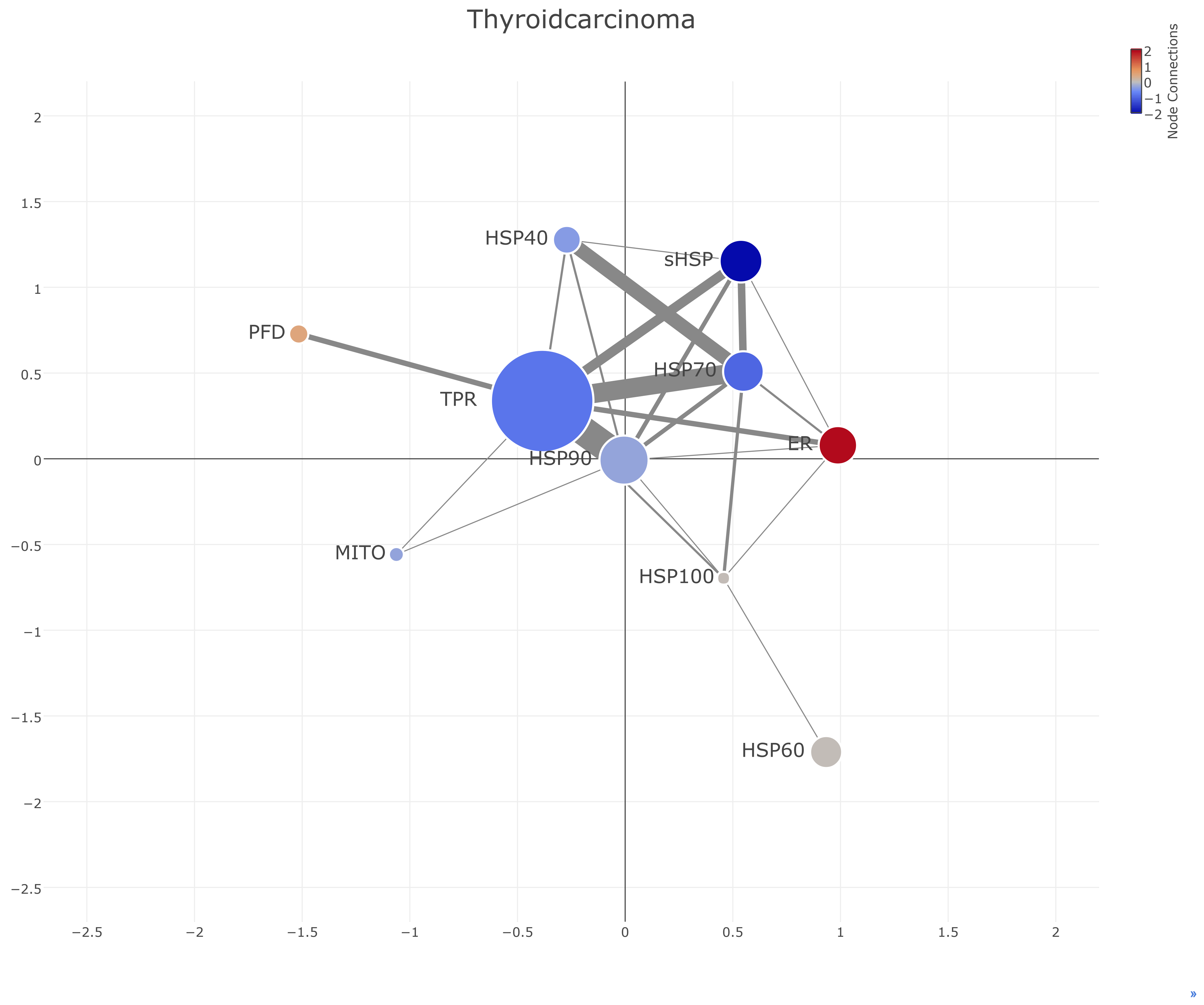
Thyroidcarcinoma
Node Connections
2
1
0
−1
−2
HSP40
sHSP
PFD
HSP70
TPR
ER
HSP90
MITO
HSP100
HSP60
2
1.5
1
0.5
0
−0.5
−1
−1.5
−2
−2.5
−2.5
−2
−1.5
−1
−0.5
0
0.5
1
1.5
2

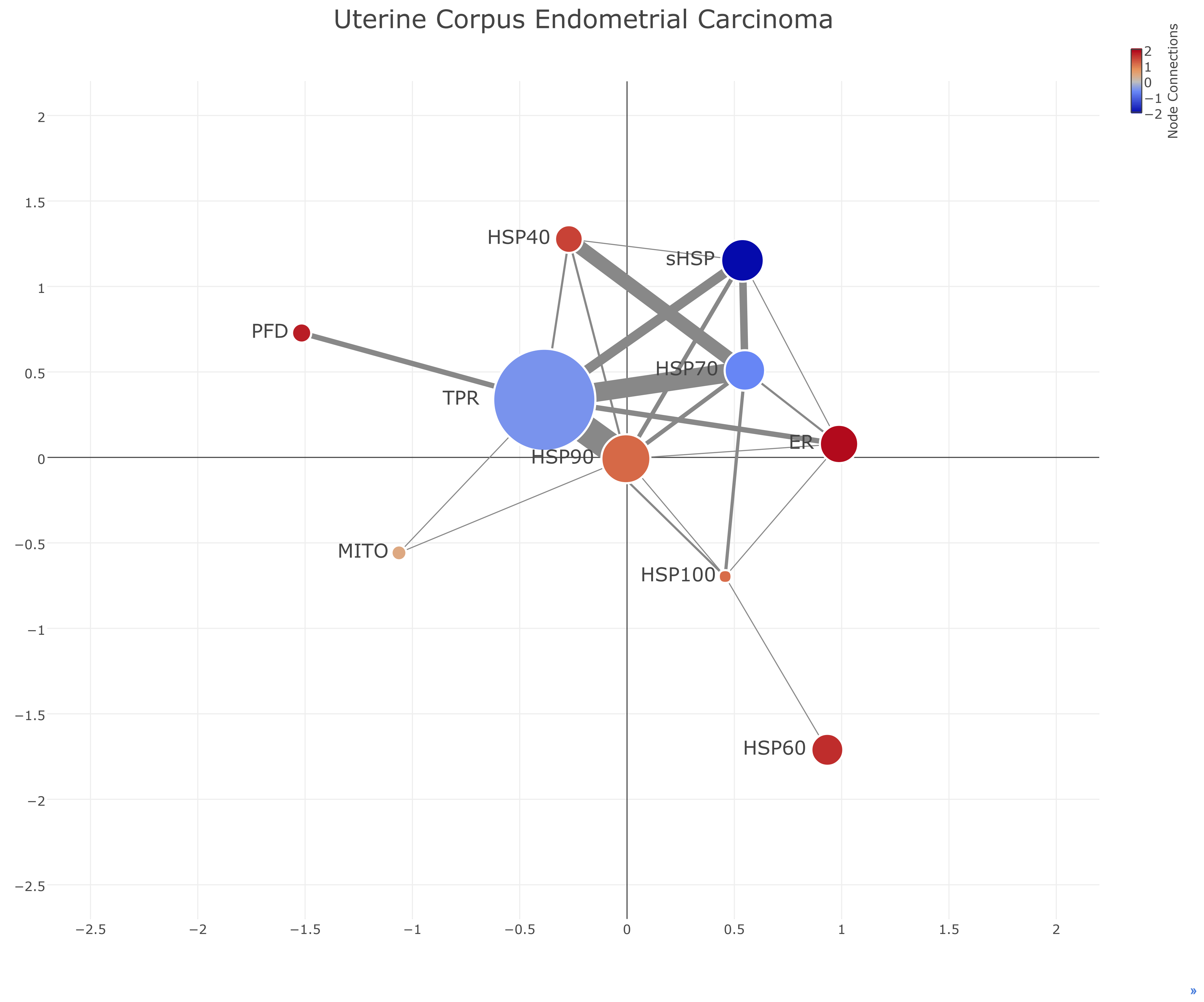
Uterine Corpus Endometrial Carcinoma
Node Connections
2
1
0
−1
−2
HSP40
sHSP
PFD
HSP70
TPR
ER
HSP90
MITO
HSP100
HSP60
2
1.5
1
0.5
0
−0.5
−1
−1.5
−2
−2.5
−2.5
−2
−1.5
−1
−0.5
0
0.5
1
1.5
2
»

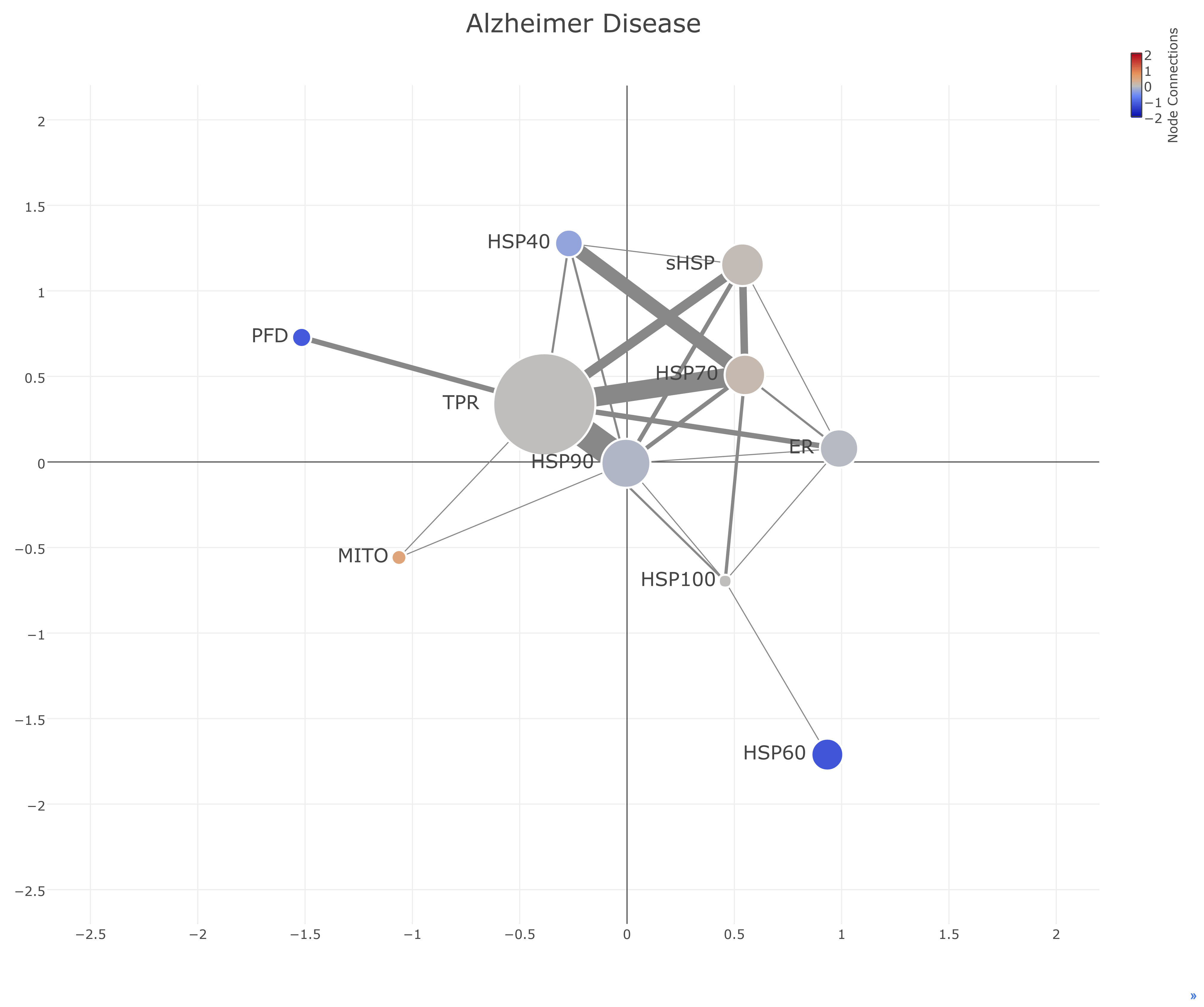
Alzheimer Disease
Node Connections
2
1
0
−1
−2
HSP40
sHSP
PFD
HSP70
TPR
ER
HSP90
MITO
HSP100
HSP60
2
1.5
1
0.5
0
−0.5
−1
−1.5
−2
−2.5
−2.5
−2
−1.5
−1
−0.5
0
0.5
1
1.5
2

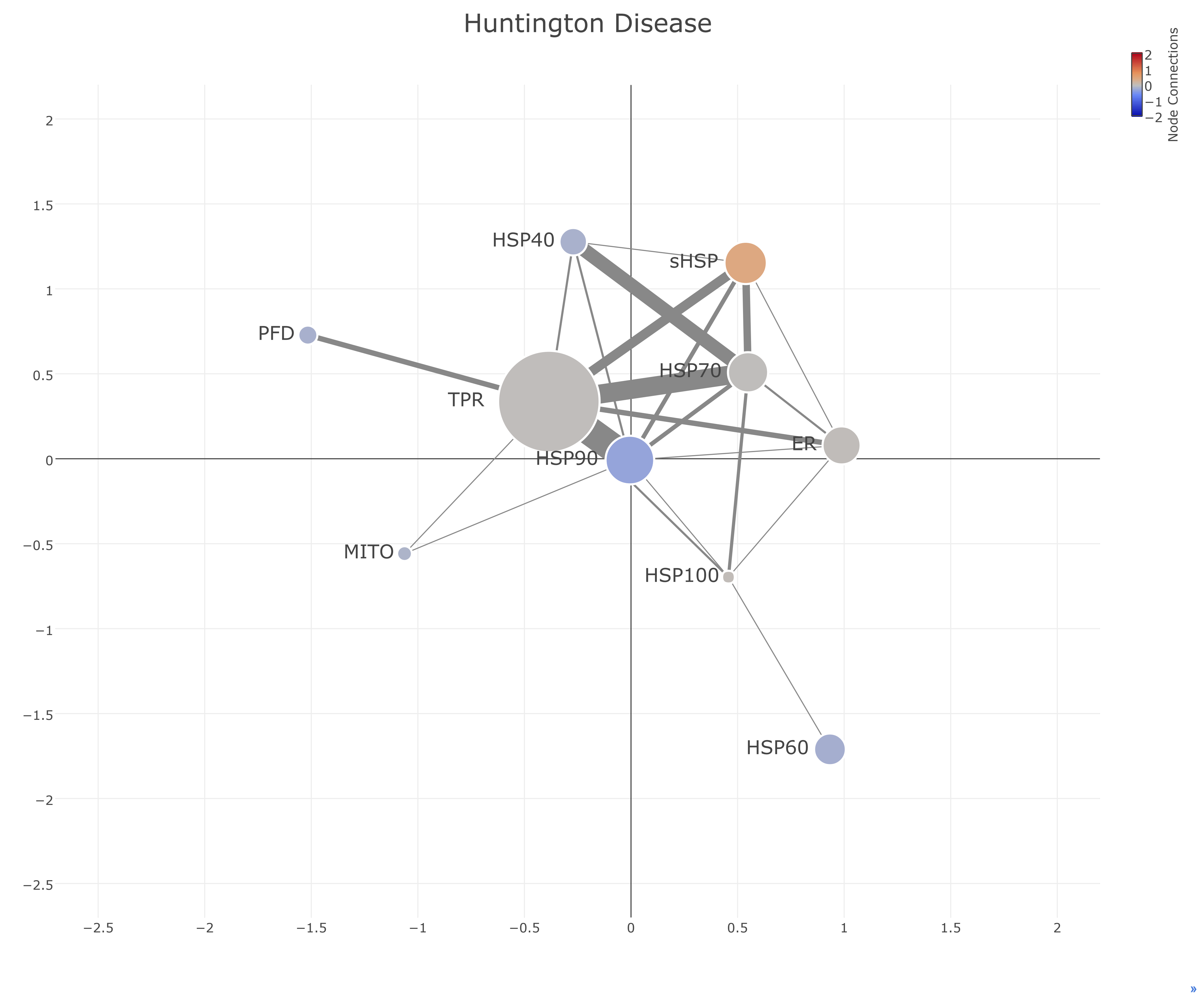

Huntington Disease
Node Connections
2
1
0
−1
−2
HSP40
sHSP
PFD
HSP70
TPR
ER
HSP90
MITO
HSP100
HSP60
2
1.5
1
0.5
0
−0.5
−1
−1.5
−2
−2.5
−2.5
−2
−1.5
−1
−0.5
0
0.5
1
1.5
2
»

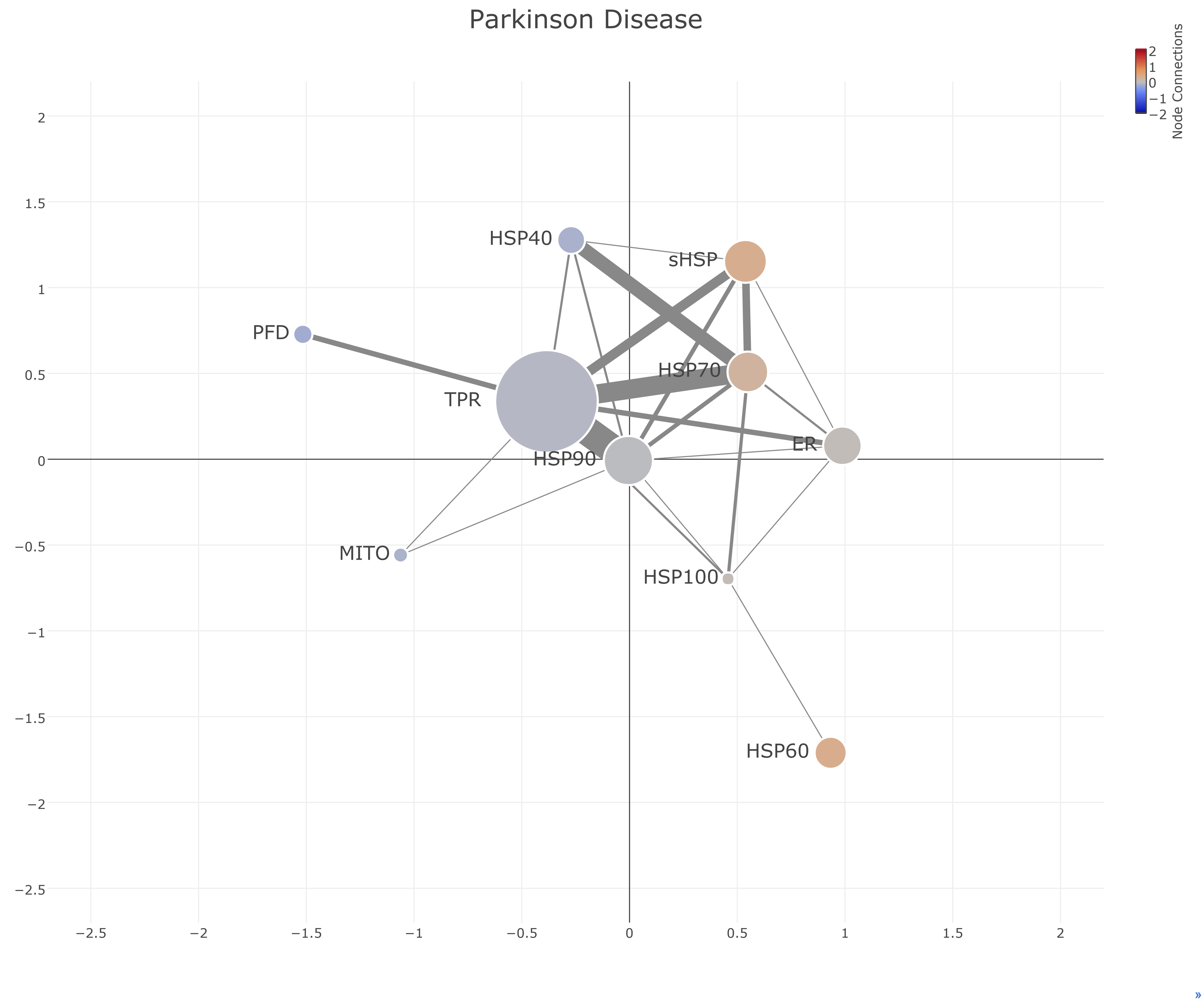
Parkinson Disease
Node Connections
2
1
0
−1
−2
HSP40
sHSP
PFD
HSP70
TPR
ER
HSP90
MITO
HSP100
HSP60
2
1.5
1
0.5
0
−0.5
−1
−1.5
−2
−2.5
−2.5
−2
−1.5
−1
−0.5
0
0.5
1
1.5
2
