# Supplementary material for: A systematic atlas of chaperome deregulation topologies across the human cancer landscape
Source: PLoS Comput Biol. 2018 Jan 2;14(1):e1005890. doi: 10.1371/journal.pcbi.1005890 (PMC5766242; doi:10.1371/journal.pcbi.1005890)

Figure S5

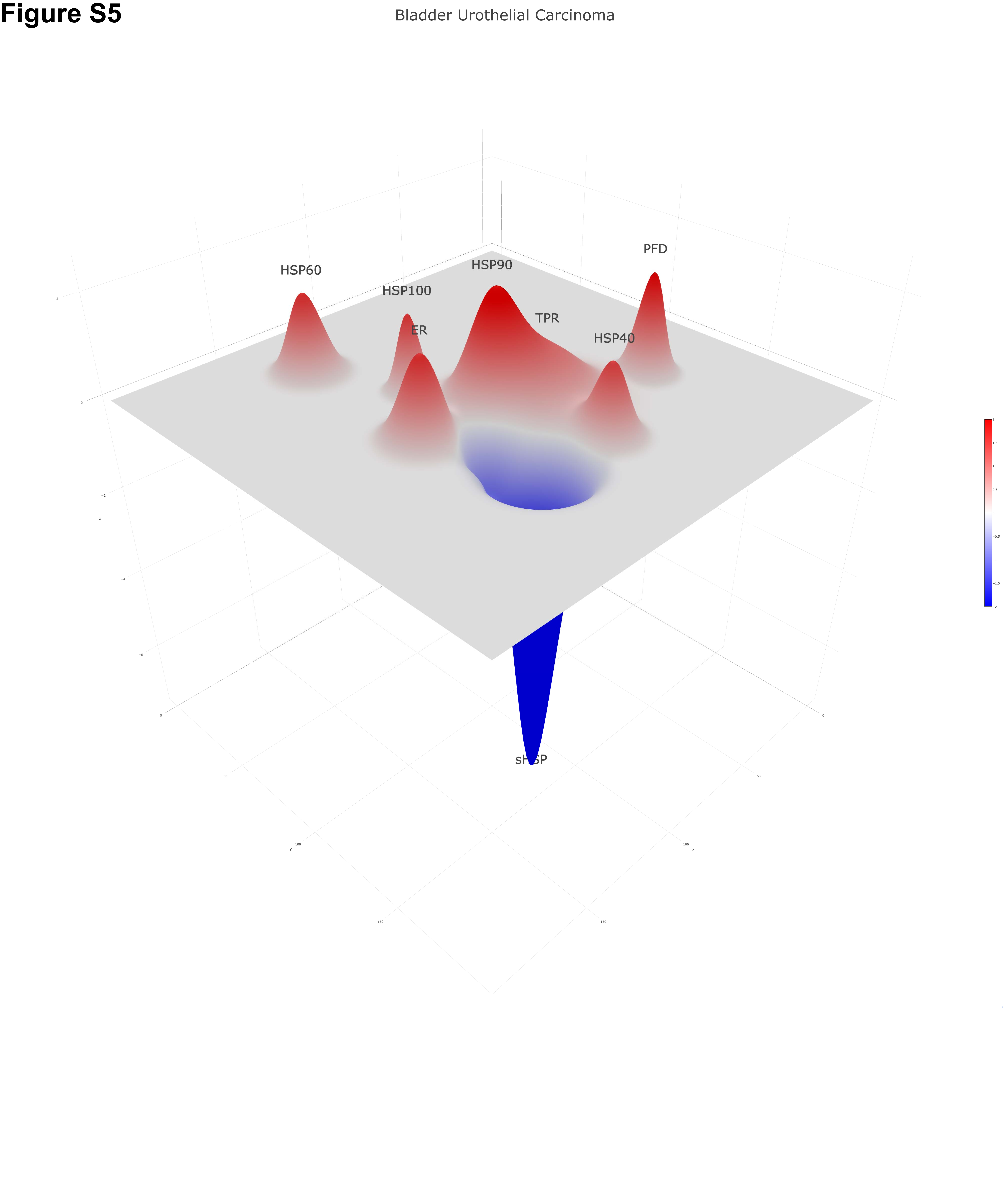
Bladder Urothelial Carcinoma
HSP60
HSP100
HSP90
PFD
TPR
ER
HSP40
sHSP

Breastinvasivecarcinoma

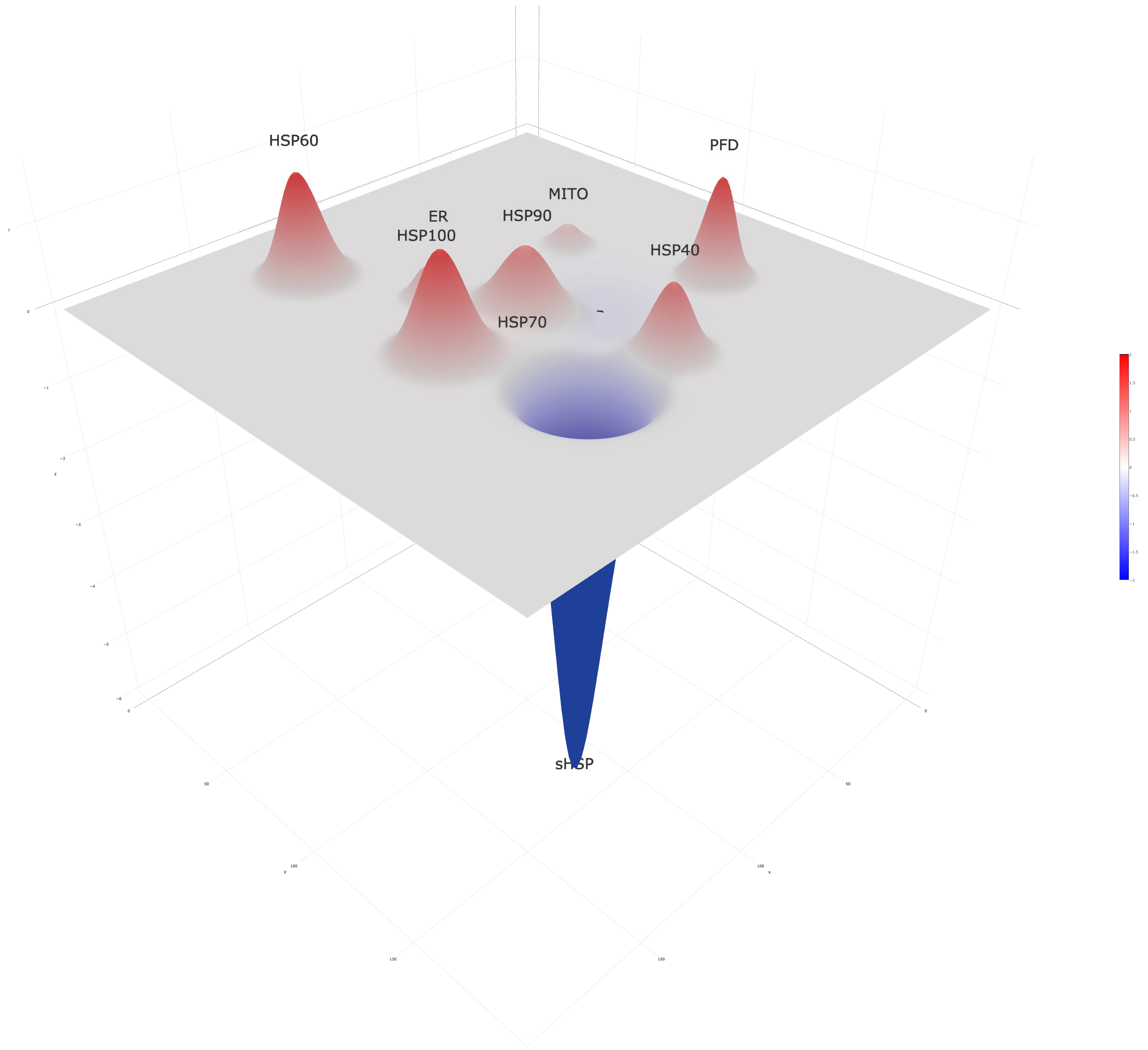

Cervicalsquamouscellcarcinomaandendocervicaladenocarcinoma

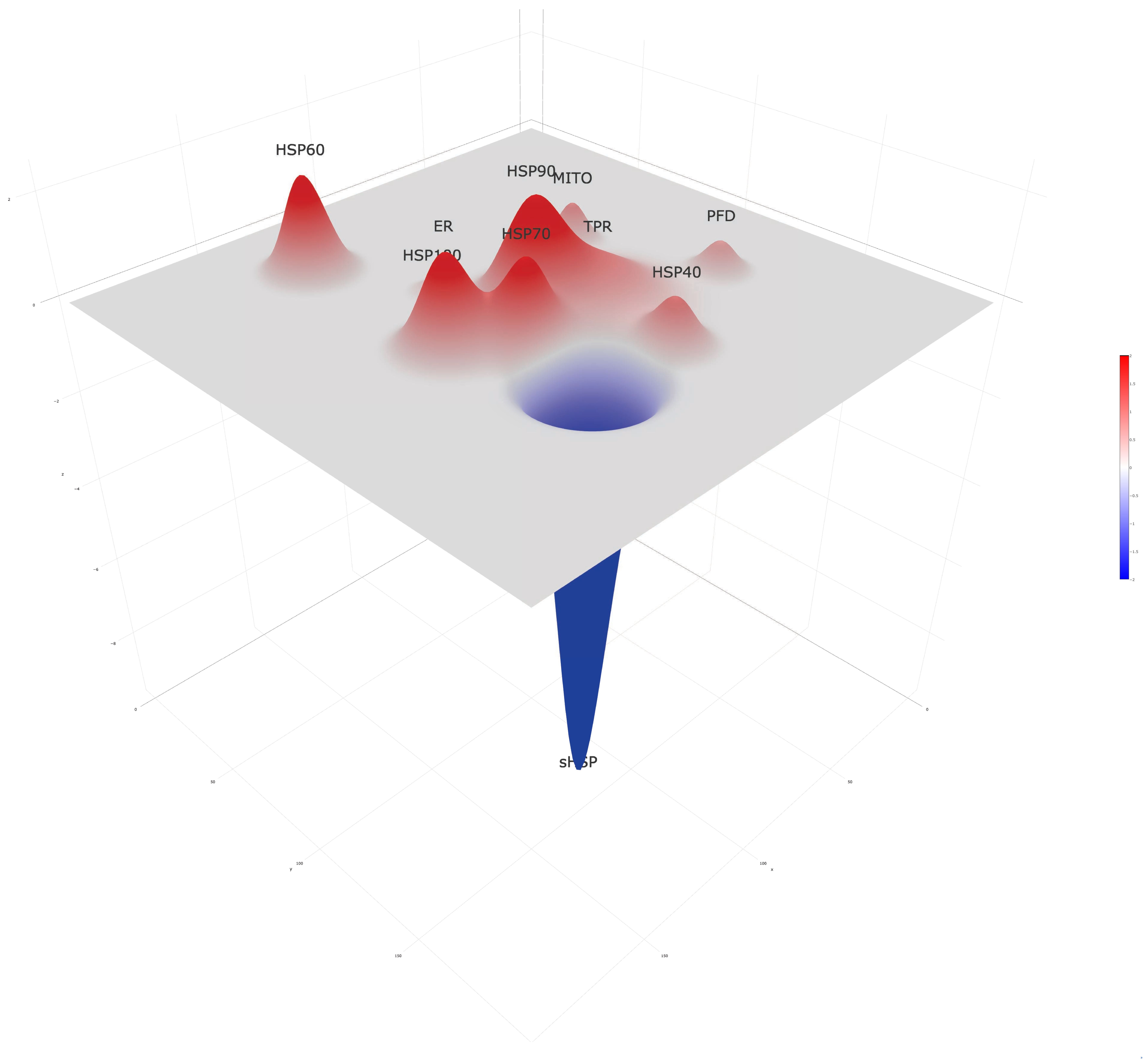

Cholangiocarcinoma

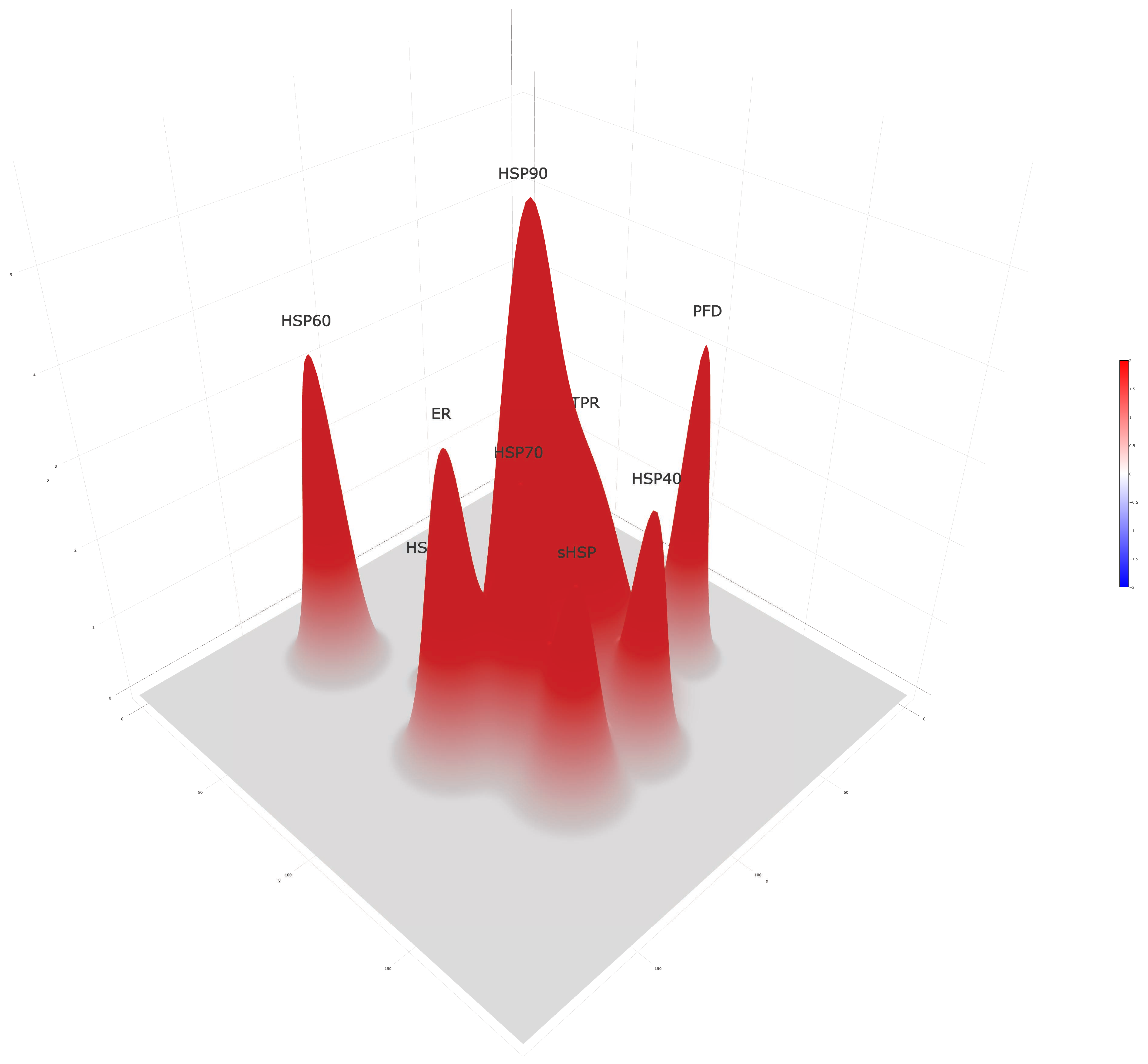

Esophagealcarcinoma

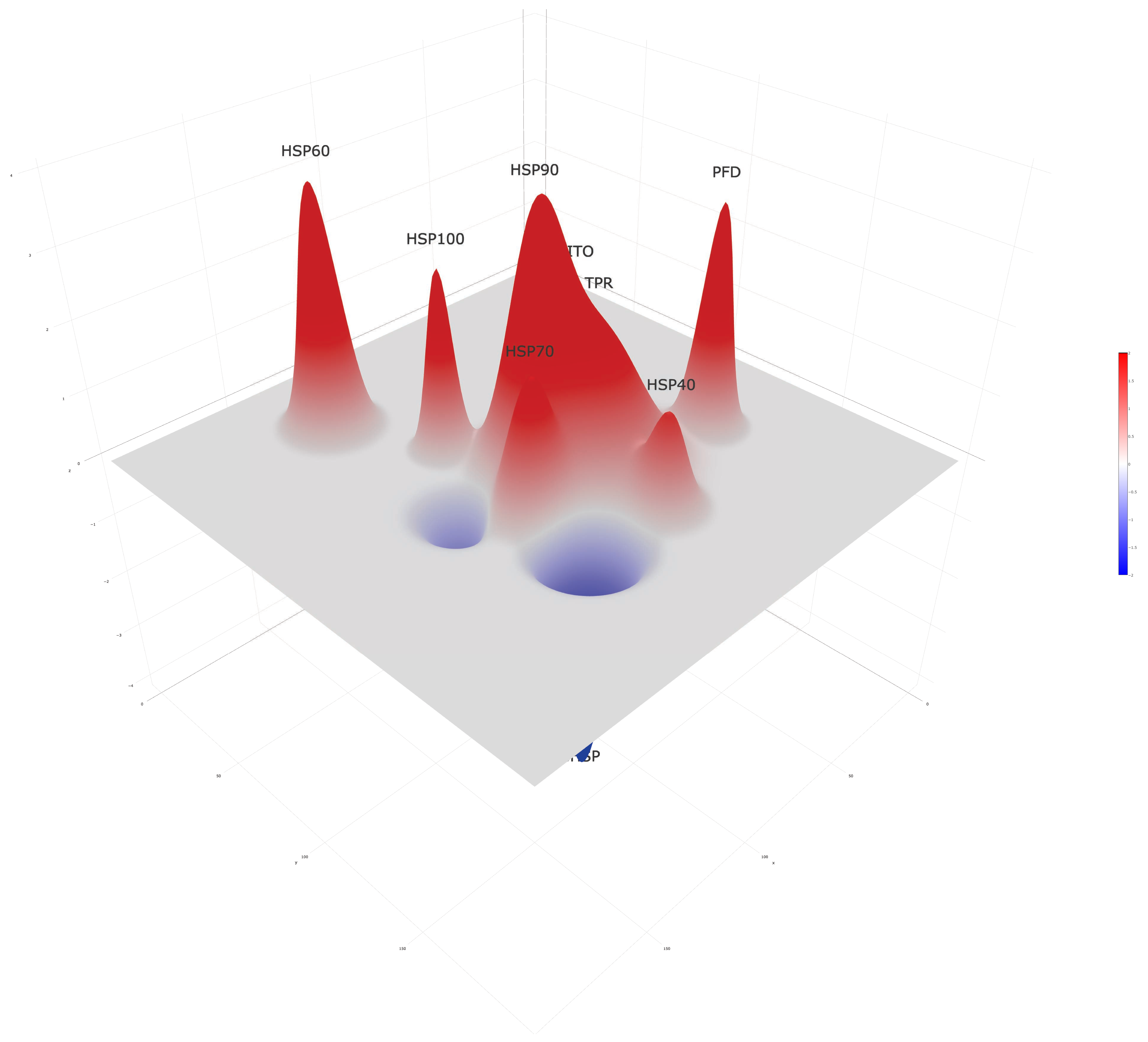

Glioblastomamultiforme

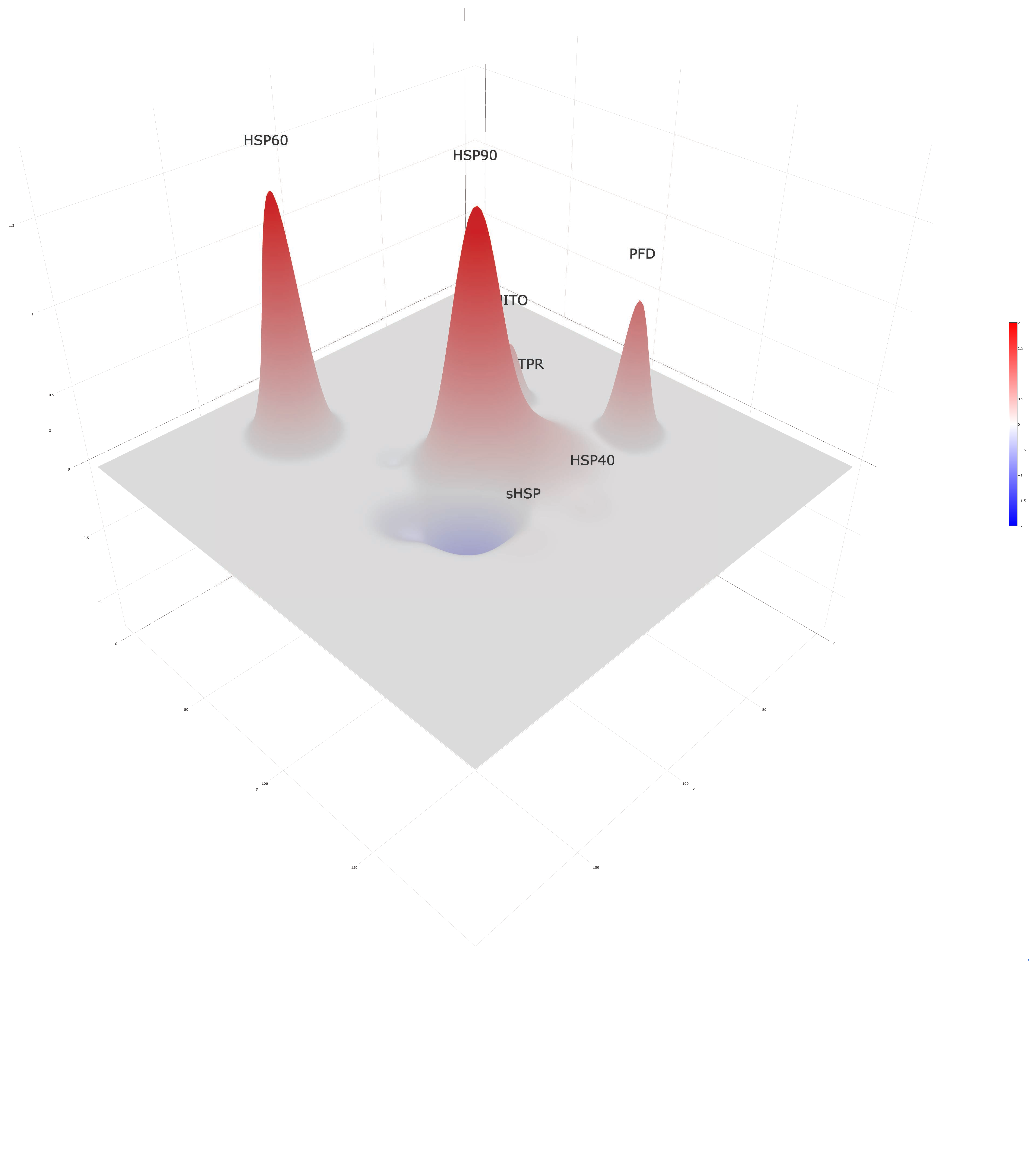

Headand Necksquamouscellcarcinoma

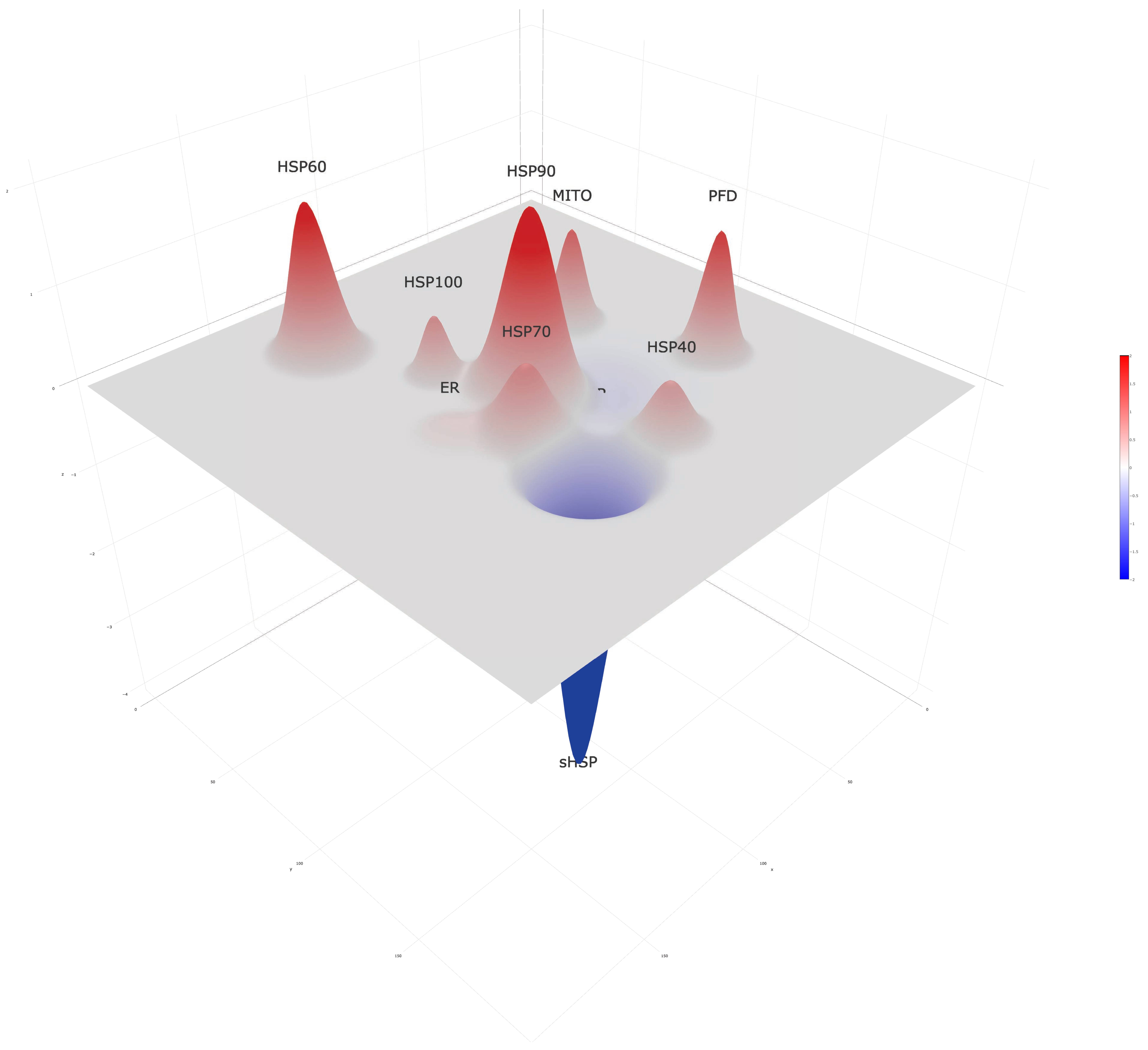

Kidney Chromophobe

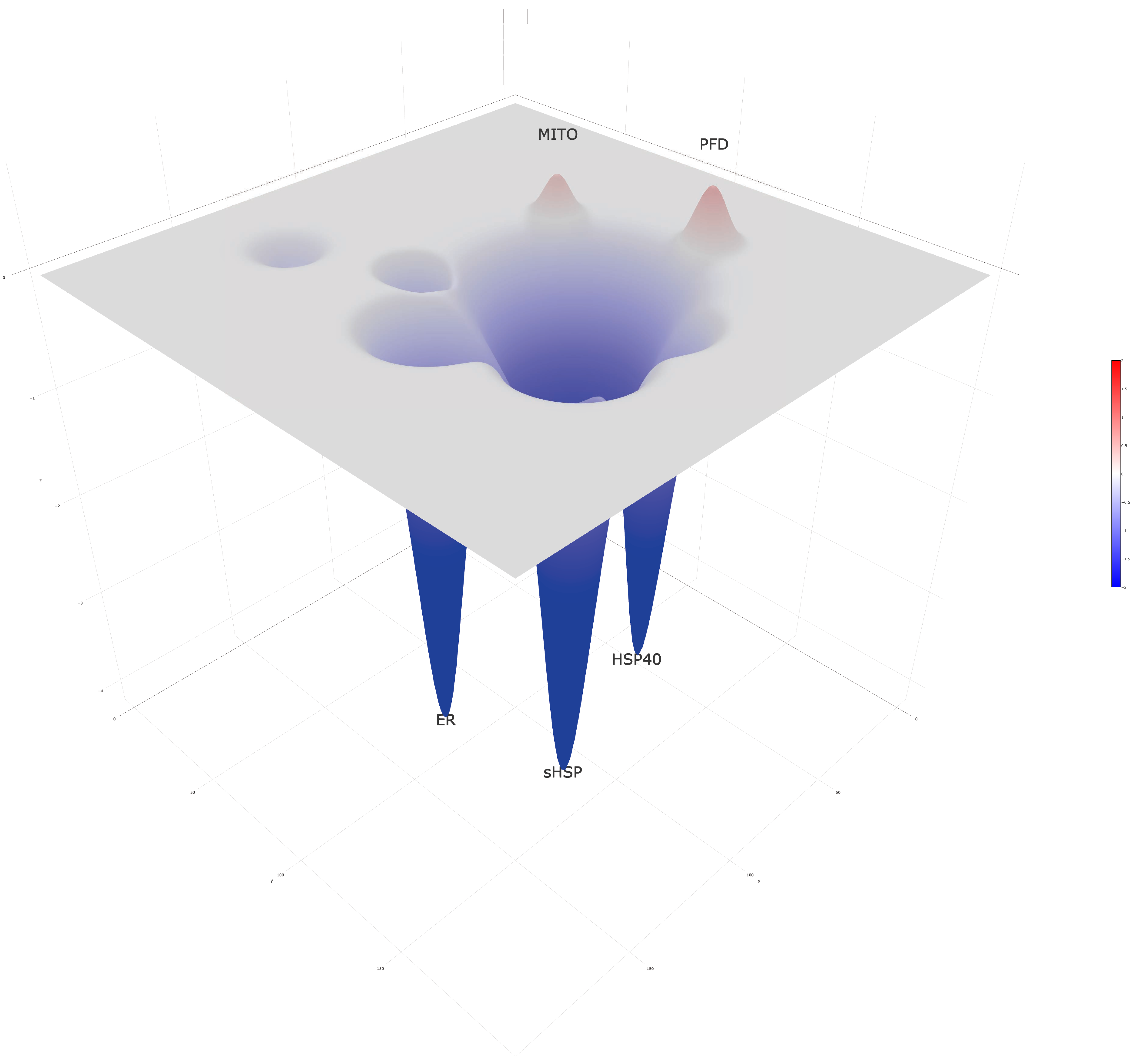

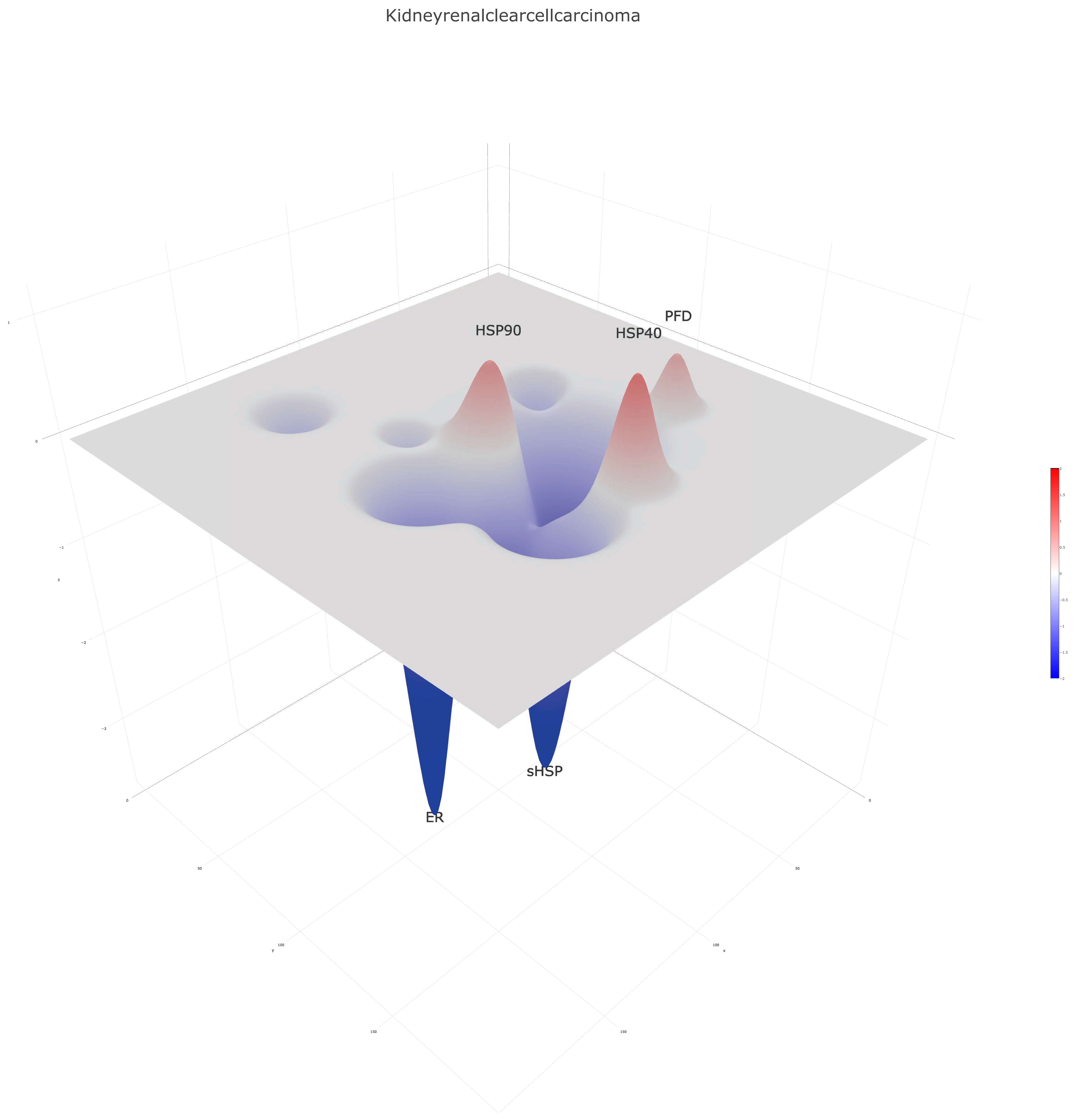
Kidneyrenalclearcellcarcinoma
HSP90
HSP40
PFD
sHSP
ER

Kidneyrenalpapillarycellcarcinoma

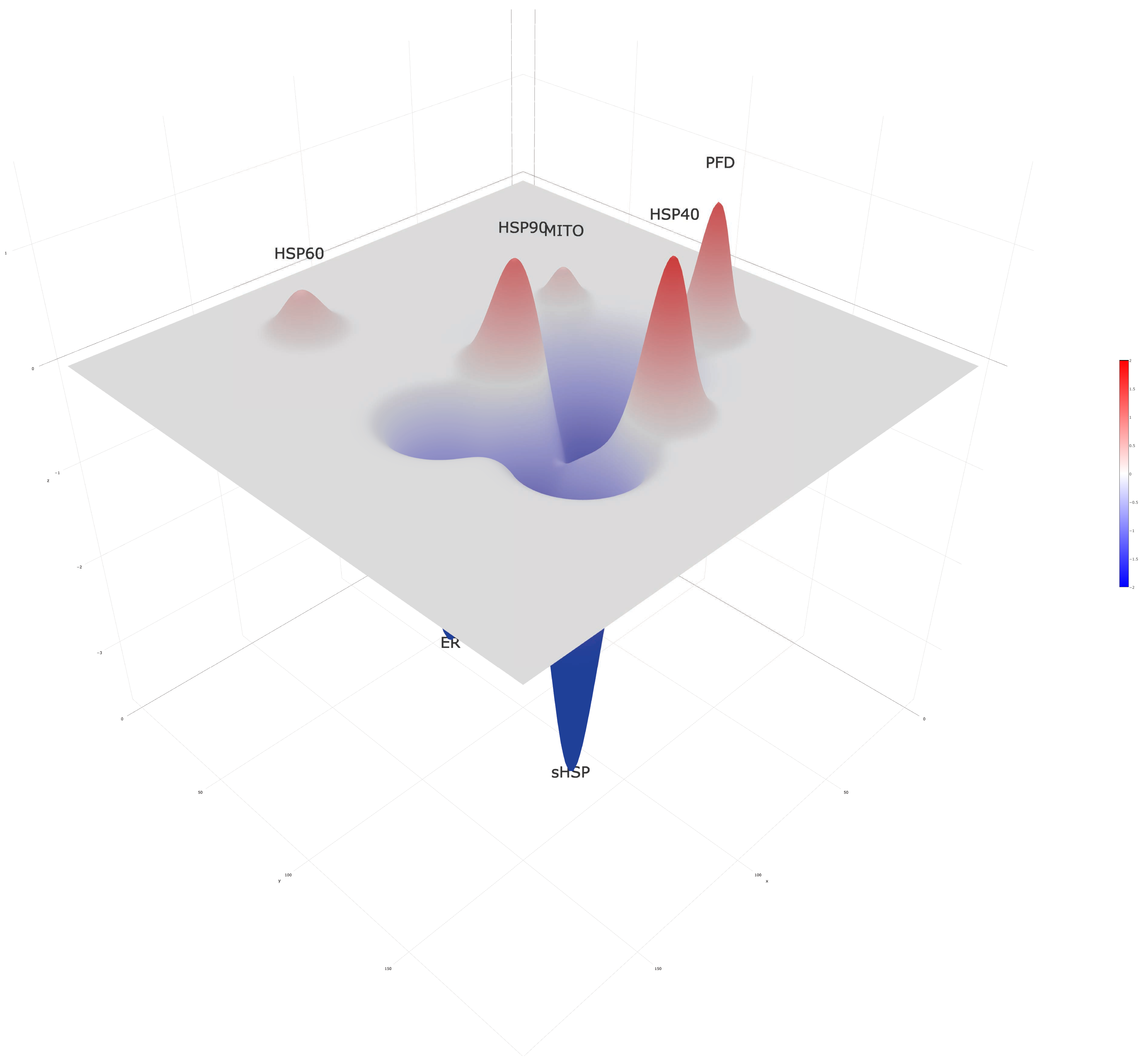

Liverhepatocellularcarcinoma

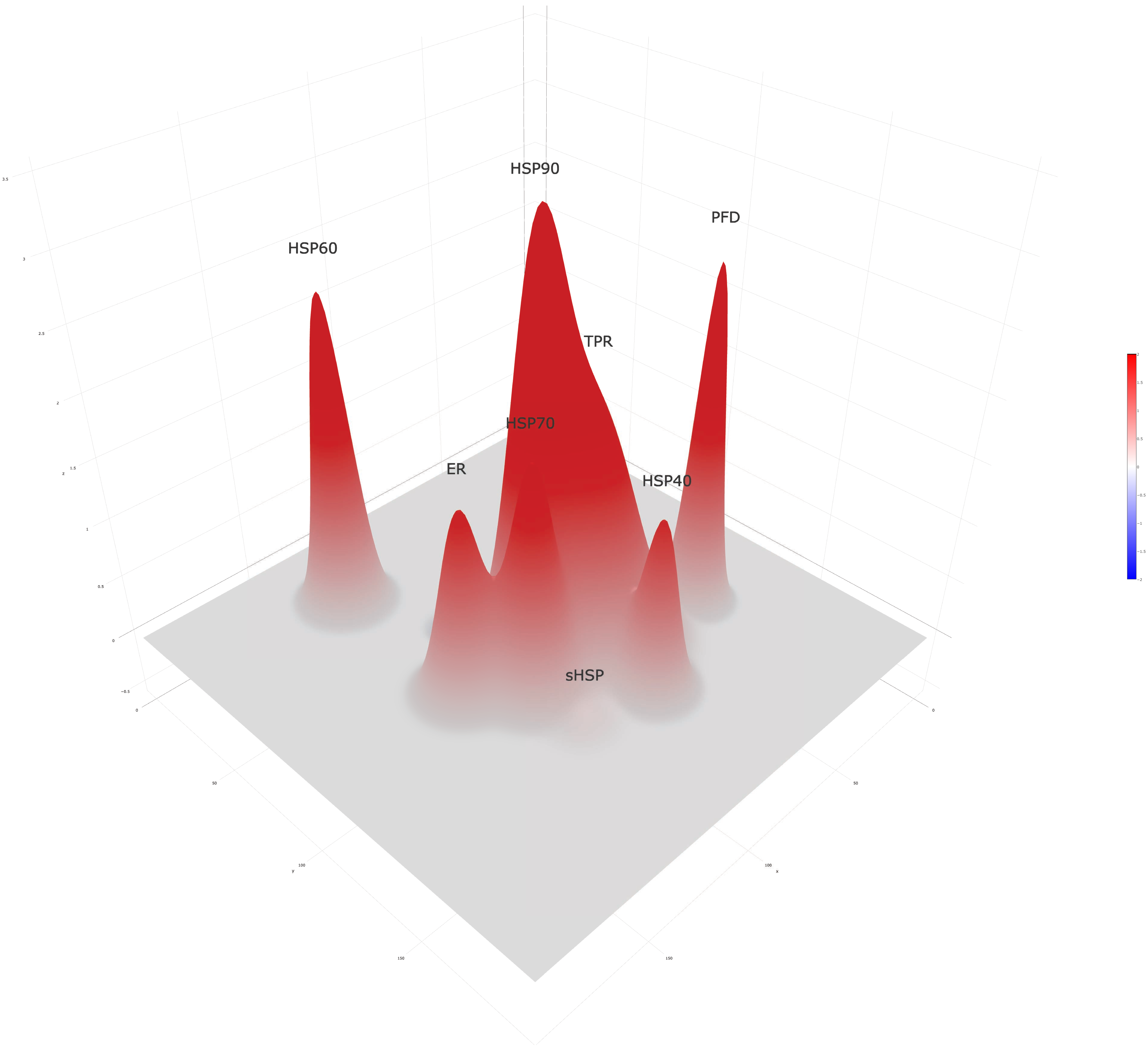

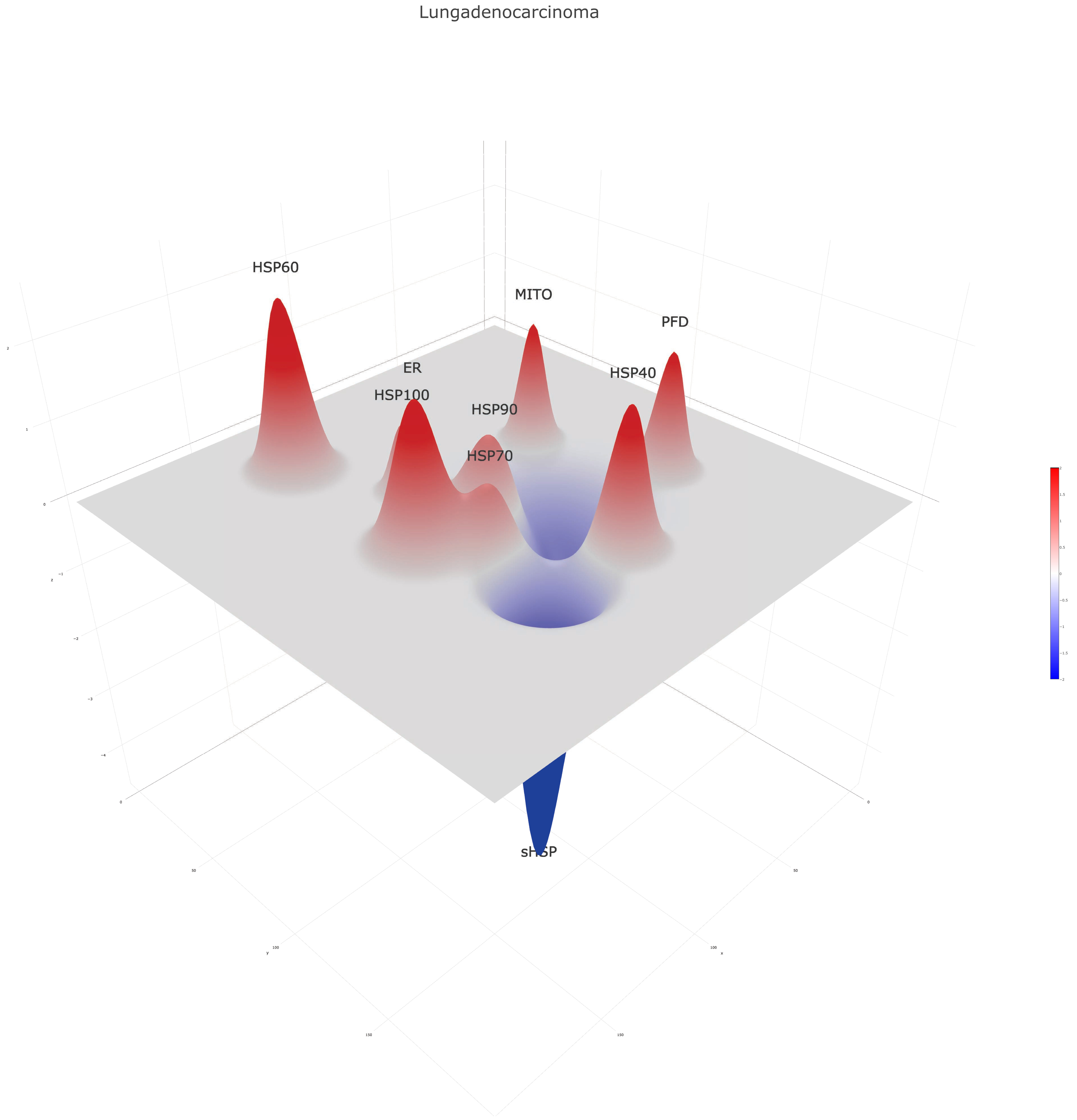
Lungadenocarcinoma
HSP60
MITO
PFD
ER
HSP40
HSP100
HSP90
HSP70
sHSP

Lungsquamouscellcarcinoma

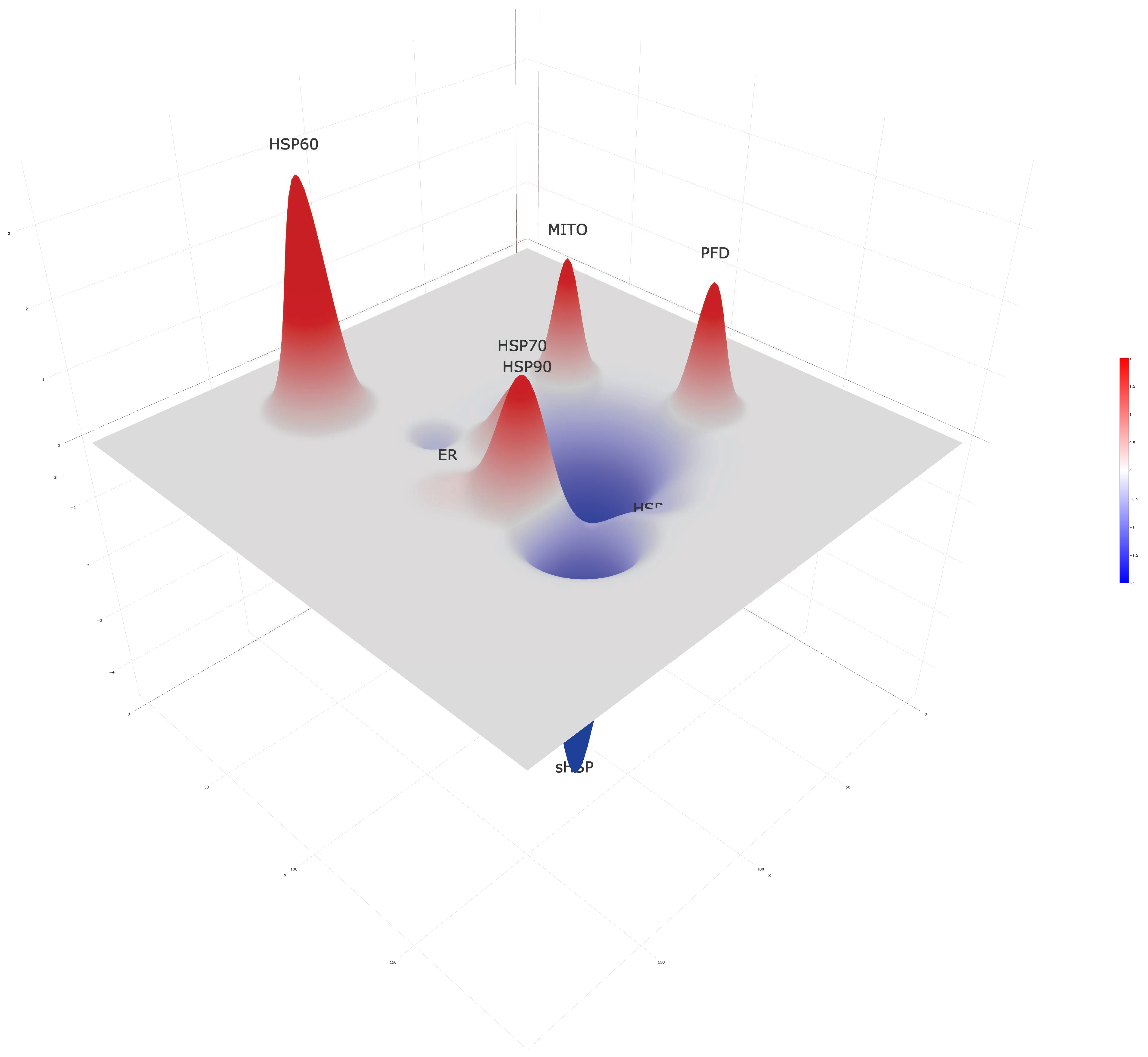

Pancreaticadenocarcinoma

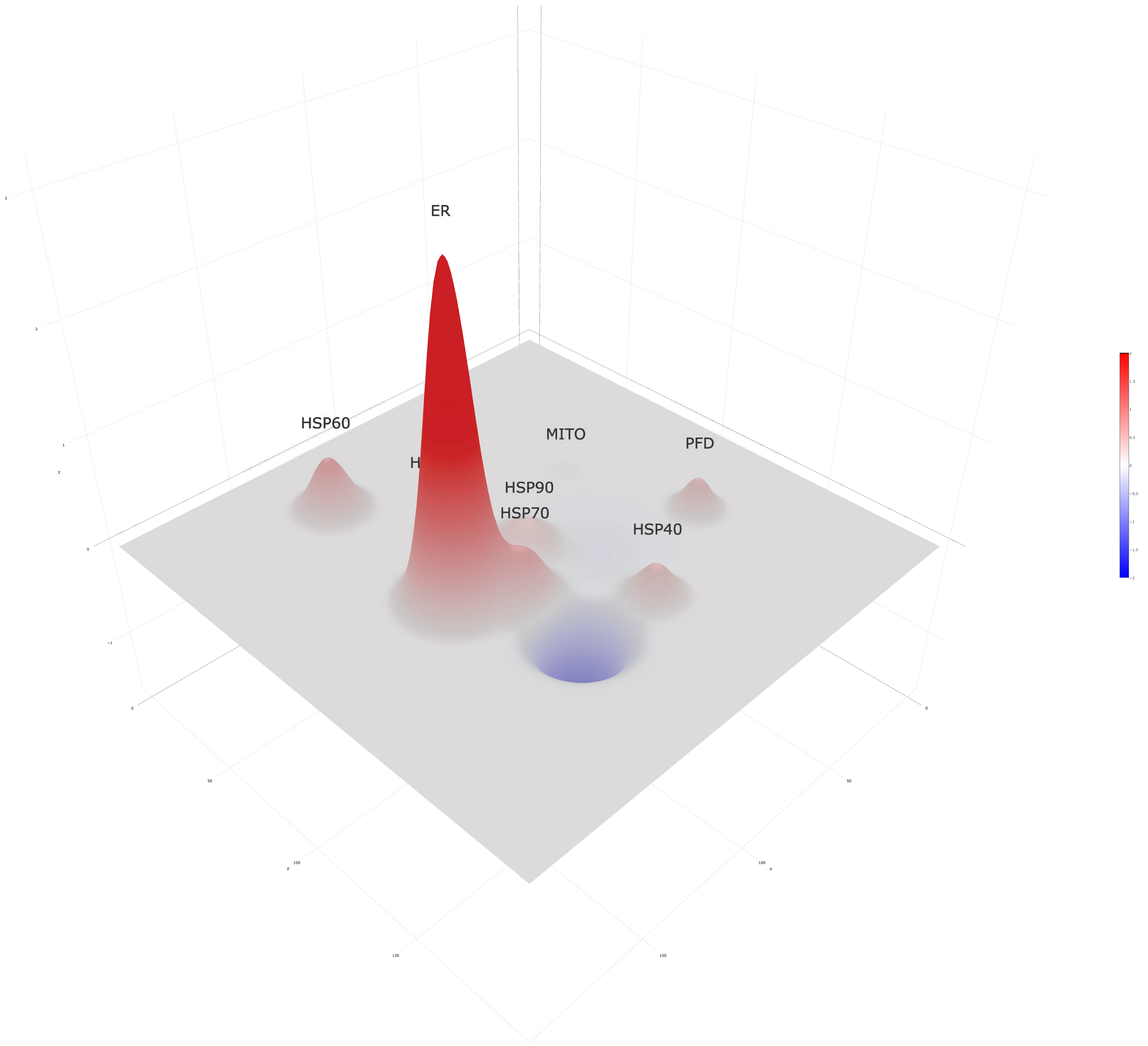

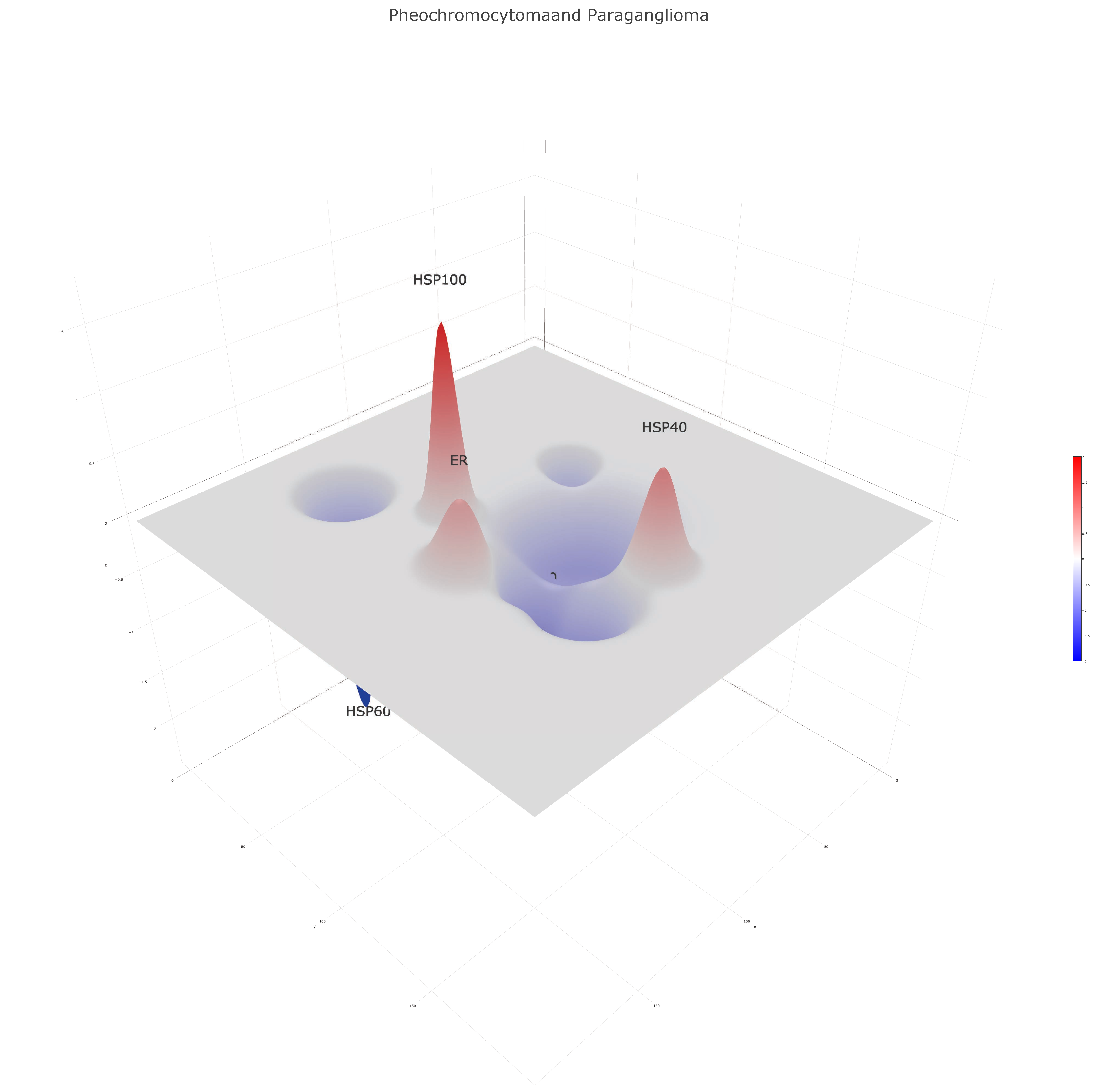
Pheochromocytomaand Paraganglioma
HSP100
HSP40
ER
HSP60
x
y
z

Prostateadenocarcinoma

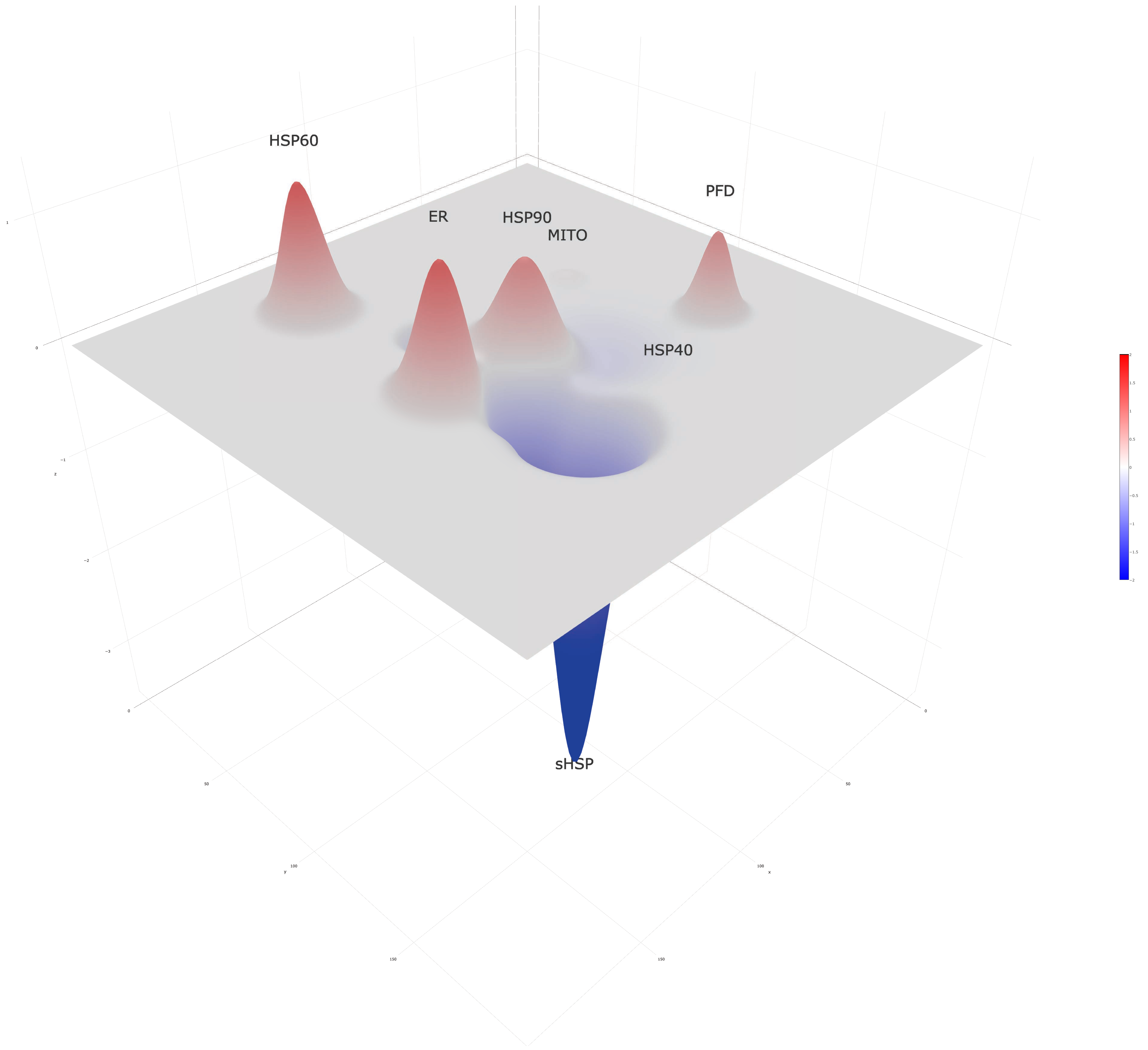

Sarcoma

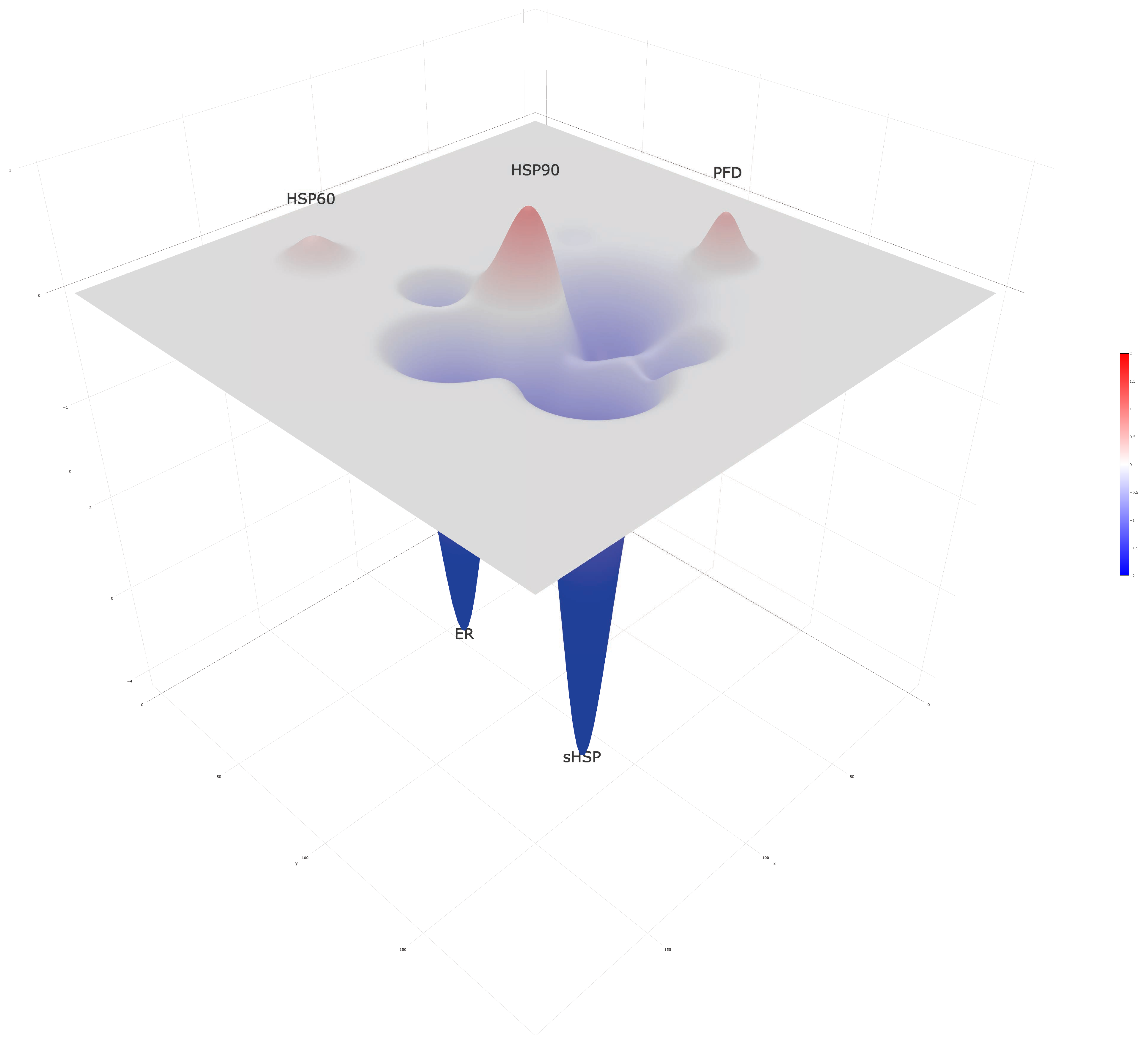

Skin Cutaneous Melanoma

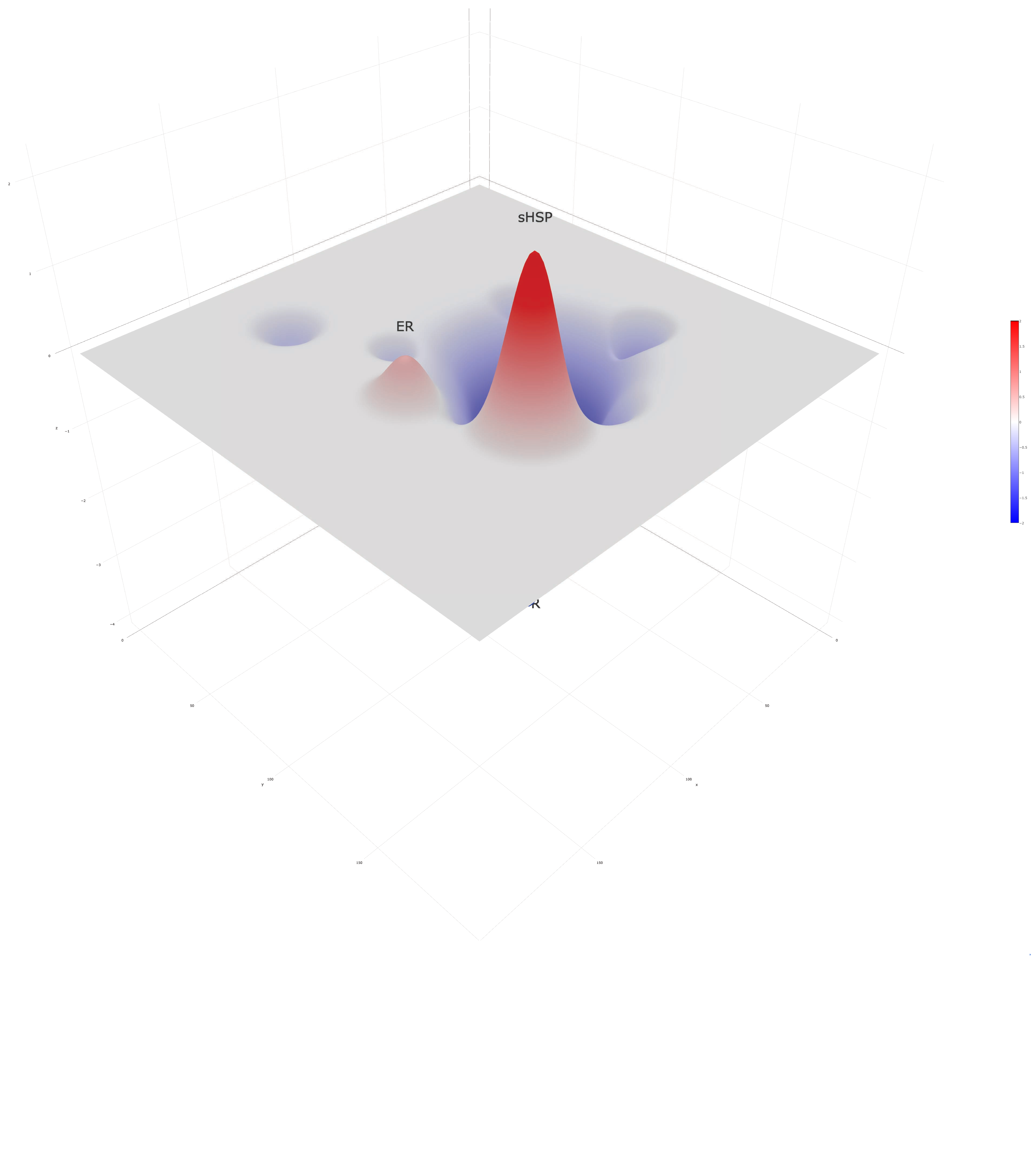

Stomachadenocarcinoma

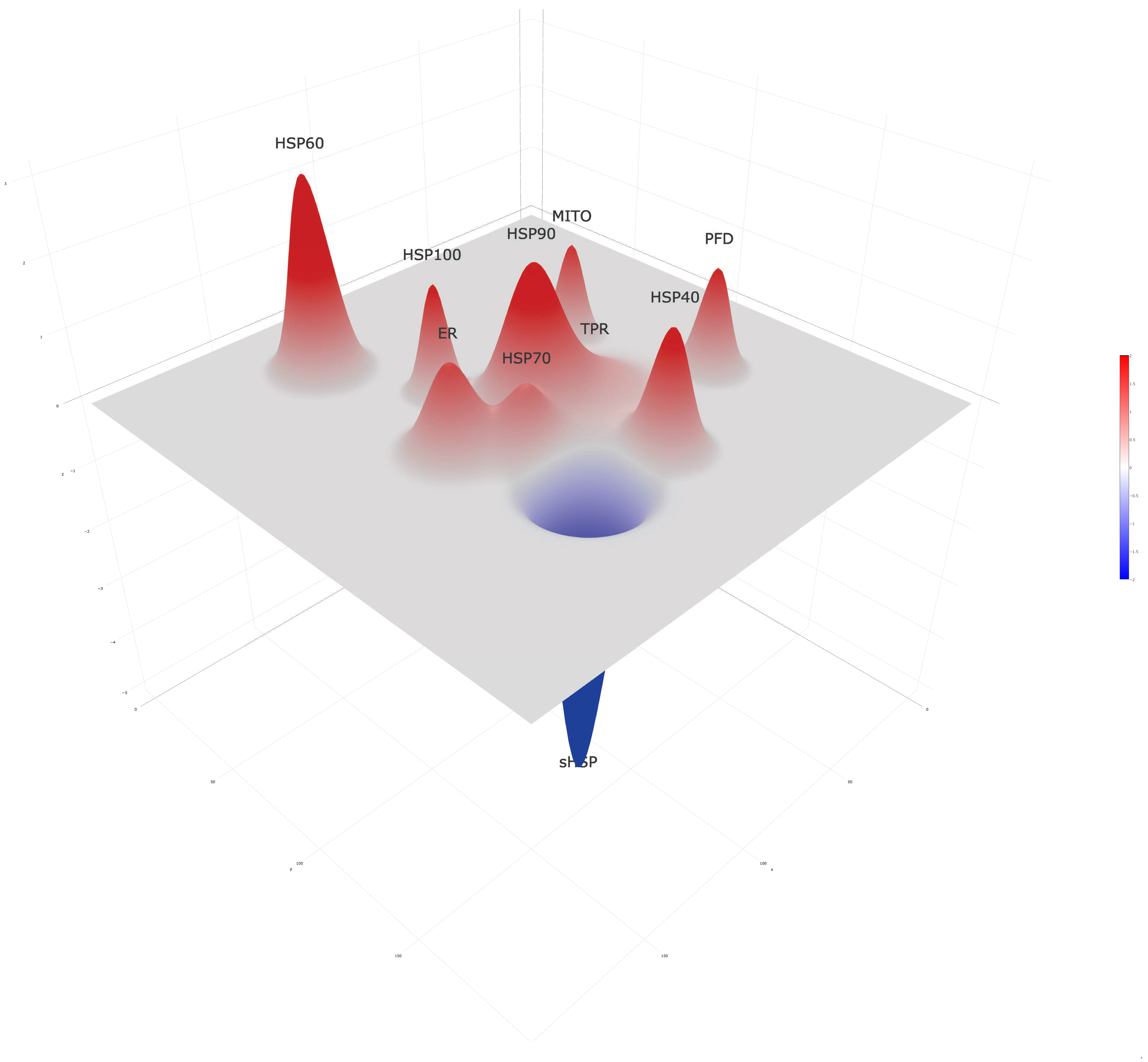

Thymoma

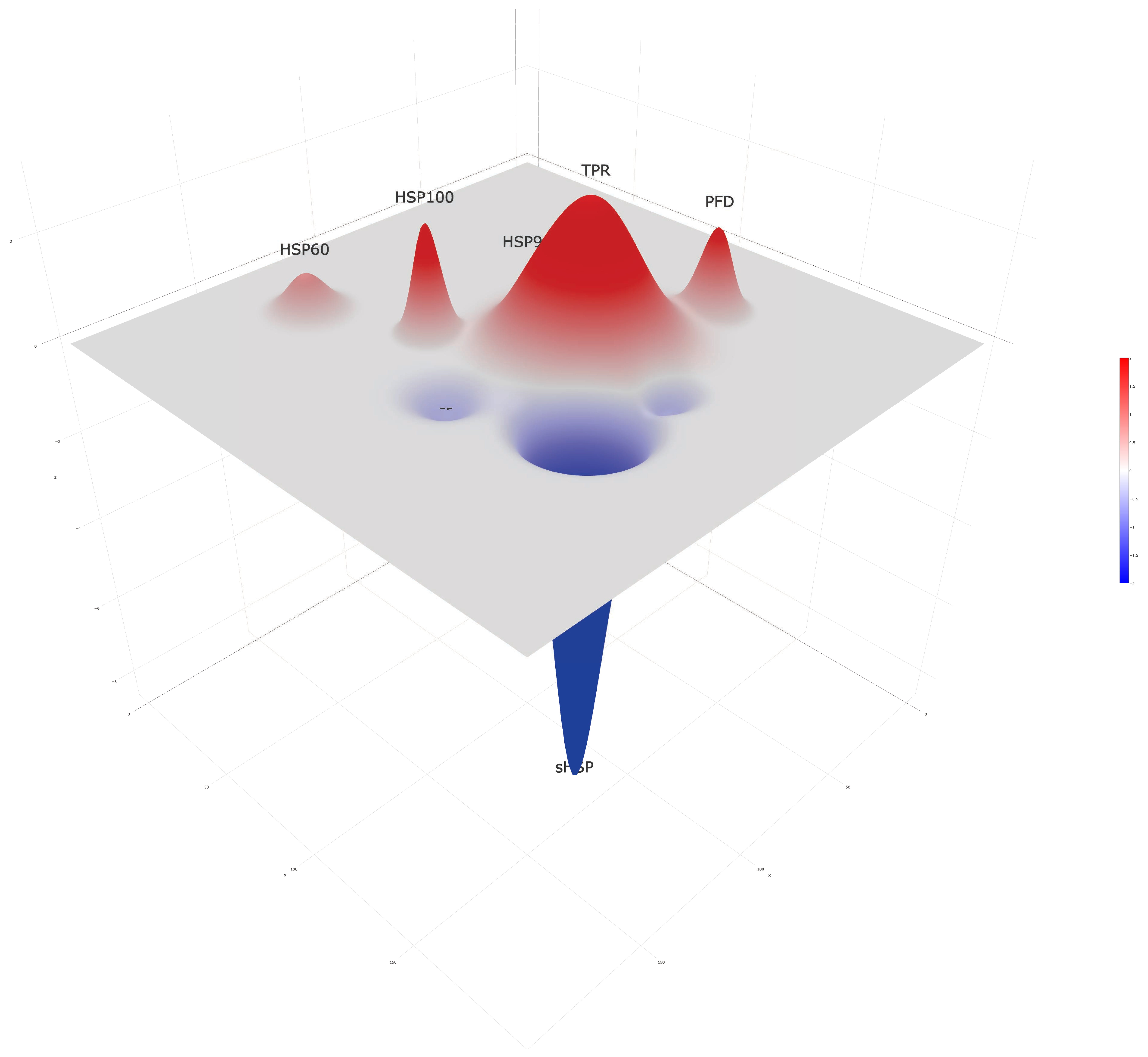

Thyroidcarcinoma

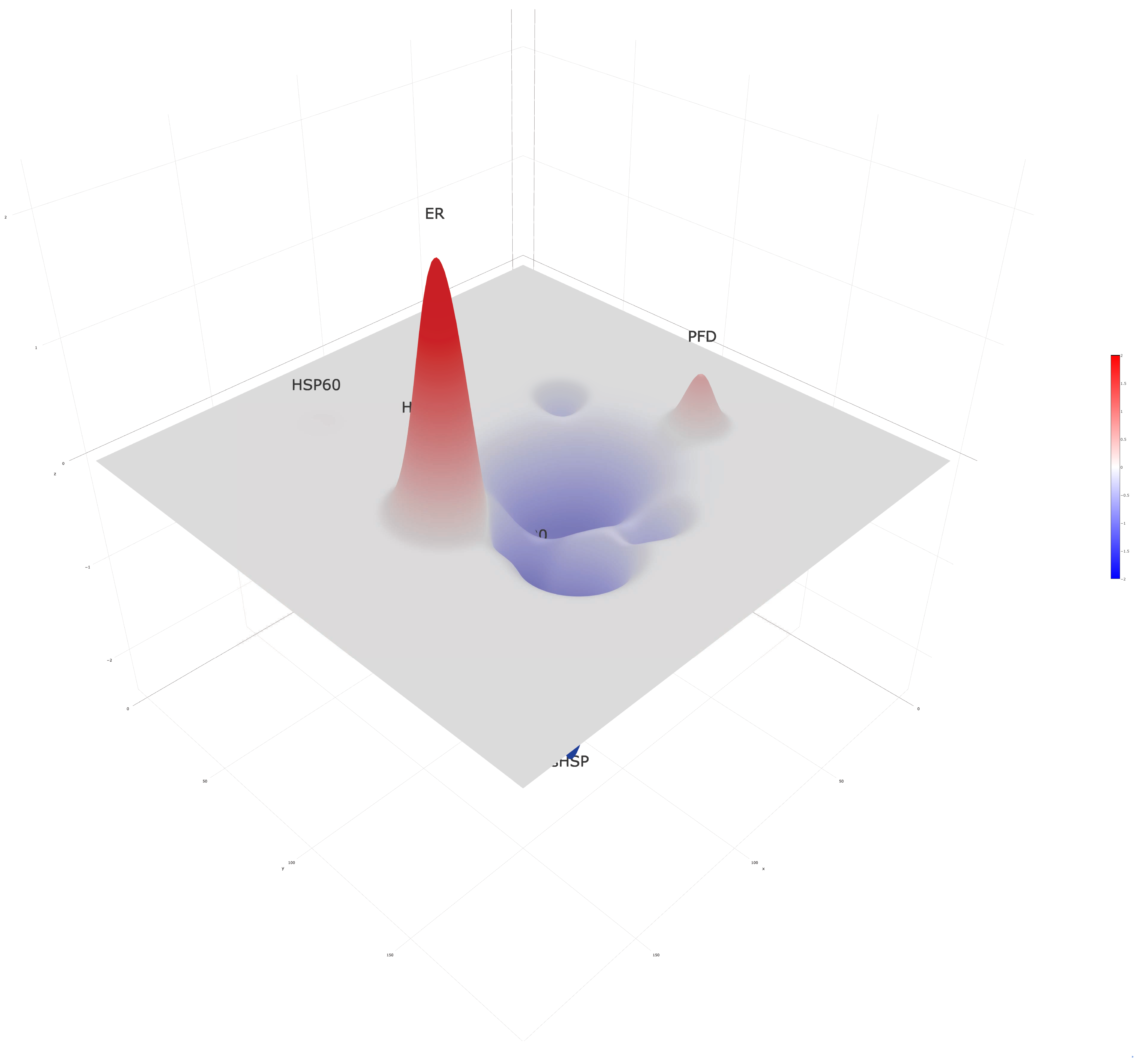

Uterine Corpus Endometrial Carcinoma

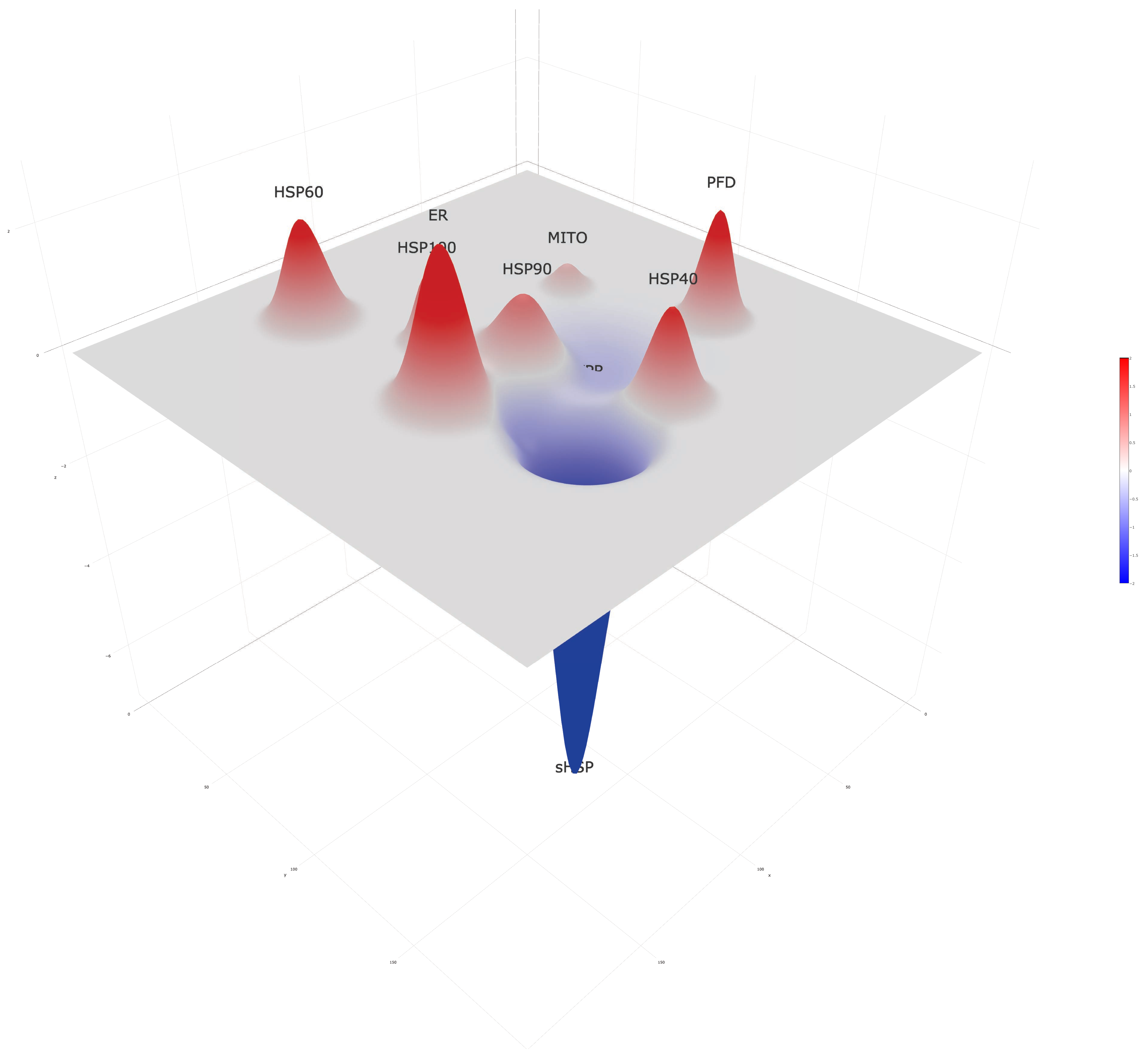

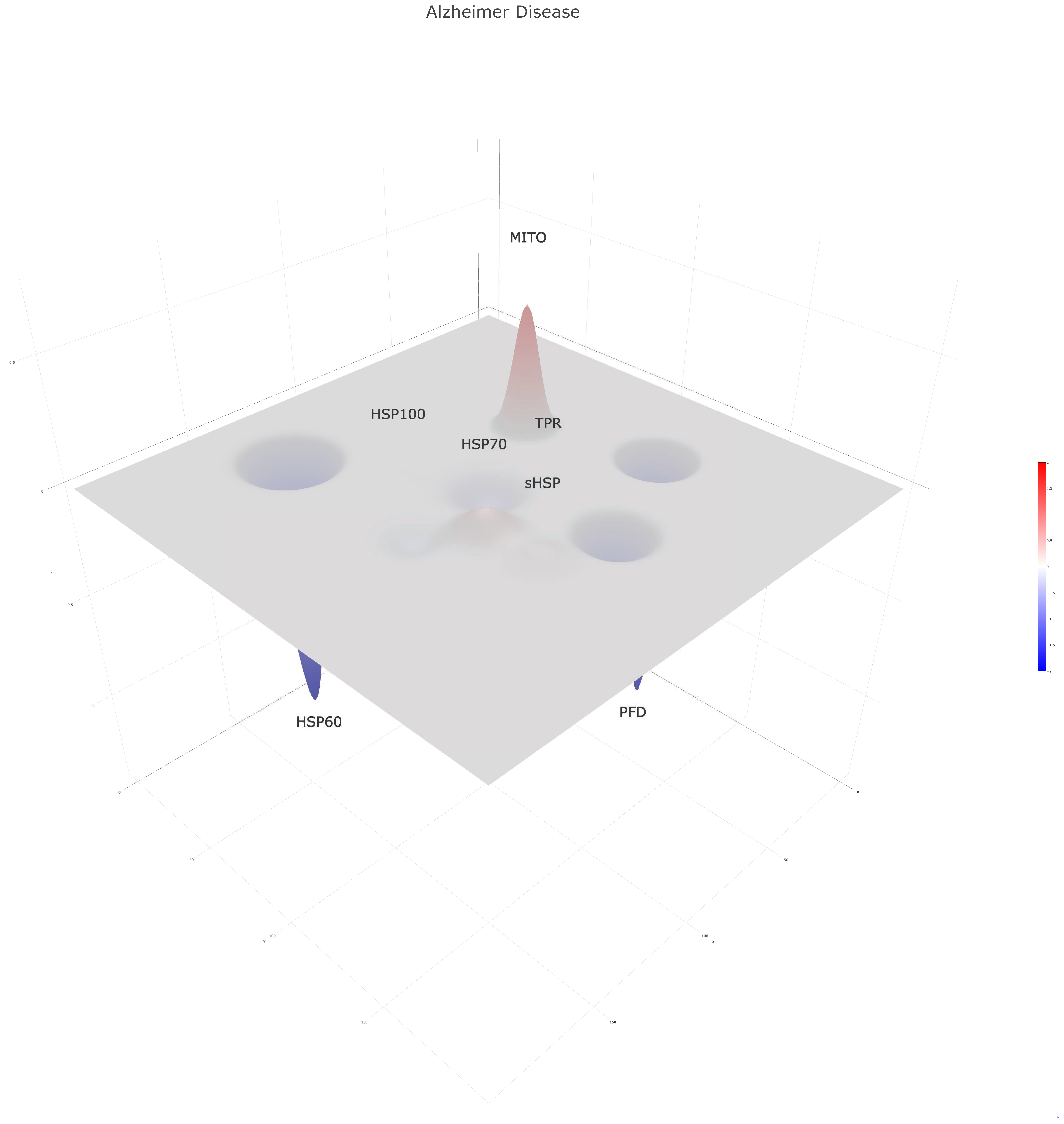

Alzheimer Disease
MITO
HSP100
TPR
HSP70
sHSP
HSP60
PFD

Huntington Disease

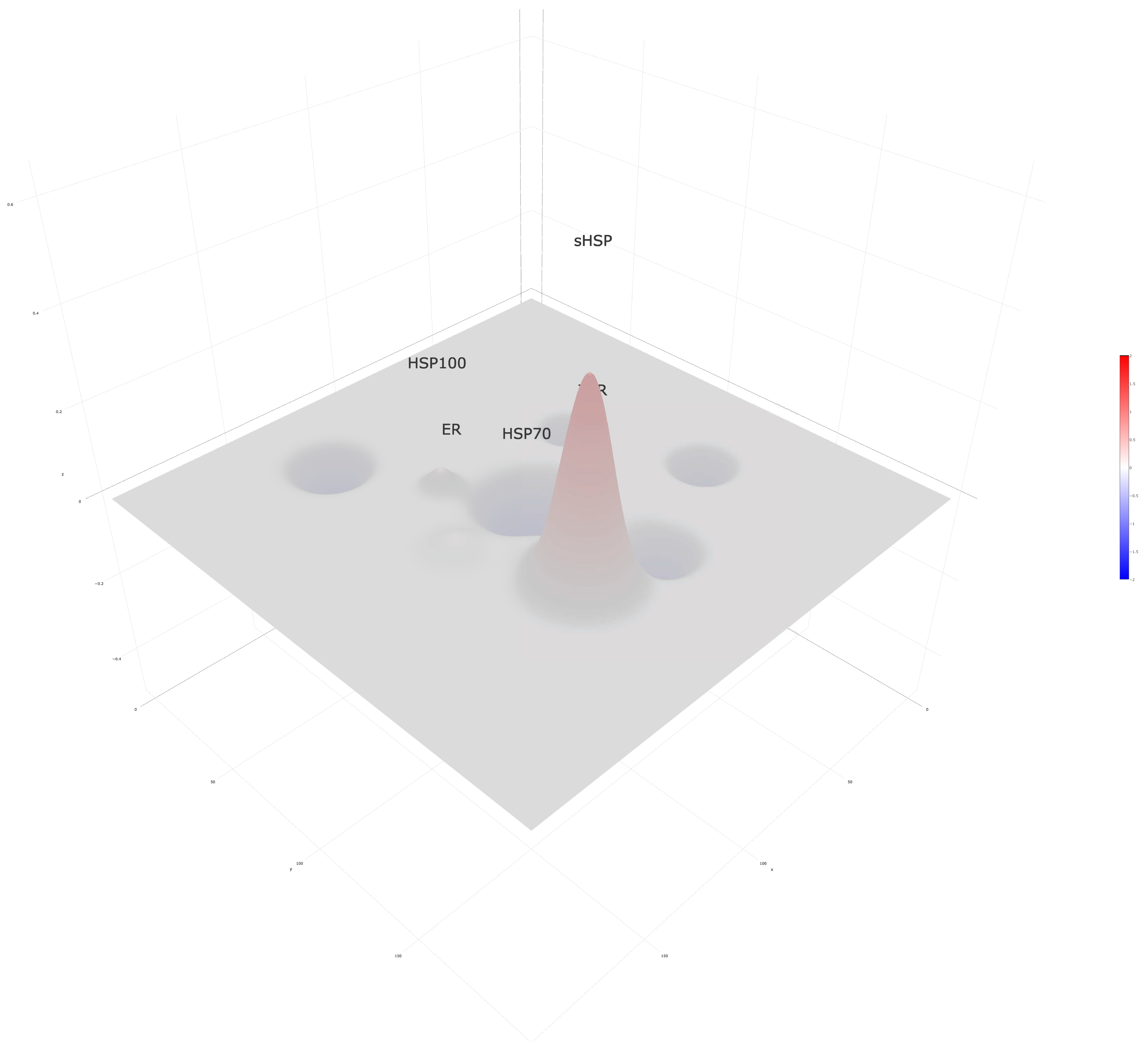

Parkinson Disease

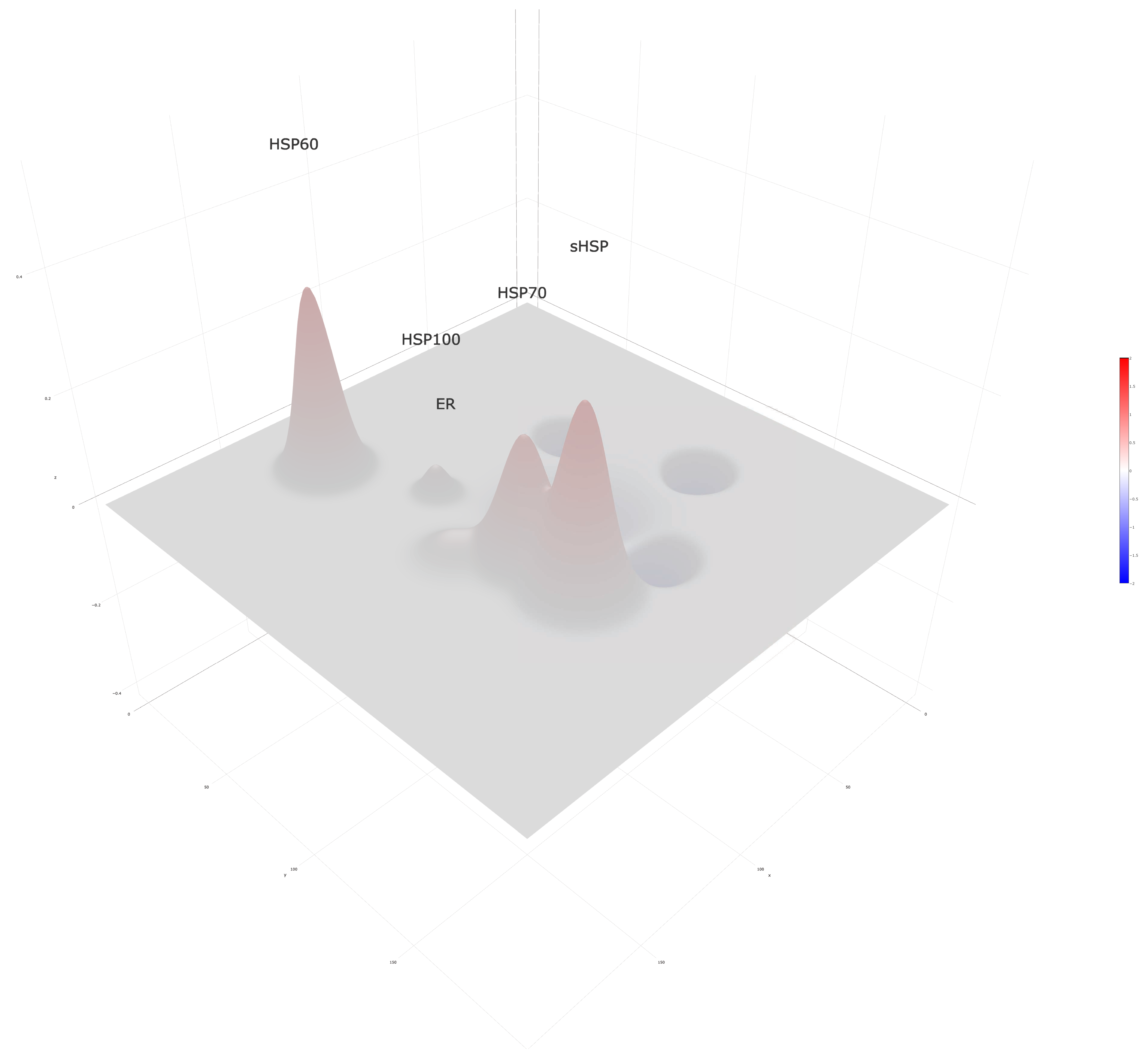

Supplement: S5 Fig — 3D topographic maps of cancer (pp. 1–22) or AD, PD and HD (pp. 23–25) chaperome alterations are obtained by projecting gene expression changes between disease and healthy counterpart biopsy gene expression (z dimension) onto the high-confidence chaperome meta-interactome (ME-CHAP) graph layout (x-y dimensions). (PDF) [file pcbi.1005890.s005.pdf]
