# Supplementary material for: A systematic atlas of chaperome deregulation topologies across the human cancer landscape
Source: PLoS Comput Biol. 2018 Jan 2;14(1):e1005890. doi: 10.1371/journal.pcbi.1005890 (PMC5766242; doi:10.1371/journal.pcbi.1005890)
Supplement: S1 Text — (DOCX) [file pcbi.1005890.s008.docx]

**S1 Text:**

## **On the correlation between RNA-seq and protein abundance, and its effect on studying the chaperome in cancer:**

Despite focusing on the chaperome, an ensemble of specific proteins involved in folding, we used gene expression data (RNA-seq) as input in our study rather than proteomics data. This decision is based on our aim of systematically studying chaperome alteration across cancers. In order to obtain robust results from our statistical methods and models, large amount of data on all cancers are necessary. Thus, the great abundance of public genomics data compared to their proteomics counterparts convinced us to focus on gene expression. Notwithstanding that, it is well known that gene expression and protein abundance do not always correlate well, with different results depending on tissue type, developmental state, stress conditions, genes considered, etc.

As a justification to use gene expression data in our study, we compared our results that were obtained analysing RNA-seq gene expression data to the outcome of the same method, but using protein abundance as input instead, in a scarce group of cancers where both, gene expression and protein abundance, were available in an acceptable quantity.

To our knowledge, there are only two databases containing proteomics data on samples from The Cancer Genome Atlas (TCGA): The Cancer Proteome Atlas (TCPA) and Clinical Proteomic Technology Assessment for Cancer (CPTAC). TCPA proteomics data covers a broad range of TCGA cancer groups, but does not include abundance measurement on a single chaperone. CPTAC on the other hand offers protein information for 260 chaperones, but has limited variety concerning different cancer groups: it comprises only ovarian, colorectal and breast cancer samples, among which only the latter overlaps with cancers relevant for our study.

For the examination of the relation between gene and protein expression in TCGA breast cancer samples and its effect on the results of our study, we first calculated and compared the Spearman correlation for each gene and its protein product (Fig A).

Fig A. Correlation between BRCA gene and protein expressions, from TCGA and CPTAC respectively, and their difference in chaperome vs, whole genome.

Based on the results depicted in Fig A, we argue that while the correlation between RNA and protein abundance is not perfect in either group of genes, it is fair to assume a positive correlation between genes and proteins in TCGA Breast cancer samples. Moreover, chaperones have a significantly higher correlation between gene expression and protein abundance than the whole genome (${p value}_{t-test} < {10}^{-8}$). Therefore, it is suitable to use RNA-seq data instead of protein expression for our study.

As a next step, we studied the effect of using protein expression as input for gene set analysis (GSA): after calculating the alteration of chaperone groups in breast cancer using protein abundance, we compared the outcome to results of our paper, which are based on RNA-seq (Fig. B). Overall we observed a Pearson correlation of 0.53 between results based on gene and protein expression, which we deemed acceptable as another justification for using gene expression in our study.

Fig B. Set enrichment analysis on protein vs. gene expression.
